# StaVia: spatially and temporally aware cartography with higher-order random walks for cell atlases

**DOI:** 10.1186/s13059-024-03347-y

**Published:** 2024-08-16

**Authors:** Shobana V. Stassen, Minato Kobashi, Edmund Y. Lam, Yuanhua Huang, Joshua W. K. Ho, Kevin K. Tsia

**Affiliations:** 1 Department of Electrical and Electronic Engineering, The University of Hong Kong, Pokfulam, Hong Kong, Hong Kong; 2 School of Biomedical Sciences, Li Ka Shing Faculty of Medicine, The University of Hong Kong, Pokfulam, Hong Kong; 3 Advanced Biomedical Instrumentation Centre, Hong Kong Science Park, Shatin, New Territories Hong Kong; 4 Laboratory of Data Discovery for Health, Hong Kong Science Park, Shatin, New Territories Hong Kong; 5 Department of Statistics and Actuarial Science, The University of Hong Kong, Pokfulam, Hong Kong; 6 AI Chip Center for Emerging Smart Systems, Hong Kong Science Park, Shatin, New Territories Hong Kong

## Abstract

Single-cell atlases pose daunting computational challenges pertaining to the integration of spatial and temporal information and the visualization of trajectories across large atlases. We introduce StaVia, a computational framework that synergizes multi-faceted single-cell data with higher-order random walks that leverage the memory of cells’ past states, fused with a cartographic Atlas View that offers intuitive graph visualization. This spatially aware cartography captures relationships between cell populations based on their spatial location as well as their gene expression and developmental stage. We demonstrate this using zebrafish gastrulation data, underscoring its potential to dissect complex biological landscapes in both spatial and temporal contexts.

**Supplementary Information:**

The online version contains supplementary material available at 10.1186/s13059-024-03347-y.

## Background

The recent surge in the creation of single-cell atlases has ushered in a new era of understanding the complexities of life at the cellular level. These atlases are now instrumental for studying a wide range of tissues, organs, and even whole organisms to reveal the origins of cellular differentiation and functional diversity [1, 2]. Further combined with spatial and time-series studies, they offer a high-definition window into biological development over space and time [3–7]. However, the growing scale of single-cell atlases often poses daunting analytical challenges [8, 9]. Specifically, the elevated complexity of large-scale atlases in terms of heterogeneity, temporal longitude, spatial environments, and sample sizes makes it difficult to unambiguously capture the emergence of multiple specialized cell lineages and their differentiation pathways at a high resolution, not to mention the difficulty of intuitively visualizing these complex pathways at this scale.

Available TI methods face three pressing challenges, the first is the inability to resolve end-to-end differentiation pathways that preserve localized details of underlying trajectories while maintaining a global view of their connectivity, resulting in differentiation pathways for distinct lineages being intermingled (by deviating into unrelated intermediate cell populations) or too myopic (failing to detect transition states). The use of first-order memoryless random walks employed by most TI methods (e.g., Palantir [10], MARGARET [11], CellRank [12], Via 1.0 [13]) makes them particularly susceptible to these misleading pathways. The second is that strategies to integrate available metadata (e.g., spatial or temporal information) that could aid in the analysis of the cellular landscape are not readily available, thus forgoing the opportunity to use these sources of complementary information. Third, current practices to visualize developmental landscapes rely on established dimension reduction visualization tools which primarily capture clusters of distinct lineages (e.g., UMAP [14], t-SNE [15]). These tools are not designed to display a single-cell embedding that can intuitively be mapped or linked to inferred continuous trajectories. On the other hand, methods relying on diffusion maps (e.g., Phate [16]) convey progression information at the expense of collapsing/superimposing multiple distinct lineages.

**Fig. 1:**
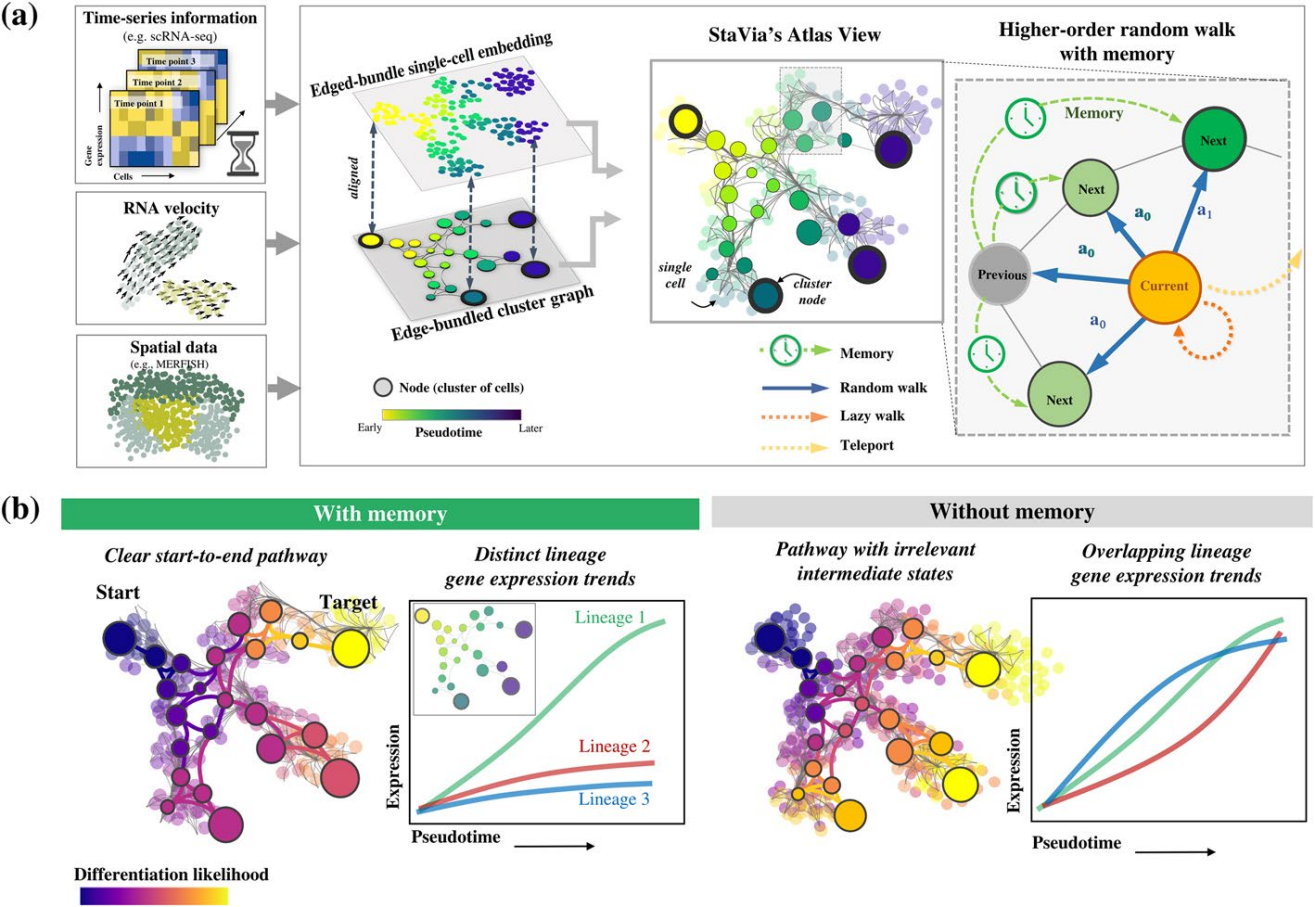
Overview of StaVia. **a** The StaVia graph is a flexible framework for single-cell data that can optionally incorporate any combination of the following data to infer cell transitions: sequential or spatial metadata (e.g., known stages, tissue coordinates), RNA-velocity, pseudotime, and lazy or teleporting behaviors. Based on an algorithm of higher-order lazy-teleporting random walks (LTRW) with memory, StaVia can generate single-cell embeddings with the underlying high-resolution connectivity of the single-cell KNN graph. This can be aligned with an edge-bundled cluster graph in which each node represents a cluster of single cells. The cluster graph and single-cell embedding can be overlaid to generate an Atlas View which offers an intuitive and comprehensive visualization of the computed trajectories. StaVia uses higher-order LTRW with memory to accurately infer complex trajectories. Previous states’ neighbors inform the decision-making process for subsequent transitions, i.e., to determine the transition probabilities of moving from the current state to the next states by introducing a memory factor **a** (“Methods”). **b** The Atlas View allows us to cartographically observe end-to-end pathways at a higher resolution. Higher-order LTRWs with memory ensure that pathways avoid detours to unrelated cell types and hence also increase the specificity of gene regulation along distinct lineages

To address these challenges, we present StaVia, an automated end-to-end trajectory inference (TI) framework that uncovers cellular trajectories permeating large-scale single-cell spatial and temporal atlases without sacrificing fine-grained details. To address the first obstacle, StaVia exploits a new form of lazy-teleporting random walks (LTRW) *with memory* to accurately pinpoint end-to-end trajectories in the atlas. Specifically, higher-order LTRW with memory are used to propagate information about a cell’s previous states when inferring subsequent states (e.g., during differentiation) (Fig. 1a). The inclusion of memory of past states critically alleviates issues seen in traditional first-order *memoryless* RW methods where pathways deviate into unrelated intermediate cell populations, or conversely become so localized that they fail to detect transition states (Fig. 1b). Secondly, StaVia’s framework is also flexibly compatible with diverse input data types; in addition to RNA velocity, it offers seamless strategies to integrate spatial coordinates and temporal information (or other sequential metadata) to guide the cartography in a data-driven manner (Fig. 1a).

To address the third challenge of visually capturing complex TI landscapes, StaVia feeds forward the properties of the higher-order walks with memory and metadata to create a comprehensive cartographic *Atlas View*, which efficiently (both in terms of spatial layout and computational cost) integrates the high-resolution graph-edge information with the cell type specificity of single-cell embeddings to visually chart the predicted trajectories of entire atlases in a unified snapshot (Fig. 1a). As a result, StaVia can simultaneously capture smooth sequential processes while maintaining the separation of distinct lineages—outperforming popular visualization tools (t-SNE, UMAP, Phate, etc.).

We use a murine gastrulation atlas [6] and the recent zebrafish developmental atlas (Zebrahub) [7] to show how the incorporation of second-order LTRWs with memory, together with sequential information and RNA-velocity in StaVia, allows us to compute and visualize multi-lineage differentiation in atlases, and capture pathways that cannot be charted by other methods. We also demonstrate StaVia’s spatially aware cartography on a MERFISH dataset of the preoptic hypothalamus [17] and a spatio-temporal zebrafish gastrulation atlas ZESTA [18], where StaVia integrates information of a cell’s spatial context with gene expression, to uncover sub-types and inter-cluster relationships that can only be captured when using the spatial information. Finally, a collection of developmental cell atlases is used to benchmark the Atlas View to other popular visualization methods, in which StaVia is the only method that successfully illustrates the temporal and lineage relationships in an 8-million-cell dataset of mouse gastrulation [9].

## Results

### StaVia enables high-definition cartographic TI reconstruction across the entire single-cell atlas

StaVia is a graph-based TI framework designed to tackle challenges posed by atlas-scale data. It builds on our earlier VIA method [13] which models cellular processes as a random walk with elements of laziness and teleportation across cluster graphs [19]. In StaVia, we introduce higher-order walks with the memory of cells’ previous states, integrated with cartographic views and enriched with information from available metadata (temporal or spatial), to reconstruct atlas-scale topologies coupled with automated predictions of diverse cell fates and their sequential specialization.

Advancing from VIA and other TI methods, StaVia’s contributions are threefold. First, StaVia uses high-order LTRWs with memory to infer complex trajectories by relaying information about a cell’s previous states (Fig. 1b). This approach accurately pinpoints end-to-end differentiation paths and gene dynamics associated with a particular lineage. Forgoing the walk’s memory can obscure the distinction between the different pathways to cell fates in large atlases. Second, it allows flexible integration of data and metadata (e.g., time-series developmental labels from temporal atlases, spatial layout, gene/feature similarity, and single-cell RNA-velocity) to compute pseudotimes, cell fates, and lineage pathways (“Methods”) (Fig. 1a). Integrating available temporal data with the expression profiles allows us to stitch developmental points in a data-driven manner. Spatial information is particularly challenging to incorporate when examining cellular landscapes based on their gene expression due to their highly non-linear nature. However, the microenvironments that cells occupy could provide valuable insight about their function. StaVia therefore provides a framework within which gene-expression and spatial information are jointly considered when charting the cellular landscape. Lastly, going beyond the common cluster graph visualization [13, 20], StaVia generates an *Atlas View* that simultaneously illustrates complex chronological patterns and distinct phenotypic diversity, which has been challenging in current TI methods. Both the spatial arrangement of nodes and edges in StaVia’s high-resolution *Atlas View* (and its cluster graph), as well as their direction, connectivity, and weights, are guided synergistically by the results of the pseudotime, sequential metadata, and second-order LTRWs (“Methods”) (Fig. 1a).

Using higher-order walks with memory is an unexplored feature in existing TI methods which typically rely on first-order random walks where the prediction of future steps is independent of previous states (e.g., Palantir [10], MARGARET [11], CellRank [12], and Via 1.0 [13]. When applied to reconstructing biological pathways, memoryless methods tend to encounter two types of problems which we explain by way of analogy to a faulty navigation system on a road trip from City A to B. The first issue occurs when memoryless methods suggest a cell passes through an unrelated intermediate population during development, akin to a GPS diverting us through an off-route City C. StaVia, uses higher-order LTRW with memory to act like an improved GPS that sense-checks directions, minimizing unnecessary detours, and ensuring a more accurate cell trajectory. Now imagine the road trip involves a critical turn at Point D but the GPS is so focused on the immediate road that it misses this turn. Analogously, some TI methods, due to an overemphasis on localized pathways, may fail to identify key transition states in a cell’s developmental journey. StaVia’s “memory” feature is like an alert GPS that not only focuses on the road immediately ahead but also keeps track of the overall journey. It remembers where each cell has been and where it could be headed, making it less likely to miss critical turns (or transition states), and providing a more complete picture of the cell’s developmental journey. By integrating pseudotemporal forward biasing, RNA velocity, and prior random walk state information (memory), StaVia’s higher-order LTRWs provide a more realistic prediction model of cell developmental pathways, enabling clear delineation of diverse lineages, transitional populations, and gene expression dynamics. (Fig. 1b).

Our robustness analysis shows that adjusting the memory level has a predictable and gradual impact on lineage definitions, simplifying the optimization (see “Methods” and Additional file 1: Fig. S5). Generally, increasing emphasis on memory in the LTRWs yields pathways that emphasize the role of predecessors and remain inwardly focused. This translates to increased sensitivity to distinguishing related cell types and their gene expression dynamics (Fig. 1b). Conversely, reducing memory helps explore poorly connected cell populations or those lacking precursors. The computational overhead from second-order LTRWs is minimal as they are conducted on the cluster graph level.

To generate StaVia’s cartographic *Atlas View*, we first create a single-cell embedding infused with second-order LTRW features learned from the TI cluster graph (see “Methods”). Specifically, based on the presence of sequential data (e.g. data labeled with different time points), the single-cell graph can be sequentially augmented and refined accordingly. In the case of spatial data, spatial nearest neighbors are used to augment the gene expression graph. Furthermore, prior to clustering and graph construction, the gene expression is modified as the weighted average of a cell’s own cells and its spatial neighbors when spatial data is being considered. We then use UMAP’s fuzzy simplicial set approach to align the LTRW-based cluster graph with a low-dimensional embedding (Fig. 1a). This single-cell embedding, a useful visualization in its own right, serves as the node layout for the Atlas View which arranges cell states and highlights edge connectivities (pathways) from the (spatial-temporally) augmented single-cell graph using an edge bundling method based on kernel density estimation (“Methods”). Directionality is projected on edges based on milestone pseudotime direction and RNA velocity. Note that the impact of high-order LTRWs with memory on the predicted end-to-end pathways can also readily be visualized in the Atlas View (Fig. 1b). For a detailed guide to parameter selection, see Additional file 1: Note S1.

### StaVia captures a complete view of murine gastrulation

**Fig. 2:**
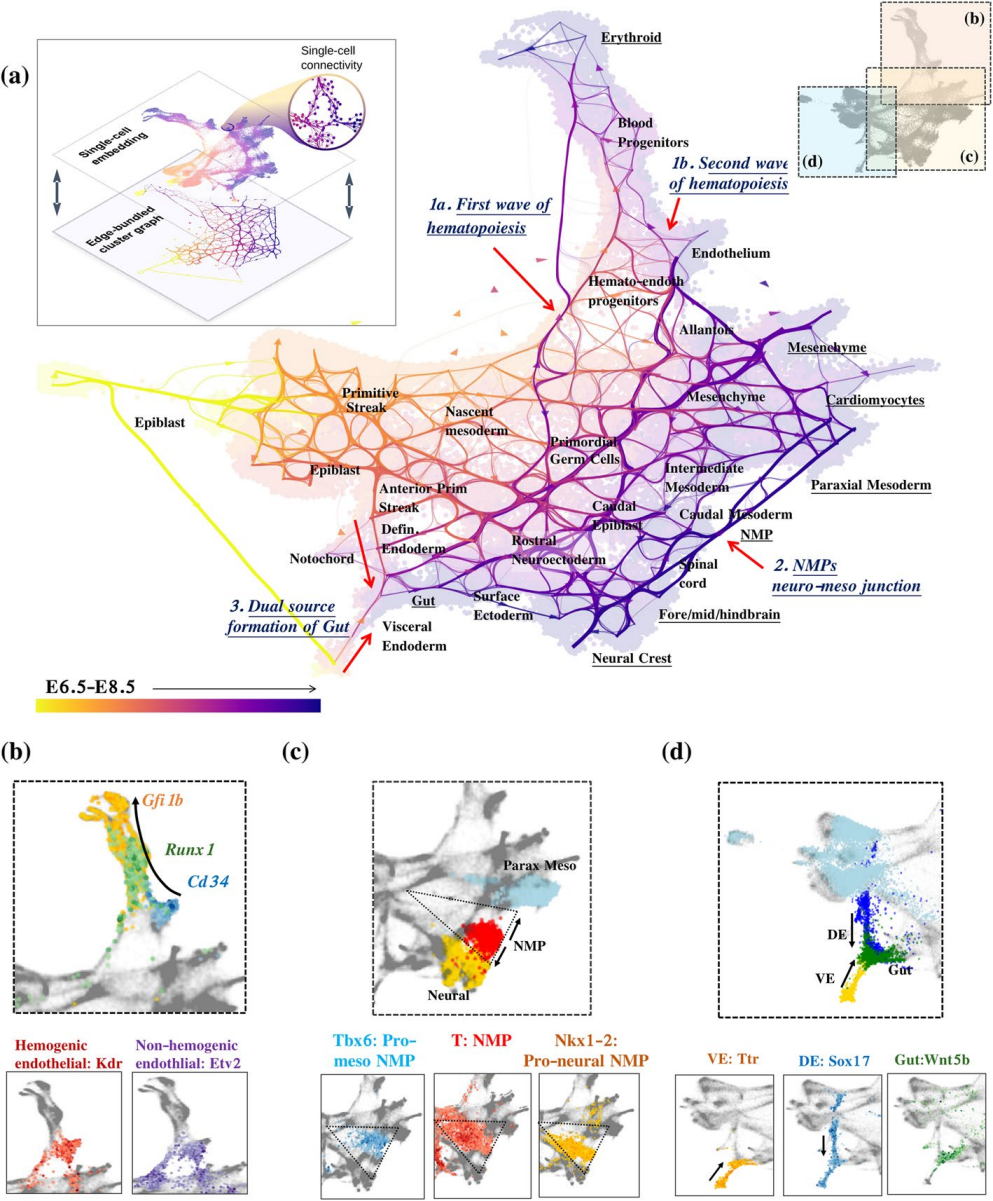
StaVia Atlas View of mouse gastrulation. **a** StaVia Atlas View of murine gastrulation, colored by known stage with edge directions inferred using a combination of RNA velocity and pseudotime. Root state automatically detected as epiblast E6.5. Autodetected terminal cell fates are underlined. **b** Sequential order of hemogenic endothelial cell differentiation. The black arrow is based on the edge direction of the hematopoietic branch in **a** and shows that Runx1 precedes the upregulation of GFi1b, which is a direct target of Runx1 and critically down-regulates endothelial markers to induce the endothelial-to-hematopoietic transition (EHT) [21, 22]. **c** NMP cells colored red reside between neural-yellow and paraxial mesoderm-blue (lhs) the zoomed-in triangle of NMP cells express T brachyury. Of interest, the NMPs with a mesodermal tendency express *Tbx6*. NMPs with a more neural tendency express more *Nkx1-2* [23]. **d** Dual source of gut formation with *Ttr*-positive cells at the visceral endoderm (VE), *Sox17* expression for definitive endoderm (DE), and gut expressing *Wnt5b* [24, 25]

**Fig. 3:**
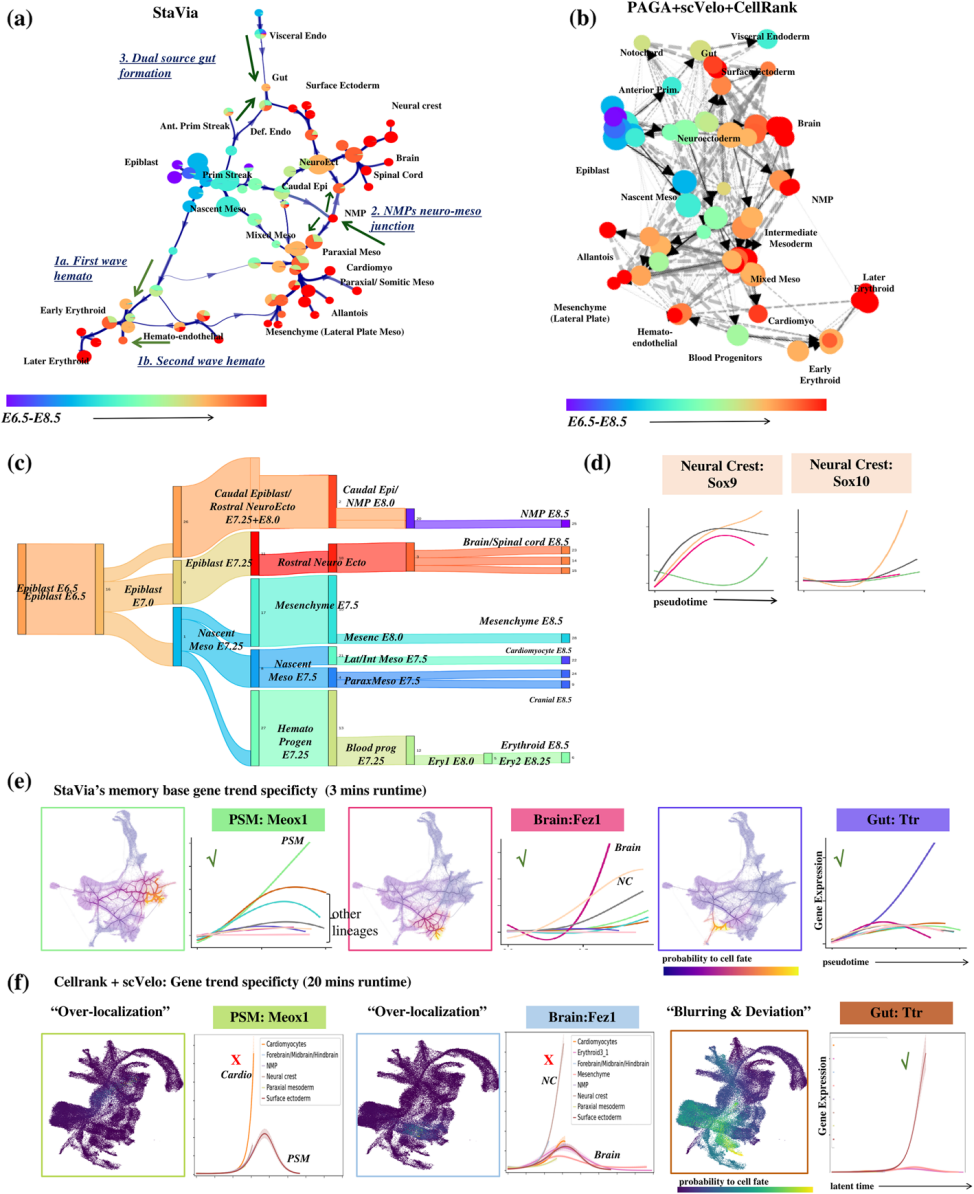
Comparison of TI graph structure and analysis. **a** StaVia cluster graph shows a directed trajectory using a combination of scRNA-velocity and pseudotime. Colored by known stage within the mesoderm, StaVia identifies cardiomyocytes, paraxial mesoderm, and mesenchymal cells; within the neuro-ectodermal branch: the surface ectoderm, brain, and neural crest (NC); and arising from the visceral and definitive endoderm, the gut. **b** scVelo directed PAGA with a similar number of clusters and also using force-directed layout—lower visualized edges results in several disconnected clusters. **c** Automatically predicted differentiation flow based on the cluster graph. **d** StaVia captures *Sox9* upregulation preceding *Sox10* in Neural Crest (NC) development. **e** StaVia end-to-end pathways from epiblast to cell fate for each germ layer. Each trend line corresponds to a lineage. The lineage of interest is highlighted by the color of the lineage-plot’s border and associated marker-gene, e.g., in StaVia the brain lineage is dark pink. When the color of the marker gene matches the color of the upregulated trend line, it signals that the correct trend is inferred and merits a checkmark. **f** CellRank: the lineage pathways to the brain (light blue) exhibits is an example of where the pathway fails to detect transition states due to over localization and the corresponding blue lineage trend is not upregulated, warranting a cross-mark. The gut pathway (brown trendline) is an example of deviation into unrelated intermediate states resulting in distinct pathways becoming blurred

We employed StaVia on a scRNA-seq dataset of murine gastrulation [6], comprising 89,297 cells from stages E6.5 to E8.5 post-fertilization at quarter-day intervals. Previous trajectory analysis on this dataset required subsetting various lineages and analyzing them individually with manual curation in order to identify developmental trajectories of interest. In contrast, StaVia, by integrating higher-order LTRW with memory, RNA velocity, and time-series annotations (i.e., E6.5 to E8.5), accomplishes a holistic mapping of the entire atlas in a single run, accurately capturing relevant trajectories sans manual subsetting and curation (Figs. 2 and 3).

The Atlas View (Fig. 2a) and the cluster graph (Fig. 3a) visualizations created by StaVia illustrate the emergence of lineages within the entire dataset through a fanned-out structure that reflects the increasing separation between progressively specialized cells. The cluster graph (Fig. 3a), which forms the basis for the memory-infused second-order LTRW lineage probabilities as well as the layout and directionality of the Atlas View, captures the emergence of major lineages, their progressive separations towards cell fates (highlighted on the Atlas View Fig. 2a as underlined populations), and the correct placement of more subtle transition populations that exist at the boundaries of these major layers (e.g., neural mesodermal progenitors (NMPs) [23]). Interestingly, both the cluster graph and Atlas View are uniquely able to show that the gut arises from the visceral and definitive endoderm [24, 25]. They also visually indicate two hematopoietic sources, the first being erythroids from the primitive wave and the endothelial cells, which suggest the onset of the second wave (Figs. 2a, b and 3a) [26]. This structure is not easily observed in other cluster graphs (e.g., PAGA Fig. 3b) or in higher resolution representations (e.g., UMAP, Phate), as evidenced by the comparative visualization analysis presented later (Fig. 8). See Additional file 1: Note S2 for details of these three developmental patterns as shown by the StaVia cluster graph and Atlas View and Additional file 1: Table S3a for a full list of supporting literature.

Next, we compared StaVia with a hybrid pipeline involving PAGA [20], scVelo [27], and CellRank [12], a state-of-the-art method that combines gene–gene feature distances with directional information from RNA velocity for cell fate determination (see Additional file 1: Note S3 for details on the selection of benchmarked methods). Comparing StaVia’s cluster graph with PAGA’s (using CellRank’s initial states and scVelo’s RNA velocity [27]) (Fig. 3b) (see Additional file 1: Table S1 for detailed parameter setting), we observe that the PAGA-scVelo plot is visually difficult to interpret due to edge congestion that cannot easily be minimized. This is due to even conservative attempts of edge thresholding resulting in graph fragmentation. Importantly, the connectivity in the PAGA-scVelo plot misses key biological insights (e.g., lacks dual source of gut formation).

We subsequently compared the lineage probabilities from StaVia and CellRank towards different cell fates (Fig. 3e–f and Additional file 1: Fig. S3c), with StaVia completing the TI computation in 3 min, compared to CellRank’s 20 min (Additional file 1: Note S3). Notably, the single-cell probabilistic lineages in CellRank do not capture the end-to-end pathways from epiblast, through transition states to final cell fates. In most cases for CellRank, the lineage probabilities (Fig. 3f and Additional file 1: Fig. S3 for all lineages) are either very localized to cells at the corresponding final cell fate with no indication of past states (NMP, brain, presomitic/paraxial mesoderm (PSM)) or are very diffuse detouring through the entire landscape (gut, cardiomyocyte) falsely suggesting that unrelated intermediate cells have a high likelihood of differentiating towards these cell fates. The same issues of either blurring or over-localization during pathway prediction are observed in Palantir, with manual setting of cell fates required to bypass incorrect automated predictions in Palantir as demonstrated in Additional file 1: Fig. S3, Fig. S7 and Fig. S19). In contrast, the graph structure presented by StaVia (Fig. 3a) and its LTRW traversal using memory enables us to more unambiguously retrace how these lineages emerge (Fig. 3a, c, e and Additional file 1: Fig. S3).

At higher memory levels, the gene trends predicted for the brain lineage show distinct elevation of *Fez1* and *Pax6*, crucial for neuroectoderm fate specification, neurogenesis, and forebrain patterning (Fig. 3e, Additional file 1: Fig. S4) [28, 29]. StaVia also reveals a noteworthy trend: *Sox9* expression precedes *Sox10* in neural crest (NC) precursors (Fig. 3d). This aligns with known data showing *Sox9*’s role in initiating premigratory NC cells, followed by *Sox10*, which fosters later NC development and cell emigration [30]. This *Sox9*-*Sox10* sequence is not captured by CellRank (Additional file 1: Fig. S3).

### Introducing memory in random walks delineates end-to-end pathways in murine gastrulation

**Fig. 4:**
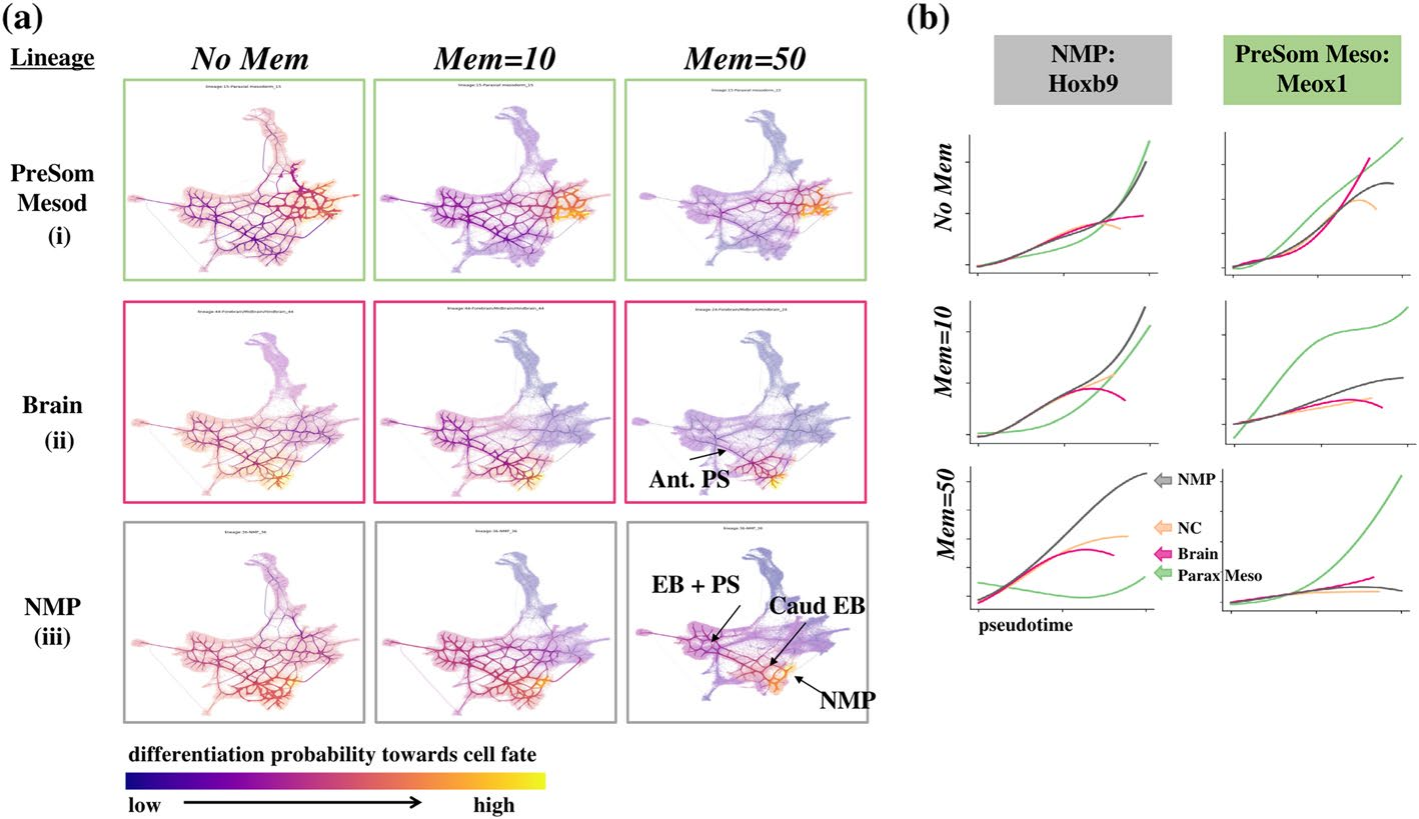
StaVia Memory impact on lineage paths. **a** Increasing memory mitigates too-diffused pathways from epiblast towards specialized cell fates and consequently improves the associated gene trends specificity as shown in **b**. Gene expression trends along pseudotime for lineages NMP (grey), PSM (green), brain (pink), and neural crest (NC) (peach). *Hoxb9* is an NMP marker and we expect the grey NMP gene expression to become comparatively more upregulated than the other three lineages. Similarly, we expect *Meox1* as a PSM marker to be comparatively more upregulated

We next investigated how the incorporation of memory into random walks improves cell fate mappings and their associated biological interpretation, by addressing the issue of too-localized or too-diffused paths seen in current methods. In the murine gastrulation dataset (and later Zebrahub), we compared the lineage pathways obtained using a first-order and second-order LTRW with varying levels of memory (Mem = 1 (signifies no memory) to Mem = 50). Lower memory values lead to more diffuse pathways on StaVia’s Atlas View (Fig. 4 and Additional file 1: Fig. S4), confounding analysis of temporal gene dynamics. In contrast, higher memory values successfully distinguish adjacent cell fates, as shown by the temporal gene expression of the NMP, paraxial mesoderm, neural, and neural crest cell fates at E8.5 (Fig. 4a-b).

For the NMP lineage, nestled between the emerging PSM and neural cells, higher memory enables the delineation of the sequential cell pathway from the epiblast to caudal epiblast and then to the boundary of the mesodermal and neuronal lineages for the biopotent NMP cell fate (Fig. 4a-iii). In contrast, lower memory values tend to include unrelated cell populations. As a result, gene expression trends for NMP markers *Hoxb9* and *Nkx1.2* [23] overlap for all these lineages at lower memory values, but at memory = 50, the NMP lineage alone shows distinct expression elevation (Fig. 4b, Fig. S4). The benefits of the memory mechanism are also evident in the E8.5 brain cells (Fig. 4a-ii), where higher memory more accurately shows the brain lineage deriving from the epiblast, followed by cells in the anterior pole of the primitive streak [31].

### StaVia displays holistic and high-resolution transcriptomic landscape of the full Zebrahub

**Fig. 5:**
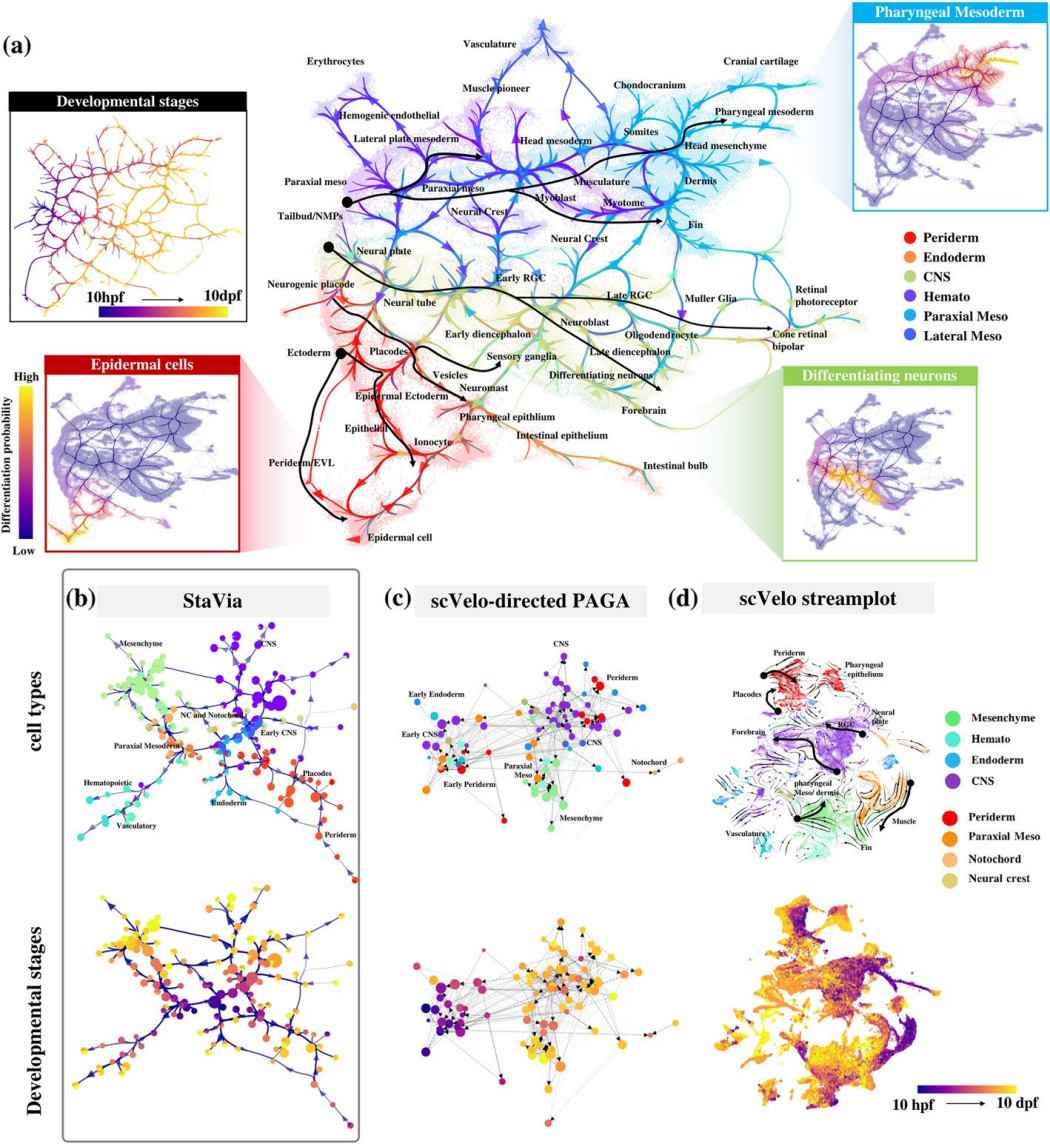
StaVia for Zebrahub. **a** Atlas View of the entire Zebrahub bud stage to 10 dpf colored by germ layer. Black arrows highlight the direction of differentiation indicated by Atlas edges for major lineages in the mesoderm, neuro-ectoderm, and non-neuro ectoderm. (Insets) StaVia end-to-end paths from bud to cell from mesoderm, neuro-, and non-neural ectoderm show well-delineated pathways as a result of higher-order random walks. **b** StaVia directed cluster graph using scRNA-velocity and pseudotime colored by main tissue type (top) and known stage (bottom). Edge directions radiate outwards from the center. **c** scVelo-directed PAGA constructed with a similar number of clusters as StaVia and also using a force-directed layout, shows a congested edge-layout with tissue-specific groups poorly separated and no clear direction. (d) scVelo stream plot on UMAP cannot mark the emergence of lineages as clearly as the edges in the Atlas View. The black arrows trace similar lineages to those highlighted in the Atlas but do not transition through intermediate stages and often show conflicting direction

We proceeded to leverage StaVia to probe the full Zebrahub, a recent comprehensive scRNA-sequencing time course atlas of 120,000 zebrafish embryonic cells [7] (Fig. 5a). As current methods struggle to reconcile the extended temporal span and extensive cellular information of the entire 10-hpf to 10-dpf (hour/day post-fertilization) dataset, Lange et al., limited their study to the subset of cells (only 30% of the cells in the time-course study), omitting the peridermal and neuroectoderm lineages entirely. We show that StaVia successfully interrogates the complete dataset. Notably, Zebrahub’s neuroectoderm and periderm lineages are analyzed here for the first time with an example of the probabilistic pathway from each of these three layers (see the insets in Fig. 5a).

**Fig. 6:**
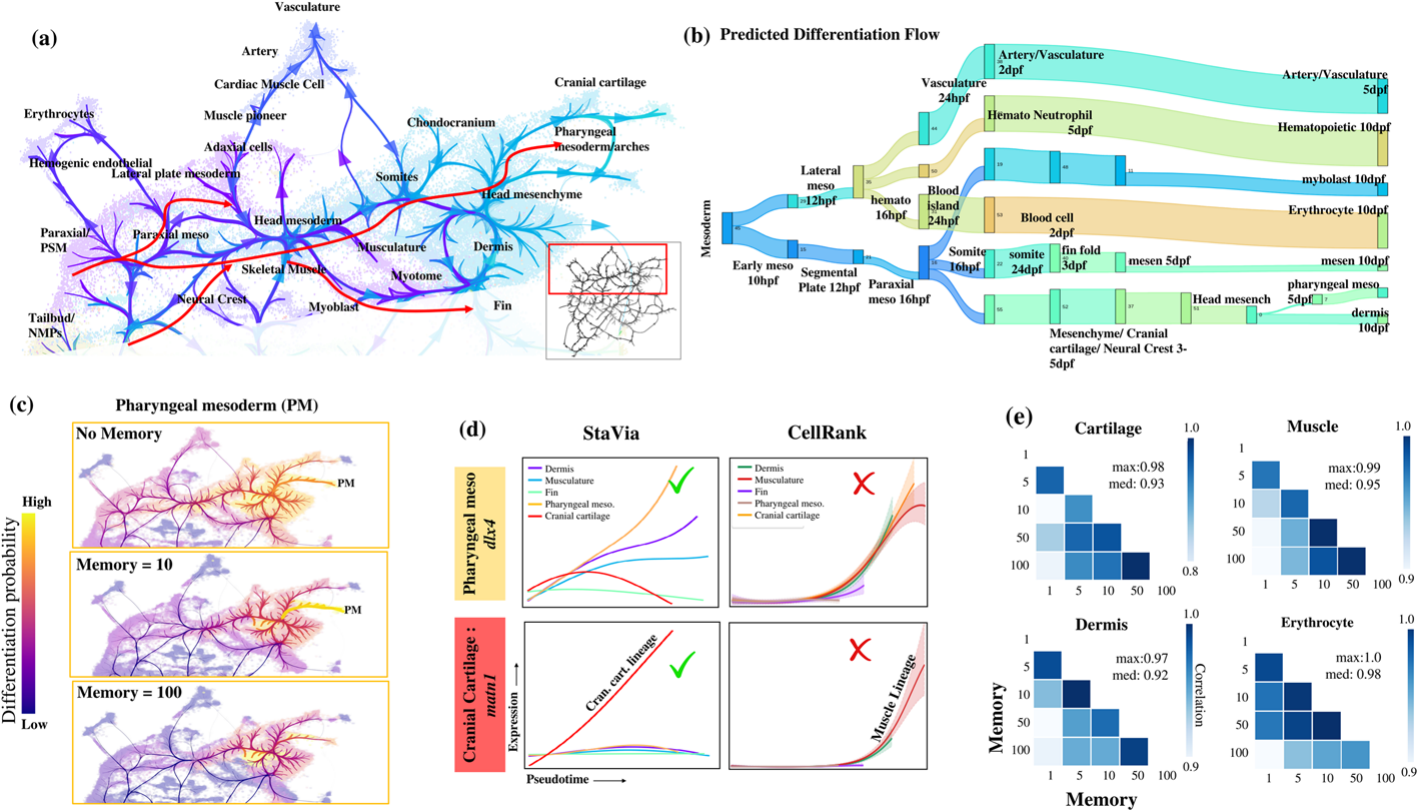
Mesoderm development. **a** Zoom-in of mesodermal lineage highlighting paths to pharyngeal mesoderm and musculature. **b** Automated predicted differentiation flow of detected mesodermal and hematopoietic cell fates from early mesoderm 10 hpf to 10 dpf. **c** In StaVia, increasing memory shows clearer paths to cell fate of interest and avoids spillover into unrelated cell types. **d** Gene trends of mesoderm lineages for dlx4 (pharyngeal mesoderm marker) [32] and matn1 (cranial cartilage marker) [33] are correctly captured by StaVia, whereas CellRank’s lineages are incorrect (e.g., the muscle lineage upregulates matn1 in CellRank). **e** Correlation matrices for cartilage, muscle, dermis, and erythrocyte lineage pathways at different values of memory 1 to 100 shows stability of analysis when changing memory (Fig. S7 for all fates)

**Fig. 7:**
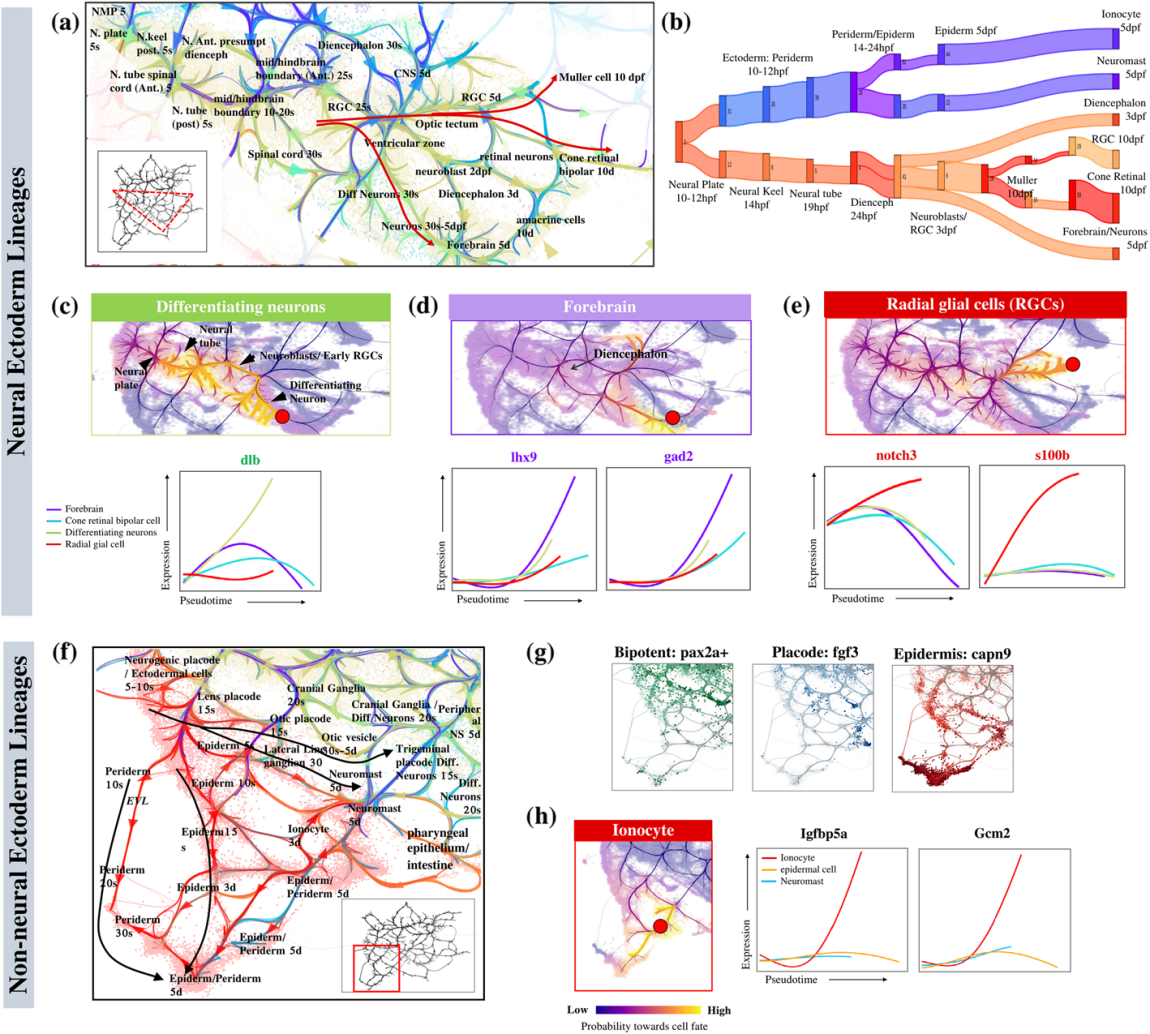
StaVia reveals Ectoderm differentiation in Zebrahub. **a** Zoom-in Atlas View of neurulation from 5-hpf to 10-dpf. “s” denotes somite-stage and “d” is days post fertilization (dpf). **b** Predicted differentiation flow of non-neural ectoderm and neural fates. **c**–**d** End-to-end pathways from neural plate region at 10 hpf (5-somite) to **c** differentiating neurons and **d** forebrain (5–10 dpf). Accompanying gene-expression trends for neural lineages, shows upregulation of marker genes. **e** RGC end-to-end pathway and its marker gene expression trends. **f** Zoom-in Atlas View of non-neural ectoderm regions shows the formation of bilayered epiderm and the differentiation of the placodes and their interactions with associated trigeminal neurons/ganglia. **g** Pax2a expressed in the early epiderm and placode bipotent regions, *Fgf3* restricted to placodes, and *Capn9* concentrated on epidermal cells. **h** StaVia detects that the ionocyte fate (red dot) expresses more *Igfbp5ag* and *Gcm2* than other non-neuro ectoderm lineages

StaVia outperforms existing state-of-the-art methods, e.g., scVelo, in delineating the intricate cell differentiation trajectories. For instance, StaVia recapitulates that cross-talk between the major lineages (visualized as edges on both the Atlas View (Fig. 5a) and cluster graph (Fig. 5b)) is more prominent during earlier stages (e.g., neuro mesodermal progenitor pluripotent cell types like (NMPs) traverse two germ layers) and diminishes as cells become more specialized. Furthermore, the direction of differentiation is also more clearly captured by StaVia’s cluster graph (Fig. 5b) than CellRank-scVelo-directed PAGA representation (Fig. 5c) and scVelo’s stream plot (bold black arrows in Fig. 5d). StaVia also detects more relevant late-stage cell fates (Figs. 6b and 7b), as well as the gene trends that distinguish these lineages from each other (see Additional file 1: Figs. S7-S9 for lineage comparisons on all detected cell fates, where those missed by CellRank are manually provided to allow comparison), avoiding the pitfalls of missing transition states and inconsistent directionality occurring in the scVelo stream plot (Fig. 5d). Again, StaVia’s TI runtime is fast, taking 4 min, compared to 30 min in CellRank which misses several cell fates (Additional file 1: Fig. S6).

### StaVia distinguishes multiple mesodermal pathways in Zebrahub

StaVia uncovers a high-precision mesodermal differentiation flow (Fig. 6a-b) that cannot be recovered from the scVelo and PAGA maps. It accurately predicts that vascular and hematopoietic lineages are derived from the lateral plate mesoderm [34] while revealing that the paraxial mesoderm gives rise to somitic cells, precursors to the dermis and cartilage [35]. Critical to early embryonic development, bipotent NMPs located at the early bifurcation of the mesoderm and neuroectoderm are identified [36] (Figs. 5a and 6a). Again, this ability is attributable to the memory-centric graph traversal implemented in StaVia (see the impact of the memory on mesodermal differentiation analysis in Figs. S10-11).

Notably, StaVia accurately predicts that the pharyngeal arch is derived from the head mesoderm and the cranial neural crest [37, 38] (see the zoom-in Atlas View in Fig. 6a, differentiation flow in Fig. 6b and pathway in Fig. 6c). The upregulation of *Dlx* genes, as revealed by StaVia, marks the emergence of a pharyngeal population (Fig. 6c–d). This is in contrast to CellRank, which, lacking the memory mechanism, fails to distinguish *Dlx* expression patterns across mesodermal lineages, resulting in a homogenized expression that masks true cell fate distinctions (Fig. 6d, Additional file 1: Fig. S7 where cell fates missed by CellRank are manually assigned to allow full comparison). Furthermore, StaVia identifies Matrilin *Matn* as a gene marker to distinguish the cranial cartilage from the pharyngeal arches (Fig. 6d) [33]. This distinction is lost in CellRank (Fig. 6d), which confounds the cartilage with smooth musculature. CellRank’s paths lack intermediate populations (Additional file 1: Fig. S7), showing either fate-localized lineage probabilities or diffuse pathways (Additional file 1: Fig. S6).

While increasing the memory for graph traversal can generally sharpen the specificity of lineage progression towards the desired cell fate (Fig. 6c and Figs. S10-11 for other cell fates), excessive memory can constrain the pathway, as seen with the PM lineage at Mem = 100 (Fig. 6c), underscoring the need for a balanced application of this parameter. Our stability analysis (Fig. 6e and Additional file 1: Fig. S5) indicates that adjusting memory has a predictable and controllable impact. A heuristic correlating known time-series labels with pseudotime across memory values aids in determining the optimal memory range, ensuring the accuracy of inference by StaVia (see “Methods” and Additional file 1: Fig. S5).

### StaVia elucidates neurulation sequence and differentiation of radial glia

We analyzed neuro-ectodermal lineages in Zebrahub (Fig. 7a-e) using StaVia, marking the first analysis of these cells which have otherwise been omitted in prior analyses. It identifies four distinct cell fates in the 5- and 10-dpf neuronal branches: forebrain cells, radial glia, and differentiating neuron and cone retinal bipolar cells. This contrasts with CellRank’s identification of only the bipolar cells and radial glial cells (Additional file 1: Fig. S8 for the full set of cell fates). Crucially, StaVia successfully traces the neurulation sequence, from the neural plate through the neural tube cells’ progression to the diencephalon, and culminating in the mature forebrain neurons [39] (Fig. 7a-b). Again, the use of memory enables us to identify gene expression trends specific to these cell fates. For instance, differentiating neurons are distinguished by the upregulation of *Delta* genes (*Dla/Dlb*) specific to the subventricular zone (Fig. 7c) [40], whilst the mature post-mitotic neurons of the forebrain have elevated *lhx9* and *gad2* (Fig. 7d) CellRank’s diffuse probabilities of the neurons blur gene trends, confusing differentiating neurons with other ectodermal fates (Additional file 1: Fig. S8) [40].

The zoomed-in Atlas View (red arrows in Fig. 7a) and probabilistic pathways (Fig. 7e) uniquely highlight how the multipotent radial glial cells (RGCs) give rise to both neurons and glia. They are characterized by activated *notch3* and *s100b*, indicative of early gliogenesis [41–43]. StaVia further captures that the early RGCs are partially diverted to the differentiating neurons through a neuroblast sub-branch, while the other RGCs continue to the 10-dpf state where they differentiate (indicated by minor sub-branches) into Muller glia, oligodendrocytes or neurons (Fig. 7a).

### StaVia charts emergence of bilayered epidermis and placodes from Pax2 + field

StaVia’s Atlas View clearly separates the neural and non-neural ectoderm, enabling for the first time an unsupervised analysis of the Zebrahub non-neural lineages in the ectoderm (Fig. 7e-g). The identified edge connectivities capture how the ectodermal field of bipotent *Pax2*+ cells give rise to both the *Fgf3*+ otic placode and *Capn9*+ epidermis [44] (Fig. 7f).

The high-resolution edges of the Atlas View present the emergence of otic placodes which later yield the otic vesicles (Fig. 7e black arrows) [45]. StaVia’s ability to capture localized details within a more global network is seen in the correct placement of the neuromasts (uniquely upregulating *Fndc7a* [46]) along the lateral line placode, with edges to the neuronal cranial ganglia population known to innervate them [47]. The formation of an early bilayered epidermis (Fig. 7e) from the extraembryonic enveloping layer (EVL)/periderm, and the inner basal epidermis is also detected by StaVia. The epidermal cells are identified by markers *Capn9, Anxa1b/c* in StaVia (Fig. 7f, Additional file 1: Fig. S9) [44]*,* but in CellRank are indistinguishable from neuromasts and ionocytes due to diffuse lineage probabilities (Fig. S9).

Notably, StaVia detects two small cell fates each comprising less than 0.3% of the cell atlas. One comprises cells in the pharyngeal epithelial lining (located towards the lower right of Fig. 7e) which are formed by peridermal cells invading the pharyngeal cavity and subsequently expanding along the midline until the esophagus-gut boundary [48]. The second is the ionocyte cell fate (expressing *Igfbp5a* and *Gcm2* Fig. 7h), which are epithelial cells maintaining osmotic homeostasis [49]. The ability to pinpoint these cell fates in the context of the entire dataset echoes the key strength of combining the Atlas View with the specificity of random walk memory.

### Systematic assessments of StaVia’s visualization

**Fig. 8:**
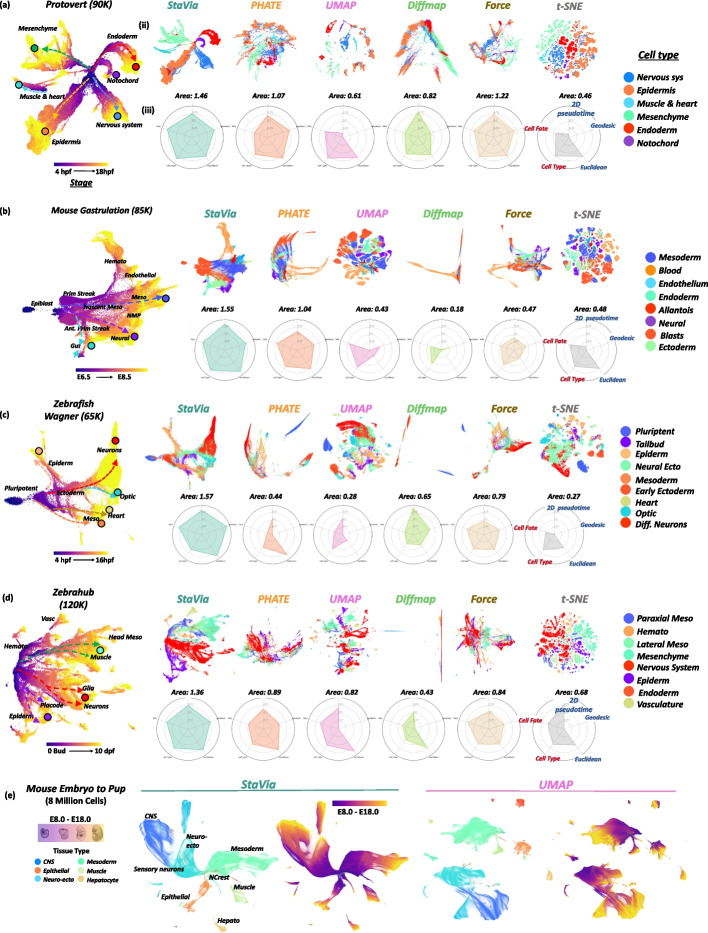
Comparison of visualization methods (**a**–**d**). (i) StaVia embedding colored by known experimental time, (ii) comparison of sc-embeddings generated by different methods colored by tissue type, and (iii) radar plot scoring for each criterion. Read clockwise, we have blue metrics (measuring sequential integrity) quantified by the correlation of the known time-series labels to the geodesic and euclidean distances from the root to other nodes in the embedded space, and also, the 2D pseudotime uses the embedding as the input to StaVia rather than the original features/principal components (PCs) which would usually be used to compute the pseudotime for TI purposes. The second set of red metrics (separation of cell types/lineages) are measured by the cell-type F1 score when clustering the 2D input using the same number of clusters for all methods and cell fate—measures how many fates are correctly detected by StaVia on the sc-embedding input rather than a higher dimensional input. **e** 8 million cells of mouse gastrula to pup (E8.0 to E18.0) [9] by StaVia and UMAP colored by major tissue type and developmental stage. PHATE, t-SNE, and force-directed layouts were attempted on this mega atlas but failed even after 24–48 h of runtime and over 30 cores of parallel processing

We systematically assessed the cartographic visualization performance of StaVia on six different single-cell transcriptomic datasets (Fig. 8a-e, Additional file 1: Figs. S12-S17). One of these is the 8-million-cell mouse gastrula to pup atlas [9] (Fig. 8e) which was only computationally accessible to StaVia and UMAP—with StaVia being able to capture the developmental relationships in a more unified manner. We also benchmarked StaVia with commonly used single-cell visualization methods: UMAP, Phate, diffusion maps, principal component analysis (PCA), force-directed layout (ForceAtlas2 [50]), and t-SNE [15]. To facilitate comparison with other methods, we use the single-cell embedding generated by StaVia (prior to the edge integration step that creates the Atlas View), together with a set of five metrics that were adapted to account for the suitability of an embedding towards TI visualization. These metrics assess the ability to (1) convey progression and (2) separate lineages/distinguish cell types (Fig. 8, Additional File 1: Fig. S13a-b and “Methods”).

We demonstrated that StaVia’s single-cell embedding can consistently portray intuitive trajectory patterns, in accordance with the experimental time points (Fig. 8a-d). StaVia also outperforms other competitive methods for faithful and robust TI visualization as evidenced by the five metrics (see the radar plot comparison in Fig. 8a-d). While UMAP [14] and Phate [16] are competitive methods for single-cell data visualization and have their respective strengths, we observed that Phate underperforms in being able to visually separate distinct cell types, although it significantly improves upon using selected diffusion components. Compared to Phate, UMAP scores well in delineating cell types, however suffers when it comes to visualizing connectivity between progenitor and progressively specialized cells. (See Additional file 1: Figs. S12-S13 for all 5 datasets colored by stage and major tissue type across benchmarked visualizations). StaVia’s strength is that it can simultaneously capture developmental chronology and continuity whilst maintaining visual separation of distinct cell types. We also note that the superior TI visualization in StaVia does not compromise the computation speed, compared to other methods (see Fig. S12b for comparison of runtimes).

We also examined the impact of individual steps in StaVia towards creating a TI-compatible visualization (Additional File 1: Fig. S14 for detailed analysis of removing each step in the algorithm in turn and Additional File 1: Figs. S15-S16 for all 5 datasets colored by stage and tissue type). Removing sequential augmentation of the single-cell KNN graph and skipping the TI cluster-graph-based initialization cause a significant drop in the ability to visualize progression. Keeping sequential augmentation but skipping the StaVia cluster graph initialization, which distills the underlying trajectory, also has a dramatic effect as quantified by the lower scores related to capturing the temporal progression as well as the visual outputs which appear more disjoint.

### Spatio-temporal cartography in StaVia captures relationships between cells across space and time

Spatial omics have expanded our understanding of tissue architecture by mapping cells in their native environments, considering both their physical locations and gene expression profiles. Yet, it remains challenging to truly integrate this spatial information with gene expression profiles, resulting in analyses remaining purely in the transcriptomic domain with resulting cluster annotations and observations subsequently merely being visually projected back onto the spatial tissue locations. This makes integrative spatial and gene expression analysis non-trivial as it is now known that cell clusters or subtypes can exhibit stark contrasts in their distribution across a tissue slice, depending on their microenvironmental neighborhood, be it highly localized or dispersed.

While our examples have until now focused on temporally varying processes, we show that StaVia can also be used to investigate spatial datasets to understand cellular landscapes based on a combination of their expression levels as well as characteristics of their spatial “habitats”. As a proof of concept, we use the pre-optic mouse hypothalamus dataset based on MERFISH (multiplexed error-robust fluorescence in situ hybridization) [17] and a spatiotemporal Stereo-Seq zebrafish gastrulation atlas ZESTA [18] to show that incorporating these spatial differences, in conjunction with gene expression, when clustering and capturing the connectivity landscape elucidates differences in cell type and function. Here, StaVia’s graph construction leverages a recent concept [51] to recalibrate gene expression by considering a cell’s environment. Furthermore, StaVia also augments the gene-expression-based KNN graph with spatial neighbors when establishing cluster connectivity (see “Methods”). Hence, the StaVia graph unifies gene expression with the spatial reality of the tissue.

**Fig. 9:**
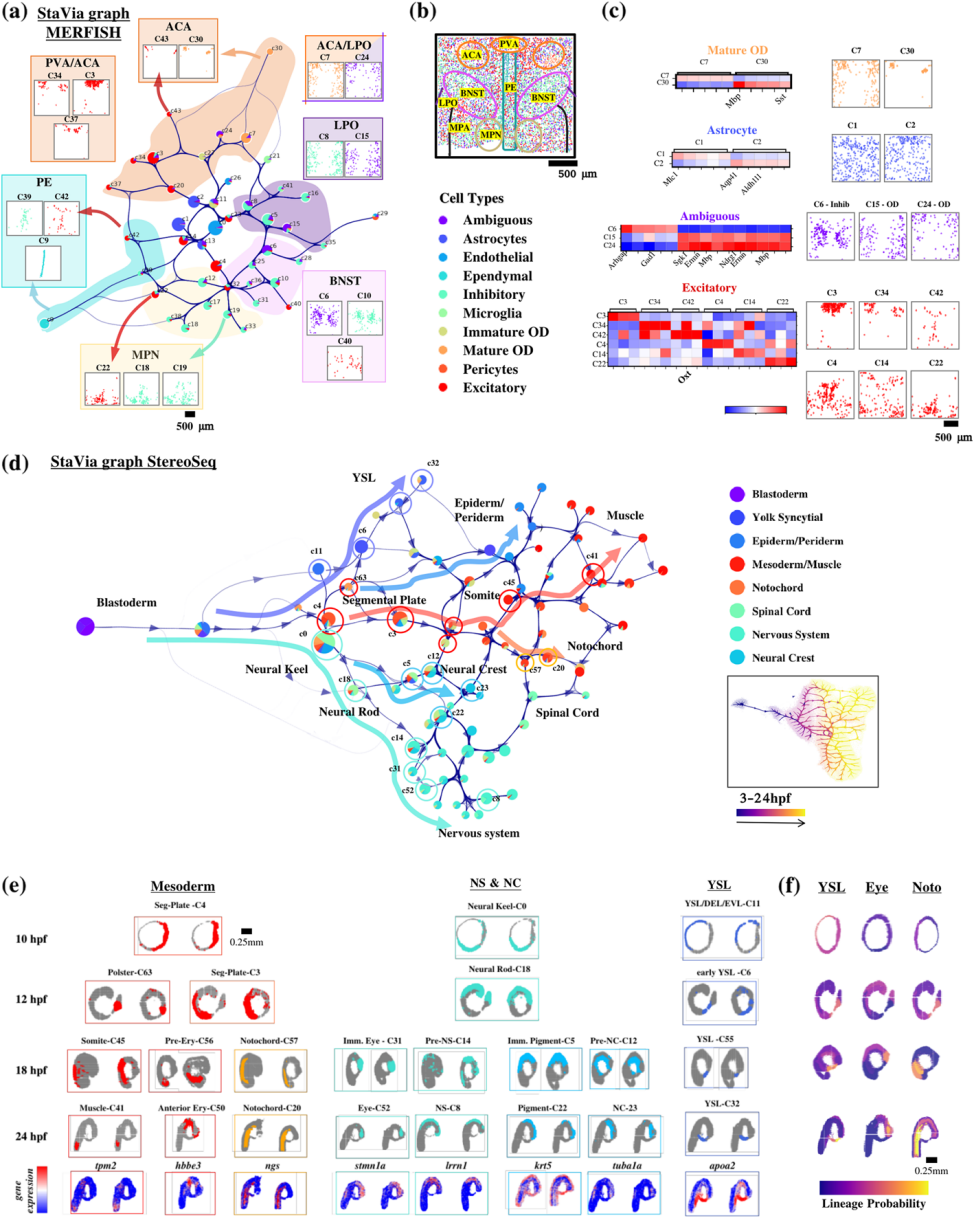
Spatiotemporally aware StaVia cartography. **a** StaVia cluster graph of mouse hypothalamus preoptic region at bregma −0.289 mm. Clusters located in similar zones share spatial “habitats”. Clusters are colored by cell type composition; all subplots share a legend for cell type coloration. Scatter plots placed near zones in the graph show the placement of cells in a cluster according to their spatial location on the tissue slice. **b** MERFISH tissue slice at −0.289 mm colored by the cell type annotations as per [17], BNST bed nucleus of the stria terminalis, MPN medial preoptic nucleus, PVA paraventricular thalamic nucleus, ACA anterior commissure, PE periventricular hypothalamic nucleus, LPO lateral preoptic area, MPA medial preoptic area. **c** The top DEGs show clear differences in corresponding subpopulations. **d** StaVia cluster graph of the trajectory, colored by major cell type. Cell and cluster annotations are made of StaVia’s clusters using the DEGs and markers compiled by Liu et al. for each cell type (**e**) progression of key cell fates for 10 hpf onwards (since 3 to 5 hpf are a common origin for all cell fates) based on cluster connectivity in the StaVia graph. Colored regions in (sub-fig **e**) correspond to labeled clusters in the cluster graph (sub-fig **d**). Lowest row shows the normalized gene expression for each cell fate by a known marker gene (see Additional file 1: Table S3d for literature references of cell type markers). **f** lineage probabilities towards three cell fates colored onto the tissue slices to show the propensity of cells on the tissue in space-time of developing towards a particular fate (see Additional file 1: Fig. S20c for Palantir and CellRank lineage probabilities

In the hypothalamus dataset, this approach yields several key results that distinguish it from the PAGA graph (Additional file 1: Fig. S19a) as well as earlier versions of VIA which did not leverage the spatial information: (1) the StaVia graph automatically generates and arranges clusters not only purely based on their expression-based cell type but also on their general tendency to occupy particular regions of the tissue section. While clusters themselves remain pure in terms of major cell classification, their cluster-level neighbors are often from a shared tissue “habitat”. The automated zoning of the tissue into neighborhoods of groups of clusters by the spatially aware cluster graph facilitates hypothesis generation and identification of potentially interesting sites or niches in a tissue where different cell types are colocalized and potentially interact to yield location-specific functions. In the StaVia cluster graph (Fig. 9a), we use the nomenclature of tissue sub-regions [52] (Fig. 9b) to roughly guide the reader regarding the identified StaVia zones. Cells found in the lower section of the tissue slice are generally placed lower in the StaVia graph, whilst those towards the ACA and PVA are found at the top of the StaVia graph. (2) StaVia identifies sub-types that are missed when omitting spatial information. The excitatory neurons are separated into multiple subtypes that are located within their respective zones on the cluster graph and express distinct DEGs (Fig. 9c). For instance, the *oxytocin*-positive excitatory cluster C42 is placed near the ependymal cluster C9. *Oxt* neurons are known to be found near ependymal cells [53], this linkage is not predicted when spatial information is left out of the computational analysis; both the oligodendrocyte and astrocyte population comprise two subtypes that present different spatial and DEG characteristics, and notably, the sub-population originally annotated as “ambiguous” seems to actually comprise of both inhibitory neurons (cluster C6 expressing *Gad1 I* [54]) and oligodendrocytes (C24 and C15 clusters, expressing *Mbp* and *Ermin* [55]). The PAGA (Additional file 1: Fig. S19a) graph is an example of a graph that does not relay very much spatial information, given the lack of a framework to incorporate this, and has a more limited set of sub-populations even when graph and clustering parameters are adjusted to increase the resolution, thus failing to achieve the results outlined above by StaVia.

We next use ZESTA [18], an STOmics (spatiotemporal multi-omics) atlas of Zebrafish gastrulation from 5 to 24 hpf of 150,000 spots approaching single cell size at a resolution of 10 × 10 × 15 μm. ZESTA exemplifies the emerging trend of STOmics atlases on which StaVia can demonstrate its unique capability of integrating both spatial tissue coordinates and real-time information in its trajectory analysis (Fig. 9d–f). StaVia arrives at a biologically sound representation of the chronology of germ layer differentiation in the first 24 h of Zebrafish development purely using the Stereo-seq data, without using supplementary scRNA-seq data for the clustering or TI calculations.

This is in contrast to current analytical approaches for STOmics data [18] where the inferred trajectory is computed on a complementary scRNA-seq dataset, and the results are subsequently projected onto the spatial data for visualization. We ran StaVia exclusively on the StereoSeq STOmics data and showed that the specification of germ layers into various cell types is captured correctly. The StaVia graph (Fig. 9d) shows the progressive commitment of pluripotent cell types towards different cell fates. Edges from early cells in each of the major tissue types are correctly connected to cells of the same tissue type of later stages (this is not the case in the PAGA graph Additional file 1: Fig. S20b where cells/clusters are predominantly connected by stage and not cell type). The clusters identified along the StaVia cluster graph as contributing to a lineage pathway are circled on the cluster graph and also colored directly on the tissue slices (Fig. 9e). The probabilities of cells moving towards a particular fate are also shown at each time point (Fig. 9f). These two subfigures thus highlight the areas on each slice at different time points that are predicted by StaVia to contribute towards the cell fate under consideration, showing progressive commitment of cells as it occurs on the tissue.

When running StaVia without the spatial information, but using only the temporal information, we see two main shortcomings (Additional file 1: Fig. S20): (1) earlier time points are not as well divided into different constituent cell types, which limits our ability to detect cell fate specification and cues at earlier stages and (2) certain sub-types are only delineated in the presence of spatial information, e.g., spatial information helps identify different clusters for the anterior and ventral spinal cord, which are distinct both in location and gene expression.

Comparison of StaVia with the PAGA graph (Additional file 1: Fig. S19b), as well as CellRank and Palantir (in terms of the inferred fate probabilities) (Additional file 1: Fig. S19c), further highlights StaVia’s strength in identifying sequential steps and intermediate stages. The PAGA graph does not accurately position the early time points (3 and 5 hpf) in relation to the other clusters. Moreover, because there is limited connectivity between cells of earlier and later cells from the same tissue type, there is scant information on how and which cells progress towards tissue-specialized fates. CellRank and Palantir were unable to detect any intermediate states, as evident in Additional file 1: Fig. S19c for three different cell fates. Even when the resolution of the method was increased (which incurred a high runtime cost in CellRank), it only led to an increase in the number of predicted final states (several of these were not from 24 hpf despite using the same root cell information as StaVia), but did not result in the identification of the relevant transition populations and differentiation pathway between 3 and 24 hpf. By contrast, StaVia manages to identify cells from each developmental time that contribute to the final cell fate at 24 hpf (Fig. 9e-f), demonstrating the unique capabilities of StaVia in trajectory analysis.

## Discussion

A salient feature of StaVia is the implementation of the second-order random walks with memory, vital to delineating the intricate end-to-end pathways of multiple lineages in the entire differentiation process. We demonstrated that without higher-order random walks with memory (by comparison to CellRank, Palantir, and by removing memory), lineage pathways run into two main issues: deviating into unrelated intermediate populations which entangles pathways and hinders lineage-specific insights or becoming so myopic in search paths that transition states are overlooked. The usefulness of StaVia’s random walks with memory to overcome these challenges without resorting to manual subsetting of stages/populations that would otherwise rob the atlas of its unique scale and perspective was demonstrated on the mouse gastrulation dataset. StaVia identified sequential transitions in hematopoiesis with new insights into hemogenic endothelial differentiation, NMP bipotency, and dual-source gut formation that are not detected by other methods unless manually curated and subsetted.

StaVia also facilitated the collective analysis of all cells in the recent Zebrahub atlas for the first time. The use of memory together with automated integration of time series information revealed insights into the bilayered epiderm formation, placode development, and the differentiation of glial cells during neurulation. Moreover, StaVia’s runtime for retrieving lineage pathways is competitive, requiring a few minutes in comparison to CellRank which needed more than 30 min on the same dataset using the same hardware. Efficient processing and runtimes aid in the discovery process by allowing the analysis to be probed across parameters on a collective atlas level without requiring access to immense computational resources.

Current state-of-the-art methods (e.g., the scVelo-directed PAGA graphs and the RNA-velocity stream plots) quickly become congested in terms of edges or streamlines and struggle to convey biological transitions. For effective visualization of complex trajectories at an atlas scale, it is imperative to establish a linkage between the overall network structure and the fine-grained transcriptomic signature [56]. StaVia’s Atlas View does this with its high edge resolution and TI-based spatial layout of cells, providing perspective on biological chronology while preserving spatial proximity of similar cells, being uniquely able to visualize development in the 8-million-cell gastrulation atlas.

As shown on the spatial-temporal Zebrafish gastrulation data (ZESTA) and the preoptic MERFISH dataset, StaVia’s framework can integrate spatial information of cells on tissues, offering perhaps the first cartographic approach to spatial transcriptomics data that conveys both location and gene-expression based similarities of cell types. This revealed several sub populations and intermediate transition states that eluded detection based purely on gene expression and also showed intra-cluster relationships between cell types based on physical location.

## Conclusion

StaVia presents an advanced TI method integrated with a new visualization approach, tailored for cell atlases that encapsulate a high degree of complexity, be it diverse lineage representation, longitudinal temporal span, non-linear spatial layout, or sheer sample size. StaVia shows that biologically faithful TI can be performed on spatial omics data without requiring a scRNA-seq for prediction accuracy. As we anticipate an increase in the creation of cell atlases with both temporal and spatial emphases, StaVia’s capabilities in delineating and visualizing cellular trajectories in large-scale and complex datasets could spearhead bioinformatics strategies that enable a more comprehensive understanding of cellular differentiation, lineage trajectories, and disease progression.

## Methods

### Key steps in the TI algorithm and visualization in StaVia

StaVia is built upon our earlier work of Via [13] that models the cellular process as a modified random walk, called LTRW, transversing the cluster graph computed by a data-driven community-detection algorithm [19]. This model incorporates elements of “laziness” (staying at the current state) and “teleportation” (jumping to any other state), with predefined probabilities. Pseudotime and graph directionality are then calculated based on state hitting times and refined with Markov chain Monte Carlo (MCMC) simulations. Here below are the key elements and steps relevant to the StaVia framework:

1. *Represent single-cell data by a sequentially augmented graph:* The first step is to represent the single-cell data by a single-cell KNN (scKNN) graph using the hierarchical navigable small world algorithm [19]. Subsequently, if sequential (temporal) data (e.g., data at different time points) is provided, then additional edges between cells in adjacent sequential groups are added. Edges between cells that are more than *t_threshold* can also be optionally removed. In the case of spatial data, the input gene expression is first modified to a weighted average of a cell’s own cells and that of its spatial neighbors. The construction of a scKNN is done in this new gene expression space (following PCA) and then augmented by spatially adjacent neighbors added to the scKNN based on spatial proximity.
2. *Build the cluster graph:* Following this, a cluster graph is constructed where nodes are PARC-based clusters of single cells. These groups of nodes can also be pre-defined by the user. Similar to Via 1.0, a pseudotime based on LTRW is first computed and the edges are accordingly forward biased. In StaVia, when available, the edge directions are also determined by the scRNA velocity. The edge-weighting and direction given by scRNA velocity versus pseudotime is controlled by a user-defined parameter (set as 0.5, i.e., 50/50 weight, by default). Start states are predicted based on the absorption probability (when scRNA velocity is available) or defined by the user. Terminal states are computed similarly to Via 1.0 using node degree and connectivity properties.
3. *Compute the lineage pathways:* Pathways (from the root to the terminals) are computed using second-order LTRWs with memory conducted on a forward-biased (directed and weighted) cluster graph. These give us a collection of simulated second-order random walks that describe the probabilistic pathways. The *memory_parameter* controls the weighting multiplier used on node edges.
4. *Construct an edge-bundled cluster graph:* The cluster graph’s node layout is computed using the Fruchterman–Reingold method. Despite the relatively modest number of clusters, edge congestion quickly arises with pruning of edges being a suboptimal way of reducing clutter. We therefore visualize edges using an edge bundling technique based on kernel density estimation (KDE) which transforms the graph into a density map and then moves edges towards the local density maxima to form bundles [57, 58].
5. *Construct a single-cell embedding for Atlas View:* The underlying single-cell embedding relies on UMAP’s implementation of minimizing the fuzzy set cross-entropy between the high- and low-dimensional representation. The cell–cell neighborhood used in the cost function computation is based on the sequentially (spatio-temporally) augmented scKNN graph. The initialization of the single-cell embedding is based on the layout of the forward-biased TI cluster graph.
6. *Generate a complete Atlas View:* The single-cell 2-D embedding layout, as described above, is clustered using kmeans (set to 150–1000 clusters depending on the desired granularity). A cluster graph is formed using the augmented sc-KNN graph and the edges are bundled using KDE. These are overlaid on the TI-cluster graph initialized single-cell embedding from step 4 to create the complete Atlas View. The bundled edges greatly aid in visually sharpening the spatial density of edges and emphasizing high-traffic edge patterns.

#### Second-order LTRW in StaVia

The concept of second-order random walks has been used previously to define search neighborhoods in feature representations of networks which can subsequently be used for classification tasks [59–61]. We extend this idea to TI computation in StaVia. Here, we use a fast implementation of the node2vec algorithm [61] to compute second-order walks on the directed cluster graph (used for lineage probability predictions and pseudotime).

The cluster graph constructed in StaVia is defined as a weighted connected graph G (V, E, W) with a vertex set *V* of *n* vertices (or nodes), i.e., $V=\{{v}_{1},\dots ,{v}_{n}\}$ and an edge set *E*, i.e., a set of ordered pairs of distinct nodes. *W* is an $nxn$ weight matrix that describes a set of edge weights between nodes *i* and *j*, ${w}_{ij}\ge 0$ are assigned to the edges $({v}_{i}, {v}_{j})$.

Assume the walker is currently on node ${v}_{cur}$ and has neighborhood ${N}_{v}$ with three neighbors $({v}_{m}, {v}_{n},{v}_{o})$ (Supplementary Fig. 18a). In the first-order case, the transition probability is given by

1 $$p({v}_{o},{v}_{\text{cur}}) = \frac{w\left({v}_{\text{cur},}{v}_{o}\right)}{{\sum }_{u ^\prime \in { N}_{v} }w({v}_{\text{cur},}u ^\prime)}$$

where the probability is only conditioned on the current state. However, in the second-order random walk, we adapt for StaVia’s lineage probability computations (Additional File 1: Fig. S18b), a bias factor *alpha*$\boldsymbol{\alpha }\le 1$ is applied to reweight edges depending on the previous state such that neighbors of the current node that are not neighbors of the previous node are considered “out-edges”. A node that is a mutual neighbor of the current and previous nodes is an inward edge, with the return-edge being the case when the next node returns to the previous one. When $\boldsymbol{\alpha }=1$, this system reverts to the first-order case. The original node2vec algorithm applies an additional biasing parameter for the return edge to discourage getting stuck in a loop that returns to the previous state. However, in our case, since we have a forward-biased weighted graph that already suppresses reverse behavior against the pseudotime, this additional biasing is not required. The transition probability in the second-order case is now given by

2 $$p({v}_{o}|{v}_{\text{curr}},{v}_{\text{prev}})= \frac{\alpha \left({v}_{\text{cur}},{v}_{o}\right)w({v}_{\text{cur}},{v}_{\text{o}})}{\sum_{u ^\prime \epsilon {N}_{v}}\alpha \left({v}_{\text{cur}},{u} ^\prime \right)w({u} ^\prime,{v}_{\text{cur}})}$$

which generalizes to

3 $${p(v}_{\text{next}}|{v}_{\text{cur}}, {v}_{\text{prev}}) = \frac{\alpha ({v}_{\text{cur}},{v}_{\text{next}}) w({v}_{\text{cur}},{v}_{\text{next}})}{{\sum }_{u ^\prime \in { N}_{v} }\alpha ({v}_{\text{cur}},u ^\prime )w(u ^\prime,{v}_{\text{cur}})}\, for\, ({v}_{\text{cur}},{v}_{\text{next}}) \in {N}_{v} ,\text{ else} \,0$$

and it has bias factor $\alpha$, given by

4 $$\alpha ({v}_{\text{next}},{v}_{\text{prev}}) = \frac{1}{\text{Memory}}, \,\text{for}\, ({v}_{\text{next}},{v}_{\text{prev}}) \notin E \,\text{else}, \,1 \,\text{for} \,({v}_{\text{next}},{v}_{\text{prev}}) \in E$$

Stability of the TI when changing the memory parameter and a short guide to selecting a suitable range is described below and in Additional file 1: Fig. S5.

#### Kernel density estimation (KDE)-based edge bundling

The graph bundling for the cluster graphs and Atlas View uses a kernel density estimation-based method [57, 58, 62]. Combining this with StaVia’s single-cell atlas embeddings aids in visually summarizing the edge density and highlighting pathways based on their traffic (cell–cell interactions). Briefly, the KDE edge bundling is an iterative algorithm that repeats the following set of steps on a graph drawing: first convolve the edges with a kernel to construct a density map. The density is a measure of the number of edges at that particular location in space. Next, compute the gradient of the density map $\Delta \rho$ and advect points $x\in G$ in the direction of $\Delta \rho$ and do Laplacian filtering to smooth the edges. Repeat these steps, reducing the kernel bandwidth on each iteration. The effect will be to sharpen the density such that straight-lined unbundled edges will be drawn as tightly bundled curves.

Given a graph drawing $G\subset {R}^{2}$ with edges $E=\{{{e}_{i}\subset R}^{2}\}$ where $x\in {G}^{2}$, the density map is given by

5 $$\rho (x)= \sum \nolimits_{i=1}^{N}{\int }_{y\in {e}_{i}}K\left(\frac{x-y}{h}\right)$$

The kernel used here is the Epanechnikov kernel

6 $$K\left({\varvec{x}}\right)=1-{||{\varvec{x}}||}^{2}$$

The bandwidth is reduced by a factor $\lambda$ at each iteration, such that on the ${n}^{\text{th}}$ step, it will be ${h}_{n}={\lambda }^{n}{h}_{\text{max}}$.

### Robustness analysis of memory parameter

We have investigated the utility of incorporating random walks with memory in the context of both mouse and zebrafish gastrulation, whereby gradually increasing the level of memory in the random walk improves end-to-end pathway mapping and the specificity of gene trends associated with the emergence of lineages. However, at very high levels of memory (e.g., memory = 100), some pathways can be too restrictive. We show that it is possible to narrow down or gauge an optimal range for the memory parameter by correlating the known experimental times to the inferred pseudotime (computed at different memory values). For Zebrahub and mouse gastrulation, the correlation increases with memory, remaining elevated for an interval before decreasing at higher levels of memory Additional file 1: Fig. S5b-c. To show that the change incurred by memory is gradual and behaves in a stable manner, we also present a correlation analysis in Additional file 1: Fig. S5a of lineage probabilities for cell fates in the Zebrhub dataset at memory values {1,5,10,50,100}, where a value of 1 signifies no memory. These show that for a wide range of values, the analysis is highly correlated.

#### Metrics for quantitative analysis of visualizations

The metrics fall into either structural or cell-type measures. Structural metrics to assess how well a method visualization sequential information and progression include standard Pearson correlation ${\varvec{r}}({\varvec{x}},{\varvec{y}})$ in the following contexts:

7 $$r=\frac{\sum \left({x}_{i}-\overline{x }\right)\left({y}_{i}-\overline{y }\right)}{\sum {\left({x}_{i}-\overline{x }\right)}^{2}{\left({y}_{i}-\overline{y }\right)}^{2}}$$

- *2D-pseudotime:* Time series label and StaVia-pseudotime, where StaVia-pseudotime is the inferred pseudotime by StaVia when the 2D embedding is given as the input to StaVia.
- *Geodesic:* Time series label and geodesic distance on the embedding from root to cells. The geodesic distance ${d}^{\text{geo}}(u,v)$ between two nodes $u \,\text{and}\, v$ on a weighted graph is the minimum sum of weights across all the paths connecting *u* and *v*.
- *Euclidean:* Time series labels and Euclidean distance on the embedding from root to cells. The Euclidean distance ${d}^{\text{Euc}}(u,v)$ between two cells $u \,\text{and}\, v$ whose coordinates are given by the 2D embedding,
  8 $${d}^{\text{Euc}}\left(u,v\right)= \sqrt{\sum\nolimits_{u ^\prime\in {N}_{v}}{({u}_{i}-{v}_{i})}^{2}}$$

On the other hand, the metrics that assess how well a visualization method captures lineage divergence and respects cell type separation include:

- *Cell fate:* Cell fate detection when running StaVia on the embedding. StaVia’s automatic cell fate detection is applied to all embeddings (the same root state is provided in all cases). We compute the F1 score of detected cell fates with reference to expected cell fates (corresponding to the later stages of the dataset)
- *Cell type:* Use k-means clustering on the 2D embedding at a fixed number of clusters for all embeddings. For each dataset, the number of clusters is set to five clusters more than the number of given coarse-level annotations resulting in typically around 15–20 clusters. Calculate the F1-score using the scoring method applied in Stassen 2020 which assigns each cluster a majority reference population, aggregates the clusters assigned to said reference population, and calculates the one-vs-all F1 score for each reference population. The mean score across reference populations is reported which avoids the issue of larger cell populations dominating the score. This approach prevents punishing a method for splitting a cell type into multiple clusters, which may well be the case since the annotations are coarse and would not necessarily capture subtypes. Since all methods are given the same number of k-clusters, it is still a fair comparison.

#### Spatially aware cartography construction in StaVia

Our strategy has two key elements which play a role in embedding spatial information into the cartography. The first element uses a concept (Singhal 2024) that recomputes the gene expression as a weighted average of a cell’s own expression plus that of its spatial neighbors. The second component of the spatial integration is to augment the single-cell gene-expression-based KNN graph with neighbors found in the spatial domain before computing inter-cluster connectivity. The average spatial location of clusters is subsequently used to initialize the layout of the StaVia graph.

#### Pre-processing datasets

Mouse gastrulation [6]: scvelo’s filter_and_normalize function is used on the raw spliced and unspliced genes. Only genes (both in spliced and unspliced counts) that are expressed in 20 or more cells are retained, resulting in a matrix with 10,766 genes across 89,267 cells. Each cell is normalized by the counts over all its genes. The last step is to log normalize the counts before PCA. The velocities are computed using scvelo’s stochastic mode as the dynamic mode is prohibitively slow for large datasets. PCA done on the full filtered gene set.

Zebrahub [7]: Single-cell sequencing of 120,437 cells from zebrafish embryonic development across 10 time points from 10 hpf to 10 dpf. Cells expressing fewer than 200 genes and genes expressed by fewer than 5 cells were removed. Each cell is normalized by the counts over all its genes, followed by log normalization. The top 5000 highly variable genes are used for PCA. Due to the computational demands of scVelo for large datasets, we use the velocity matrix publicly available by Zebrahub which was computed using scVelo. Cell annotations were combined from the datasets for individual time points.

Ascidian Protovert [63]: Early phases of embryogenesis of ascidian protovertebrate with sequentially staged Ciona embryos, from gastrulation at the 110-cell stage to neurula and larval stage. Individual h5 matrices for each time point are concatenated into an Anndata object containing 90,579 cells. Standard gene filtering (min_cells = 5, min_counts = 10) is done using scanpy and each cell is normalized by the counts over its genes followed by log normalization. Top 2000 highly variable genes are used towards PCA. Root cell is user defined based on timestamps as stage 1 epidermis cell.

Zebrafish [64]: 63,273 cells across 7 time points from the first 24 h of zebrafish embryo Same preprocessing as Ascidian Protovert data. Top 2000 highly variable genes are used towards PCA.

Mouse neuron (La Manno 2021): 292,495 cells from embryonic mouse brain from stages E8–E18. Same preprocessing as Ascidian protovert. scvelo is used to compute the scRNA velocity. Top 2000 highly variable genes are used towards PCA.

MERFISH [17]: MERFISH of 12 slices of mouse preoptic region 1.8 mm × 1.8 mm × 0.1 µm thick of 160 genes chosen based on scRNA-seq marker gene panel and known functional gene panel. Figure 9 is 6500 cells from a naive female mouse slice at −0.289 mm bregma. Analysis of slices from other bregma shows similar spatially aware StaVia graphs and clustering tendencies.

ZESTA [18]: Stereo-seq dataset profiling 91 zebrafish embryo sections covering six time points during the first 24 h of development. Filtered data of individual time points (thresholded according to > 350 genes for the 15-µm bin) were available on the Zesta portal. These time points are merged, resulting in a total of 152,977 spots at a resolution of 10 × 10 × 15 μm^3^ (close to cellular size) with spatial coordinates for use in the StaVia analysis. The cell annotations provided by C.Liu are used as a starting point, with some refinements made based on finer clustering resolution in StaVia and known marker gene expression.

Mouse pup [9]: Three-level single-cell transcriptional profiling by combinatorial indexing (sci-RNA-seq3) profiling over 8 million nuclei from 83 staged embryos spanning late gastrulation (E8.0) to the end of gastrulation at E18.75, with 2-h temporal resolution during somitogenesis and 6-h resolution through to birth.

*TI parameters* for StaVia, PAGA, CellRank, and *visualization parameters* for all methods are provided in Additional file 1: Table S1 and Table S2 with the number of KNN and PCs consistent for each method and key parameters highlighted where changed from default in order to improve results. No batch correction based on experimental times was performed for the datasets during TI analysis or visualization benchmarking. The UMAP embeddings used to present CellRank’s TI results use the UMAPs resulting from batch-corrected PCs used in the Zebrahub and Sala publications.

### Supplementary Information

Additional file 1. Additional figures, notes and tables [65–94].

Additional file 2. Review history

## Data Availability

ZESTA: raw Stereo-seq and scRNA-seq data are available at CNGB Nucleotide Sequence Archive: CNP0002220. Processed data for StaVia analysis Figshare https://doi.org/10.6084/m9.figshare.25249105 (2024). Merfish preoptic: MERFISH data are available on Dryad (doi:https://doi.org/10.5061/dryad.8t8s248). Processed data for StaVia analysis Figshare https://doi.org/10.6084/m9.figshare.26028571 (2024). Mouse gastrulation: raw data is available on ArrayExpress with accession: Atlas: E-MTAB-6967. Processed data for StaVia is available on Figshare https://doi.org/10.6084/m9.figshare.26028550 (2024). Data for Ascidian Protovert is available at NCBI GEO with accession GSE131155. Processed data for use with StaVia is available on Figshare https://doi.org/10.6084/m9.figshare.26054131 (2024). Zebrafish Wagner: raw data is available at NCBI GEO with accession GSE112294. Processed data is available on Figshare https://doi.org/10.6084/m9.figshare.26054173 (2024). Data for mouse pup can be downloaded in raw and processed forms from the NCBI Gene Expression Omnibus (GEO) under accession numbers GSE186069 and GSE228590. Zebrahub data: raw data for Zebrahub is available at (https://zebrahub.ds.czbiohub.org/). Processed data for StaVia analysis Figshare https://doi.org/10.6084/m9.figshare.26028325 (2024). Processed Anndata h5ad files used in this paper for the datasets above are also available at https://github.com/ShobiStassen/VIA.

## References

[1] Quake SR. A decade of molecular cell atlases. Trends Genet. 2022;38(8):805–10. 10.1016/j.tig.2022.01.004. Epub 2022 Jan 31. PMID: 35105475. 35105475 10.1016/j.tig.2022.01.004

[2] The Tabula Sapiens Consortium*. The Tabula Sapiens: a multiple-organ, single-cell transcriptomic atlas of humans. Science. 2022;376:eabl4896. 10.1126/science.abl4896. 35549404 10.1126/science.abl4896 PMC9812260

[3] Calderon D, Blecher-Gonen R, Huang X, Secchia S, Kentro J, Daza RM, Martin B, Dulja A, Schaub C, Trapnell C, Larschan E, O’Connor-Giles KM, Furlong EEM, Shendure J. The continuum of Drosophila embryonic development at single-cell resolution. Science. 2022;377(6606):eabn5800. 10.1126/science.abn5800. Epub 2022 Aug 5. PMID: 35926038; PMCID: PMC9371440. 35926038 10.1126/science.abn5800 PMC9371440

[4] Qiu C, Cao J, Martin BK, et al. Systematic reconstruction of cellular trajectories across mouse embryogenesis. Nat Genet. 2022;54:328–41. 10.1038/s41588-022-01018-x. 35288709 10.1038/s41588-022-01018-x PMC8920898

[5] Packer JS, Zhu Q, Huynh C, Sivaramakrishnan P, Preston E, Dueck H, Stefanik D, Tan K, Trapnell C, Kim J, Waterston RH, Murray JI. A lineage-resolved molecular atlas of C. elegans embryogenesis at single-cell resolution. Science. 2019;365(6459):eaax1971. 10.1126/science.aax1971. Epub 2019 Sep 5. PMID: 31488706; PMCID: PMC7428862. 31488706 10.1126/science.aax1971 PMC7428862

[6] Pijuan-Sala B, Griffiths JA, Guibentif C, et al. A single-cell molecular map of mouse gastrulation and early organogenesis. Nature. 2019;566:490–5. 10.1038/s41586-019-0933-9. 30787436 10.1038/s41586-019-0933-9 PMC6522369

[7] Lange, Merlin, et al. Zebrahub – multimodal zebrafish developmental atlas reveals the state transition dynamics of late vertebrate pluripotent axial progenitors preprint at bioRxiv 2023.03.06.531398. 2023. 10.1101/2023.03.06.531398. 10.1016/j.cell.2024.09.047 39454574

[8] Sikkema L, Ramírez-Suástegui C, Strobl DC, et al. An integrated cell atlas of the lung in health and disease. Nat Med. 2023;29:1563–77. 10.1038/s41591-023-02327-2. 37291214 10.1038/s41591-023-02327-2 PMC10287567

[9] Qiu C, Martin BK, Welsh IC, et al. A single-cell transcriptional timelapse of mouse embryonic development, from gastrula to pup. bioRxiv [Preprint]. 2023:2023.04.05.535726. 10.1101/2023.04.05.535726. PMID: 37066300; PMCID: PMC10104014.

[10] Setty M, Kiseliovas V, Levine J, et al. Characterization of cell fate probabilities in single-cell data with Palantir. Nat Biotechnol. 2019;37:451–60. 10.1038/s41587-019-00. 30899105 10.1038/s41587-019-00 PMC7549125

[11] Pandey K, Zafar H. Inference of cell state transitions and cell fate plasticity from single-cell with MARGARET. Nucleic Acids Res. 2022;50(15):e86. 10.1093/nar/gkac412. PMID: 35639499; PMCID: PMC9410915. 35639499 10.1093/nar/gkac412 PMC9410915

[12] Lange M, Bergen V, Klein M, et al. CellRank for directed single-cell fate mapping. Nat Methods. 2022;19:159–70. 10.1038/s41592-021-01346-6. 35027767 10.1038/s41592-021-01346-6 PMC8828480

[13] Stassen SV, Yip GGK, Wong KKY, et al. Generalized and scalable trajectory inference in single-cell omics data with VIA. Nat Commun. 2021;12:5528. 10.1038/s41467-021-25773-3. 34545085 10.1038/s41467-021-25773-3 PMC8452770

[14] McInnes L, Healy J, Saul N, Großberger L. UMAP: uniform manifold approximation and projection. J Open Source Softw. 2018;3:861. 10.21105/joss.00861

[15] van der Maaten L, Hinton G. Visualizing data using t-SNE. J Mach Learn Res. 2008;9:2579–605.

[16] Moon KR, van Dijk D, Wang Z, et al. Visualizing structure and transitions in high-dimensional biological data. Nat Biotechnol. 2019;37:1482–92. 10.1038/s41587-019-0336-3. 31796933 10.1038/s41587-019-0336-3 PMC7073148

[17] Moffitt JR, Bambah-Mukku D, Eichhorn SW, Vaughn E, Shekhar K, Perez JD, Rubinstein ND, Hao J, Regev A, Dulac C, Zhuang X. Molecular, spatial, and functional single-cell profiling of the hypothalamic preoptic region. Science. 2018;362(6416):eaau5324. 10.1126/science.aau5324. Epub 2018 Nov 1. PMID: 30385464; PMCID: PMC6482113. 30385464 10.1126/science.aau5324 PMC6482113

[18] Liu C, Li R, Li Y, Lin X, Zhao K, Liu Q, Wang S, Yang X, Shi X, Ma Y, Pei C, Wang H, Bao W, Hui J, Yang T, Xu Z, Lai T, Berberoglu MA, Sahu SK, Esteban MA, Ma K, Fan G, Li Y, Liu S, Chen A, Xu X, Dong Z, Liu L. Spatiotemporal mapping of gene expression landscapes and developmental trajectories during zebrafish embryogenesis. Dev Cell. 2022;57(10):1284-1298.e5. 10.1016/j.devcel.2022.04.009. Epub 2022 May 4. PMID: 35512701. 35512701 10.1016/j.devcel.2022.04.009

[19] Stassen SV, Siu DMD, Lee KCM, Ho JWK, So HKH, Tsia KK. PARC: ultrafast and accurate clustering of phenotypic data of millions of single cells. Bioinformatics. 2020;36(9):2778–86. 10.1093/bioinformatics/btaa042. 31971583 10.1093/bioinformatics/btaa042 PMC7203756

[20] Wolf FA, Hamey FK, Plass M, et al. PAGA: graph abstraction reconciles clustering with trajectory inference through a topology preserving map of single cells. Genome Biol. 2019;20:59. 10.1186/s13059-019-1663-x. 30890159 10.1186/s13059-019-1663-x PMC6425583

[21] Gao L, Tober J, Gao P, Chen C, Tan K, Speck NA. RUNX1 and the endothelial origin of blood. Exp Hematol. 2018;68:2–9. 10.1016/j.exphem.2018.10.009. 30391350 10.1016/j.exphem.2018.10.009 PMC6494457

[22] Thambyrajah R, Mazan M, Patel R, Moignard V, Stefanska M, Marinopoulou E, Li Y, Lancrin C, Clapes T, Möröy T, et al. GFI1 proteins orchestrate the emergence of haematopoietic stem cells through recruitment of LSD1. Nat Cell Biol. 2016;18:21–32. 10.1038/ncb3276. 26619147 10.1038/ncb3276

[23] Guibentif C, Griffiths JA, Imaz-Rosshandler I, Ghazanfar S, Nichols J, Wilson V, Göttgens B, Marioni JC. Diverse routes toward early somites in the mouse embryo. Dev Cell. 2021;56(1):141-153.e6. 10.1016/j.devcel.2020.11.013. Epub 2020 Dec 11. PMID: 33308481; PMCID: PMC7808755. 33308481 10.1016/j.devcel.2020.11.013 PMC7808755

[24] Nowotschin S, Setty M, Kuo YY, Liu V, Garg V, Sharma R, Simon CS, Saiz N, Gardner R, Boutet SC, Church DM, Hoodless PA, Hadjantonakis AK, Pe’er D. The emergent landscape of the mouse gut endoderm at single-cell resolution. Nature. 2019;569(7756):361–7. 10.1038/s41586-019-1127-1. Epub 2019 Apr 8. PMID: 30959515; PMCID: PMC6724221. 30959515 10.1038/s41586-019-1127-1 PMC6724221

[25] Kwon GS, Viotti M, Hadjantonakis AK. The endoderm of the mouse embryo arises by dynamic widespread intercalation of embryonic and extraembryonic lineages. Dev Cell. 2008;15(4):509–20. 10.1016/j.devcel.2008.07.017. PMID: 18854136; PMCID: PMC2677989. 18854136 10.1016/j.devcel.2008.07.017 PMC2677989

[26] Wu Y, Hirschi KK. Regulation of hemogenic endothelial cell development and function. Annu Rev Physiol. 2021;83:17–37. 10.1146/annurev-physiol-021119-034352. Epub 2020 Oct 9. PMID: 33035429; PMCID: PMC8634156. 33035429 10.1146/annurev-physiol-021119-034352 PMC8634156

[27] Bergen V, Lange M, Peidli S, et al. Generalizing RNA velocity to transient cell states through dynamical modeling. Nat Biotechnol. 2020;38:1408–14. 10.1038/s41587-020-0591-3. 32747759 10.1038/s41587-020-0591-3

[28] Haedicke J, Brown C, Naghavi MH. The brain-specific factor FEZ1 is a determinant of neuronal susceptibility to HIV-1 infection. Proc Natl Acad Sci U S A. 2009;106(33):14040–5. 10.1073/pnas.0900502106. Epub 2009 Aug 10. PMID: 19667186; PMCID: PMC2729016. 19667186 10.1073/pnas.0900502106 PMC2729016

[29] Duan D, Fu Y, Paxinos G, Watson C. Spatiotemporal expression patterns of Pax6 in the brain of embryonic, newborn, and adult mice. Brain Struct Funct. 2013;218(2):353–72. 10.1007/s00429-012-0397-2. Epub 2012 Feb 22. PMID: 22354470. 22354470 10.1007/s00429-012-0397-2

[30] Cheung M, Briscoe J. Neural crest development is regulated by the transcription factor Sox9. Development. 2003;130(23):5681–93. 10.1242/dev.00808. Epub 2003 Oct 1. PMID: 14522876. 14522876 10.1242/dev.00808

[31] Perea-Gomez A, Meilhac SM. Formation of the anterior-posterior axis in mammals. 2015. https://api.semanticscholar.org/CorpusID:80823225.

[32] Frisdal A, Trainor PA. Development and evolution of the pharyngeal apparatus. Wiley Interdiscip Rev Dev Biol. 2014;3(6):403–18. 10.1002/wdev.147. Epub 2014 Aug 29. PMID: 25176500; PMCID: PMC4199908. 25176500 10.1002/wdev.147 PMC4199908

[33] Neacsu CD, Ko YP, Tagariello A, Røkenes Karlsen K, Neiss WF, Paulsson M, Wagener R. Matrilin-1 is essential for zebrafish development by facilitating collagen II secretion. J Biol Chem. 2014;289(3):1505–18. 10.1074/jbc.M113.529933. Epub 2013 Nov 29. PMID: 24293366; PMCID: PMC3894332. 24293366 10.1074/jbc.M113.529933 PMC3894332

[34] Gilbert SF. Developmental biology. 6th ed. Sunderland: Sinauer Associates; 2000. Lateral Plate Mesoderm. Available from: https://www.ncbi.nlm.nih.gov/books/NBK9982/.

[35] Tani S, Chung UI, Ohba S, et al. Understanding paraxial mesoderm development and sclerotome specification for skeletal repair. Exp Mol Med. 2020;52:1166–77. 10.1038/s12276-020-0482-1. 32788657 10.1038/s12276-020-0482-1 PMC8080658

[36] Kahane N, Kalcheim C. From bipotent neuromesodermal progenitors to neural-mesodermal interactions during embryonic development. Int J Mol Sci. 2021;22(17):9141. 10.3390/ijms22179141. PMID: 34502050; PMCID: PMC8431582. 34502050 10.3390/ijms22179141 PMC8431582

[37] Mork L, Crump G. Zebrafish craniofacial development: a window into early patterning. Curr Top Dev Biol. 2015;115:235–69. 10.1016/bs.ctdb.2015.07.001. Epub 2015 Oct 6. PMID: 26589928; PMCID: PMC4758817. 26589928 10.1016/bs.ctdb.2015.07.001 PMC4758817

[38] Knight RD, Schilling TF. Cranial neural crest and development of the head skeleton. Adv Exp Med Biol. 2006;589:120–33. 10.1007/978-0-387-46954-6_7. PMID: 17076278. 17076278 10.1007/978-0-387-46954-6_7

[39] Chatterjee M, Li JY. Patterning and compartment formation in the diencephalon. Front Neurosci. 2012;6:66. 10.3389/fnins.2012.00066. PMID: 22593732; PMCID: PMC3349951. 22593732 10.3389/fnins.2012.00066 PMC3349951

[40] Peukert D, Weber S, Lumsden A, Scholpp S. Lhx2 and Lhx9 determine neuronal differentiation and compartition in the caudal forebrain by regulating Wnt signaling. PLoS Biol. 2011;9(12):e1001218. 10.1371/journal.pbio.1001218. Epub 2011 Dec 13. PMID: 22180728; PMCID: PMC3236734. 22180728 10.1371/journal.pbio.1001218 PMC3236734

[41] Li H, Chang YW, Mohan K, Su HW, Ricupero CL, Baridi A, Hart RP, Grumet M. Activated Notch1 maintains the phenotype of radial glial cells and promotes their adhesion to laminin by upregulating nidogen. Glia. 2008;56(6):646–58. 10.1002/glia.20643. PMID: 18286610; PMCID: PMC2712347. 18286610 10.1002/glia.20643 PMC2712347

[42] Dang L, Yoon K, Wang M, Gaiano N. Notch3 signaling promotes radial glial/progenitor character in the mammalian telencephalon. Dev Neurosci. 2006;28(1–2):58–69. 10.1159/000090753. PMID: 16508304. 16508304 10.1159/000090753

[43] Dimou L, Götz M. Glial cells as progenitors and stem cells: new roles in the healthy and diseased brain. Physiol Rev. 2014;94(3):709–37. 10.1152/physrev.00036.2013. PMID: 24987003. 24987003 10.1152/physrev.00036.2013

[44] Ohyama T, Mohamed OA, Taketo MM, Dufort D, Groves AK. Wnt signals mediate a fate decision between otic placode and epidermis. Development. 2006;133(5):865–75. 10.1242/dev.02271. Epub 2006 Feb 1. PMID: 16452098. 16452098 10.1242/dev.02271

[45] Park BY, Saint-Jeannet JP. Induction and segregation of the vertebrate cranial placodes. San Rafael: Morgan & Claypool Life Sciences; 2010. Introduction. Available from: https://www.ncbi.nlm.nih.gov/books/NBK53171/. 21452441

[46] Steiner AB, Kim T, Cabot V, Hudspeth AJ. Dynamic gene expression by putative hair-cell progenitors during regeneration in the zebrafish lateral line. Proc Natl Acad Sci U S A. 2014;111(14):E1393-401. 10.1073/pnas.1318692111. Epub 2014 Mar 27. PMID: 24706895; PMCID: PMC3986164. 24706895 10.1073/pnas.1318692111 PMC3986164

[47] Manuel R, Iglesias Gonzalez AB, Habicher J, Koning HK, Boije H. Characterization of individual projections reveal that neuromasts of the zebrafish lateral line are innervated by multiple inhibitory efferent cells. Front Neuroanat. 2021;15:666109. 10.3389/fnana.2021.666109. PMID: 34234651; PMCID: PMC8255702. 34234651 10.3389/fnana.2021.666109 PMC8255702

[48] Teixeira Rosa J, Oralová V, Larionova D, Eisenhoffer GT, Eckhard Witten P, Huysseune A. Periderm invasion contributes to epithelial formation in the teleost pharynx. Sci Rep. 2019;9(1):10082. 10.1038/s41598-019-46040-y. PMID: 31300674; PMCID: PMC6626026. 31300674 10.1038/s41598-019-46040-y PMC6626026

[49] Peloggia J, Münch D, Meneses-Giles P, Romero-Carvajal A, Lush ME, Lawson ND, McClain M, Pan YA, Piotrowski T. Adaptive cell invasion maintains lateral line organ homeostasis in response to environmental changes. Dev Cell. 2021;56(9):1296-1312.e7. 10.1016/j.devcel.2021.03.027. Epub 2021 Apr 19. PMID: 33878346; PMCID: PMC8142321. 33878346 10.1016/j.devcel.2021.03.027 PMC8142321

[50] Jacomy M, Venturini T, Heymann S, Bastian M. ForceAtlas2, a continuous graph layout algorithm for handy network visualization designed for the Gephi software. PLoS One. 2014;9(6):e98679. 24914678 10.1371/journal.pone.0098679 PMC4051631

[51] Singhal V, Chou N, Lee J, et al. BANKSY unifies cell typing and tissue domain segmentation for scalable spatial omics data analysis. Nat Genet. 2024;56:431–41. 10.1038/s41588-024-01664-3. 38413725 10.1038/s41588-024-01664-3 PMC10937399

[52] Paxinos G, Franklin KBJ. Paxinos and Franklin’s the mouse brain in stereotaxic coordinates. 5th edn. Academic; 2019. eBook ISBN: 9780128161586.

[53] Jurek B, Neumann ID. the oxytocin receptor: from intracellular signaling to behavior. Physiol Rev. 2018;98(3):1805–2190. 29897293 10.1152/physrev.00031.2017

[54] Krishnan V, Wade-Kleyn LC, Israeli RR, Pelled G. peripheral nerve injury induces changes in the activity of inhibitory interneurons as visualized in transgenic GAD1-GCaMP6s rats. Biosensors (Basel). 2022;12(6):383. 10.3390/bios12060383. PMID: 35735531; PMCID: PMC9221547. 35735531 10.3390/bios12060383 PMC9221547

[55] Brockschnieder D, Sabanay H, Riethmacher D, Peles E. Ermin, a myelinating oligodendrocyte-specific protein that regulates cell morphology. J Neurosci. 2006;26(3):757–62. 10.1523/JNEUROSCI.4317-05.2006. PMID: 16421295; PMCID: PMC6675369. 16421295 10.1523/JNEUROSCI.4317-05.2006 PMC6675369

[56] Li MM, Huang K, Zitnik M. Graph representation learning in biomedicine and healthcare. Nat Biomed Eng. 2022;6:1353–69. 10.1038/s41551-022-00942-x. 36316368 10.1038/s41551-022-00942-x PMC10699434

[57] Hurter C, Ersoy O, Telea AC. Graph bundling by kernel density estimation. In: EUROVIS 2012, Eurographics conference on visualization. Vienna; 2012. p. 865–874. 10.1111/j.1467-8659.2012.03079.x

[58] van der Zwan M, Codreanu V, Telea A. CUBu: universal real-time bundling for large graphs. IEEE Trans Vis Comput Graph. 2016;22(12):2550–63. 10.1109/TVCG.2016.2515611. 26761819 10.1109/TVCG.2016.2515611

[59] Grover A, Leskovec J. node2vec: scalable feature learning for networks. KDD. 2016;2016:855–64. 10.1145/2939672.2939754. PMID: 27853626; PMCID: PMC5108654. 27853626 10.1145/2939672.2939754 PMC5108654

[60] Liu R, Krishnan A. PecanPy: a fast, efficient and parallelized Python implementation of node2vec. Bioinformatics. 2021;37(19):3377–9. 10.1093/bioinformatics/btab202. 33760066 10.1093/bioinformatics/btab202 PMC8504639

[61] Liu R, et al. Accurately modeling biased random walks on weighted networks using node2vec+. Bioinformatics. 2023;39(1):btad047. 10.1093/bioinformatics/btad04. 36688699 10.1093/bioinformatics/btad04 PMC9891245

[62] Cottam JA, Lumsdaine A, Wang P. Abstract rendering: out-of-core rendering for information visualization. In: Proc. SPIE 9017, visualization and data analysis 2014, 90170K. 2014. 10.1117/12.2041200.

[63] Cao C, Lemaire LA, Wang W, Yoon PH, Choi YA, Parsons LR, et al. Comprehensive single-cell transcriptome lineages of a proto-vertebrate. Nature. 2019;571(7765):349–54. 10.1038/s41586-019-1385-y. 31292549 10.1038/s41586-019-1385-y PMC6978789

[64] Wagner DE, Weinreb C, Collins ZM, Briggs JA, Megason SG, Klein AM. Single-cell mapping of gene expression landscapes and lineage in the zebrafish embryo. Science. 2018;360(6392):981–7. 10.1126/science.aar4362. 29700229 10.1126/science.aar4362 PMC6083445

[65] Edri S, Hayward P, Jawaid W, Martinez Arias A. Neuro-mesodermal progenitors (NMPs): a comparative study between pluripotent stem cells and embryo-derived populations. Development. 2019;146(12):dev180190. 10.1242/dev.180190. PMID: 31152001; PMCID: PMC6602346. 31152001 10.1242/dev.180190 PMC6602346

[66] Canu G, Ruhrberg C. First blood: the endothelial origins of hematopoietic progenitors. Angiogenesis. 2021;24:199–211. 10.1007/s10456-021-09783-9. 33783643 10.1007/s10456-021-09783-9 PMC8205888

[67] Hayashi M, Pluchinotta M, Momiyama A, Tanaka Y, Nishikawa S, Kataoka H. Endothelialization and altered hematopoiesis by persistent Etv2 expression in mice. Exp Hematol. 2012;40(9):738-750.e11. 10.1016/j.exphem.2012.05.012. Epub 2012 Jun 1. PMID: 22659386. 22659386 10.1016/j.exphem.2012.05.012

[68] Shen J, et al. Single-cell transcriptome of early hematopoiesis guides arterial endothelial-enhanced functional T cell generation from human PSCs. Sci Adv. 2021;7:eabi9787. 10.1126/sciadv.abi9787. 34516916 10.1126/sciadv.abi9787 PMC8442917

[69] Cambray N, Wilson V. Two distinct sources for a population of maturing axial progenitors. Development. 2007;134(15):2829–40. 10.1242/dev.02877. Epub 2007 Jul 4. PMID: 17611225. 17611225 10.1242/dev.02877

[70] Steventon B, Mayor R, Streit A. Neural crest and placode interaction during the development of the cranial sensory system. Dev Biol. 2014;389(1):28–38. 10.1016/j.ydbio.2014.01.021. Epub 2014 Jan 31. PMID: 24491819; PMCID: PMC4439187. 24491819 10.1016/j.ydbio.2014.01.021 PMC4439187

[71] Steventon B, Martinez AA. Evo-engineering and the cellular and molecular origins of the vertebrate spinal cord. Dev Biol. 2017;432:3–13. 10.1016/j.ydbio.2017.01.021. 28192080 10.1016/j.ydbio.2017.01.021

[72] Maier EC, Saxena A, Alsina B, Bronner ME, Whitfield TT. Sensational placodes: neurogenesis in the otic and olfactory systems. Dev Biol. 2014;389(1):50–67. 10.1016/j.ydbio.2014.01.023. Epub 2014 Feb 6. PMID: 24508480; PMCID: PMC3988839. 24508480 10.1016/j.ydbio.2014.01.023 PMC3988839

[73] Wymeersch FJ, Skylaki S, Huang Y, Watson JA, Economou C, Marek-Johnston C, Tomlinson SR, Wilson V. Transcriptionally dynamic progenitor populations organised around a stable niche drive axial patterning. Development. 2019;146(1):dev168161. 10.1242/dev.168161. PMID: 30559277; PMCID: PMC6340148. 30559277 10.1242/dev.168161 PMC6340148

[74] Balmer S, Nowotschin S, Hadjantonakis AK. Notochord morphogenesis in mice: Current understanding & open questions. Dev Dyn. 2016;245(5):547–57. 10.1002/dvdy.24392. Epub 2016 Mar 14. PMID: 26845388; PMCID: PMC4844759. 26845388 10.1002/dvdy.24392 PMC4844759

[75] Henrique D, Abranches E, Verrier L, Storey KG. Neuromesodermal progenitors and the making of the spinal cord. Development. 2015;142(17):2864–75. 10.1242/dev.119768. PMID: 26329597; PMCID: PMC4958456. 26329597 10.1242/dev.119768 PMC4958456

[76] Wilson V, Olivera-Martinez I, Storey KG. Stem cells, signals and vertebrate body axis extension. Development. 2009;136:1591–604. 10.1242/dev.021246. 19395637 10.1242/dev.021246

[77] Wang K, Hou L, Wang X, Zhai X, Lu Z, Zi Z, Zhai W, He X, Curtis C, Zhou D, Hu Z. PhyloVelo enhances transcriptomic velocity field mapping using monotonically expressed genes. Nat Biotechnol. 2023. 10.1038/s41587-023-01887-5. Epub ahead of print. PMID: 37524958. 10.1038/s41587-023-01887-5 37524958

[78] Rajewsky N, Almouzni G, Gorski SA, et al. LifeTime and improving European healthcare through cell-based interceptive medicine. Nature. 2020;587:377–86. 10.1038/s41586-020-2715-9. 32894860 10.1038/s41586-020-2715-9 PMC7656507

[79] Lickert H, Kispert A, Kutsch S, Kemler R. Expression patterns of Wnt genes in mouse gut development. Mech Dev. 2001;105(1–2):181–4. 10.1016/s0925-4773(01)00390-2. PMID: 11429295. 11429295 10.1016/s0925-4773(01)00390-2

[80] Tong X, Xia Z, Zu Y, Telfer H, Hu J, Yu J, Liu H, Zhang Q, Sodmergen, Lin S, Zhang B. ngs (notochord granular surface) gene encodes a novel type of intermediate filament family protein essential for notochord maintenance in zebrafish. J Biol Chem. 2013;288(4):2711–20. 10.1074/jbc.M112.379172. Epub 2012 Nov 6. PMID: 23132861; PMCID: PMC3554937. 23132861 10.1074/jbc.M112.379172 PMC3554937

[81] Li L, Chen M, Liu W, Tai P, Liu X, Liu J-X. Zebrafish cox17 modulates primitive erythropoiesis via regulation of mitochondrial metabolism to facilitate hypoxia tolerance. FASEB J. 2022;36:e22596. 10.1096/fj.202200829R. 36208295 10.1096/fj.202200829R

[82] Santhanam A, Shihabeddin E, Wei H, et al. Molecular basis of retinal remodeling in a zebrafish model of retinitis pigmentosa. Cell Mol Life Sci. 2023;80:362. 10.1007/s00018-023-05021-1. 37979052 10.1007/s00018-023-05021-1 PMC10657301

[83] Tossell K, Andreae LC, Cudmore C, Lang E, Muthukrishnan U, Lumsden A, Gilthorpe JD, Irving C. Lrrn1 is required for formation of the midbrain-hindbrain boundary and organiser through regulation of affinity differences between midbrain and hindbrain cells in chick. Dev Biol. 2011;352(2):341–52. 10.1016/j.ydbio.2011.02.002. Epub 2011 Feb 18. PMID: 21315708; PMCID: PMC3084456. 21315708 10.1016/j.ydbio.2011.02.002 PMC3084456

[84] Jia W, Zhang Y, Wang X, Luo L, Sun H, Jiang Y, Wang J, Mao Q, Guo Y, Kong L, Mo R, Li C. KRT5 mutation regulate melanin metabolism through notch signalling pathway between keratinocytes and melanocytes. Exp Dermatol. 2023;32(6):752–65. 10.1111/exd.14761. Epub 2023 Mar 4. PMID: 36809573. 36809573 10.1111/exd.14761

[85] Zhang T, Yao S, Wang P, Yin C, Xiao C, Qian M, Liu D, Zheng L, Meng W, Zhu H, Liu J, Xu H, Mo X. ApoA-II directs morphogenetic movements of zebrafish embryo by preventing chromosome fusion during nuclear division in yolk syncytial layer. J Biol Chem. 2011;286(11):9514–25. 10.1074/jbc.M110.134908. Epub 2011 Jan 6. PMID: 21212265; PMCID: PMC3058998. 21212265 10.1074/jbc.M110.134908 PMC3058998

[86] Lukoseviciute M, Mayes S, Sauka-Spengler T. Neuromesodermal Progenitor Origin of Trunk Neural Crest in vivo. Available at SSRN: https://ssrn.com/abstract=3902137 or 10.2139/ssrn.3902137.

[87] Martyna Lukoseviciute, Sarah Mayes, Tatjana Sauka-Spengler.

[88] Dawes JHP, Kelsh RN. Cell fate decisions in the neural crest, from pigment cell to neural development. Int J Mol Sci. 2021;22(24):13531. 10.3390/ijms222413531. PMID: 34948326; PMCID: PMC8706606. 34948326 10.3390/ijms222413531 PMC8706606

[89] Tesoriero C, Greco F, Cannone E, Ghirotto F, Facchinello N, Schiavone M, Vettori A. Modeling human muscular dystrophies in zebrafish: mutant lines, transgenic fluorescent biosensors, and phenotyping assays. Int J Mol Sci. 2023;24(9):8314. 10.3390/ijms24098314. PMID: 37176020; PMCID: PMC10179009. 37176020 10.3390/ijms24098314 PMC10179009

[90] Kovacic JC, Dimmeler S, Harvey RP, Finkel T, Aikawa E, Krenning G, Baker AH. Endothelial to mesenchymal transition in cardiovascular disease: JACC state-of-the-art review. J Am Coll Cardiol. 2019;73(2):190–209. 10.1016/j.jacc.2018.09.089. PMID: 30654892; PMCID: PMC6865825. 30654892 10.1016/j.jacc.2018.09.089 PMC6865825

[91] Arciniegas E, Neves CY, Carrillo LM, Zambrano EA, Ramírez R. Endothelial-mesenchymal transition occurs during embryonic pulmonary artery development. Endothelium. 2005;12(4):193–200. 10.1080/10623320500227283. PMID: 16162442. 16162442 10.1080/10623320500227283

[92] Alvandi Z, Bischoff J. Endothelial-mesenchymal transition in cardiovascular disease. Arterioscler Thromb Vasc Biol. 2021;41(9):2357–69. 10.1161/ATVBAHA.121.313788. Epub 2021 Jul 1. PMID: 34196216; PMCID: PMC8387428. 34196216 10.1161/ATVBAHA.121.313788 PMC8387428

[93] Palis J. Primitive and definitive erythropoiesis in mammals. Front Physiol. 2014;5:3. 10.3389/fphys.2014.00003. PMID: 24478716; PMCID: PMC3904103. 24478716 10.3389/fphys.2014.00003 PMC3904103

[94] Lange L, Morgan M, Schambach A. The hemogenic endothelium: a critical source for the generation of PSC-derived hematopoietic stem and progenitor cells. Cell Mol Life Sci. 2021;78(9):4143–60. 10.1007/s00018-021-03777-y. Epub 2021 Feb 9. PMID: tham33559689; PMCID: PMC8164610. 33559689 10.1007/s00018-021-03777-y PMC8164610

