# Supplementary material for: StaVia: spatially and temporally aware cartography with higher-order random walks for cell atlases
Source: Genome Biol. 2024 Aug 16;25:224. doi: 10.1186/s13059-024-03347-y (PMC11328412; doi:10.1186/s13059-024-03347-y)
Supplement: Supplementary file 1 — Additional file 1. Additional figures, notes and tables [65–94]. [file 13059_2024_3347_MOESM1_ESM.docx]

**StaVia: spatially and temporally aware cartography with higher order random walks for cell atlases**

**Shobana V. Stassen^1^, Minato Kobashi^2^, Edmund Y. Lam, Yuanhua Huang^2,5^ , Joshua W. K. Ho^2,4^ and Kevin K. Tsia^1,3^**

## Additional File 1

### List of Figures

Fig.S0A. Spatial-temporal parameters (Mouse Gastrulation)

Fig.S0B. Fig.S0B Atlas View Visualization Parameters (Zebrahub)

Fig.S0C. Cluster graph resolution parameters.

Fig.S1. Comparing TI graph structure of StaVia and CellRank+PAGA

Fig.S2. Lineage paths and gene trends for cell fates in StaVia using memory

Fig.S3. Lineage paths and gene trends comparison between StaVia and CellRank

Fig.S4. StaVia Memory helps distinguish gene trends near the NMP lineages

Fig.S5. Stability analysis for memory

Fig.S6. CellRank lineage probabilities not improved by projecting on Atlas View

Fig.S7. Zebrahub mesoderm: Comparison of lineage paths and gene specificity trends

Fig.S8. Zebrahub neural ectoderm: Comparison of lineage paths and gene trends

Fig.S9. Zebrahub non-neural ectoderm: Comparison of lineage paths and gene trends.

Fig S10. Impact of Memory on Mesodermal Lineage Probabilities

Fig S11. Impact of Memory on Mesodermal Gene Trends (Zebrahub)

Fig.S12 Comparison of visualization methods on time-series data (colored by tissue type)

Fig.S13 Comparison of visualization methods on time-series data (colored by stage)

Fig.S14 Impact of key steps in StaVia Atlas View embedding

Fig.S15 Impact of Steps in StaVia Embedding (cells colored by developmental stage)

Fig.S16 Impact of Steps in StaVia Embedding (cells colored by tissue type)

Fig.S17 Impact of Steps in StaVia Embedding (Radar plots)

Fig.S18 Second order random walk with memory

Fig.S19 Comparison to PAGA, Palantir and CellRank for Spatial data

Fig.S20 StaVia for Zesta - with and without spatial coordinates

### List of Notes and Tables

Note S1: Parameter guidance for StaVia

Note S2: StaVia reveals 3 developmental patterns in Murine Embryogenesis

Note S3: Selection rationale of TI methods used for comparison

Table S1 for parameters used in TI

Table S2 for parameters used for single-cell embeddings

Table S3a-3d for literature support for StaVia biological findings

**Note S1: Parameter Guidance for StaVia**

This section covers the main parameters influencing TI and visualization in StaVia and can be used in conjunction with the tutorials available on StaVia’s ReadTheDocs pages to help inform the user on parameter selection and impact. We inspect parameters both quantitatively - through correlation analysis to assure the user of stable behaviour, and also qualitatively - through visualized outputs on incremental changes to parameters - which we believe is intuitive and useful for potential users.

**Spatial and Temporal metadata related parameters:**

There are two main temporal parameters that impact how the temporal information is incorporated into the TI and resulting visualizations. These are the knn_temporal (number of sequentially adjacent neighbors added between cells) and time_diff (edges > time_diff apart are removed from the single-cell graph)*.* The main spatial parameter is knn_spatial.

- **Knn-temporal: Supplementary Fig.S0Aa** shows how the interpretation of the trajectory alters based on the number of knn_temporal. We illustrate this using the Atlas View which is derived from both the underlying single-cell and cluster-graph configurations. Adding knn-temporal refines the sequential connectivity, with the marginal change for this dataset stabilizing after knn_temporal >10. **Supplementary Fig.S0Ab** shows that the inferred pseudotime is very stable predominantly only showing a noticeable change when moving from no time-series information towards using the time-series labels to guide the TI.
- **Time_diff:** This parameter causes fairly subtle changes as the time_diff threshold is only applied as long as a cell continues to maintain more than 5 edges. A time_diff =$\tau$ means that edges between cells that are more than =$\tau$ discrete average time intervals apart will be removed. In **Supplementary Fig.S0Ab,** For $\tau<5$, the hematopoietic branches maintain connectivity to the early mesoderm as edges within these time-intervals represent a greater share of the total edges present. This connectivity between hemato-early mesoderm is lost for greater values of $\tau$.
- **Knn-spatial:** In datasets with spatial coordinates, we use the knn_spatial parameter in two distinct steps of the StaVia TI computations. StaVia's graph construction leverages a recent concept [V. Singhal 2024] to recalibrate gene expression by considering a cell's environment of its knn_spatial tissues-location based neighbors. Furthermore, StaVia also augments the gene-expression based KNN graph with knn_spatial spatial neighbors when establishing cluster connectivity. **Supplementary Fig.S0Ae** shows how the number of knn_spatial spatial neighbors impacts the ability to delineate cell types at early stages of development, which aids in the construction of pathways towards cell fates.

**Memory:** We summarize the key figures used to show the impact of increasing levels of memory on recovering lineage pathways and gene trends for all cell fates in two large and complex datasets. Generally speaking, higher memory increases the specificity of lineage pathways towards cell fates and prevents the inclusion or unrelated intermediate states. However, very large values can potentially be too restrictive. **Fig.4a** in the main text shows the impact on a subset of recovered pathways, and **Fig.4b** shows the consequential impact on gene trends which use these lineage probabilities to predict the gene trend associated with each lineage along pseudotime. **Fig4. and Fig.S10-S11** present these results more exhaustively for all lineages for both Zebrahub and Mouse gastrulation. **Fig.S5a** quantifies the stability when tuning the memory parameter. **Fig.S5b** offers a heuristic for quantitatively identifying a range of memory values, however this works when temporal data is available.

**Edge directions using scRNA velocity:** The scRNA velocity can be used to determine the direction of edges. However, given these readings can be very noisy and at times velocity models are incompatible with the biology [Zheng, S.C et al., 2023, Bergen V. et al., 2021], we allow for the directionality to be set as a weighted average of pseudotime based direction, and scRNA velocity direction by using the parameter velocity_weight. Directionality is added to the original feature-based symmetric/undirected transition matrix $T_{undirected}$by biasing the relative weighting of ${edge}_{ij}$ (from ${cell}_{i}$ to ${cell}_{j}$) versus the edge${edge}_{ji}$based on pseudotime and RNA velocity. The final transition matrix is given by:

$({weight}_{velo} \times T_{velo}) +((1-{weight}_{velo}) \times T_{pseudotime})$.

The default value when scRNA-velocity is available, is set to velocity_weight = 0.5 such that both pseudotime and scRNA-velocity contribute equally to the edge directionality. If the dataset possesses scRNA-velocity available for a large number of genes, the weight can be increased such that velocity_weight $\geq0.5$. Conversely, if the RNA velocity data is thought to be noisy, have transcriptional boosts and incompatible with model assumptions of time-constant rates of splicing/degradation and transcription [Zheng, S.C et al., 2023], then a de-emphasis on the directionality inferred by scRNA-velocity may be suitable provided there is a clear subset of cells which represent an initial state.

It should also be noted that when RNA velocity is available, the root can be auto-detected. StaVia proposes 3 root states for the user to consider when defining the start state (if no user-feedback is, the first of these roots is selected, as the cluster with the lowest stationary probability). When prior knowledge is available and preferred, then the root can be user-defined.

**Cluster graph resolution and connectivity parameters:** The key parameters which influences the resolution of the clusters-graph is the number of k-nearest neighbors of the single-cell expression graph (knn), the number of features of the input and the level of edge-pruning prior to clustering (edgepruning_clustering_resolution) of the single-cell graph prior to using a graph based clustering method such as PARC (default). **Fig.S0C** shows how on a scRNA-seq dataset of hematopoiesis (Setty et al, 2019), that increasing knn lowers the number of clusters by globally increasing the connectivity between cells thus making them ‘harder’ to separate. By pruning the graph edges using edgepruning_clustering_resolution, we can more locally adjust the overall connectivity of the graph to remove spurious edges at local regions of the graph and resolve a greater number of clusters or sub-types for a given knn without causing graph fragmentation that can otherwise occur for lower global knn values (see PARC Stassen 2020 for a detailed explanation of this parameter).

We note that the temporal parameters (knn_temporal and time_diff) do not influence the number of clusters and are used only to modulate the connectivity of the single-cell and cluster graphs, and done separately from the clustering step. The rationale for this is that temporal and spatial edges are primarily used to examine transitions, and we want to avoid artificially forcing them to cluster together. We refer to figures from Via 1.0 [Stassen 2021] for a detailed analysis on multiple datasets of parameters such as knn, and dimensionality of input data (we have included an overview figure here for completeness).

**Atlas View parameters:** The Atlas View is a key feature of StaVia and the final visualized output can be tuned using a handful of key parameters. It is important to note that altering the visualized resolution of edges and cells does not impact the underlying TI which is influenced by the other parameters discussed earlier in this section. The results are presented in **Fig.S0Ba-d**

- Global_visual_pruning: increasing this parameter increases the number of edges shown
- Decay: increasing the decay increases the merging of edges
- Initial_bandwidth: increasing the initial bandwidth increases merging of edges.
- N_milestones: increasing n_milestones increases the resolution of edges on the Atlas View

**
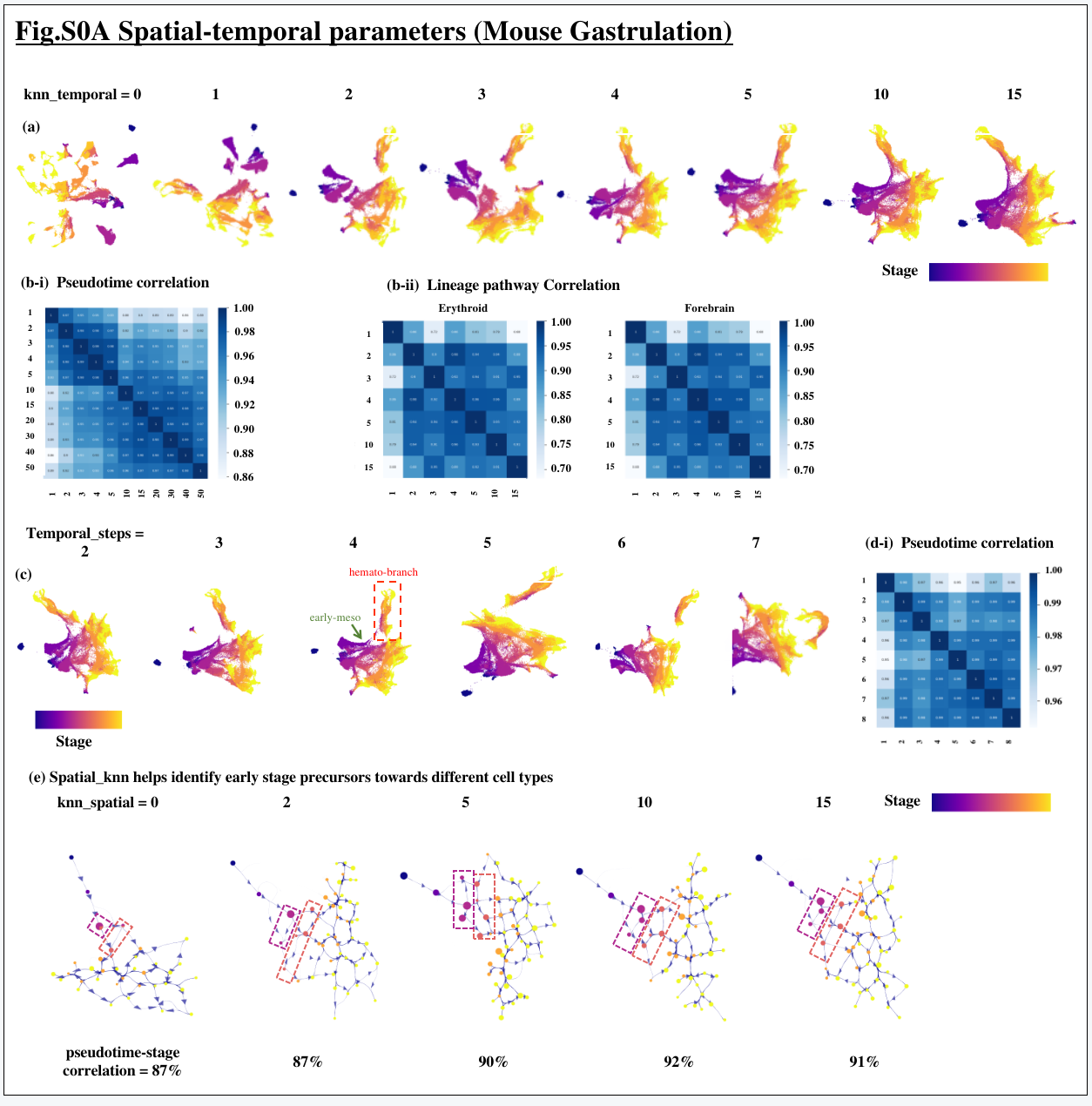
**

***Fig.S0A Spatial-temporal parameters (Mouse Gastrulation)*** *(a) Adding knn-temporal refines the sequential connectivity, with the marginal change for this dataset stabilizing after knn_temporal >10. (b) inferred pseudotime and lineage probabilities are very stable, predominantly showing a noticeable change when moving from no time-series information towards using the time-series labels to guide the TI. (c) Varying time_diff causes subtle changes in the topology. In this example, for < 5, the hematopoietic branches maintain connectivity to the early mesoderm as edges within these time-intervals represent a greater share of the total edges present. This connectivity between hemato-early mesoderm is lost for greater values of 𝛕. (d) Pseudotime correlation is very high across this parameter. (e) shows how adding knn_spatial spatial neighbors improves the ability to delineate cell types at early stages of development, which aids in the construction of pathways towards cell fates. The dark-pink and burnt-orange colored boxes house a greater number of early stage clusters as spatial neighbors are accounted for in the cluster graph. Without spatial neighbors (knn_spatial =0), all early stage cells are placed in a few common large clusters, with no cell type distinction.*

**
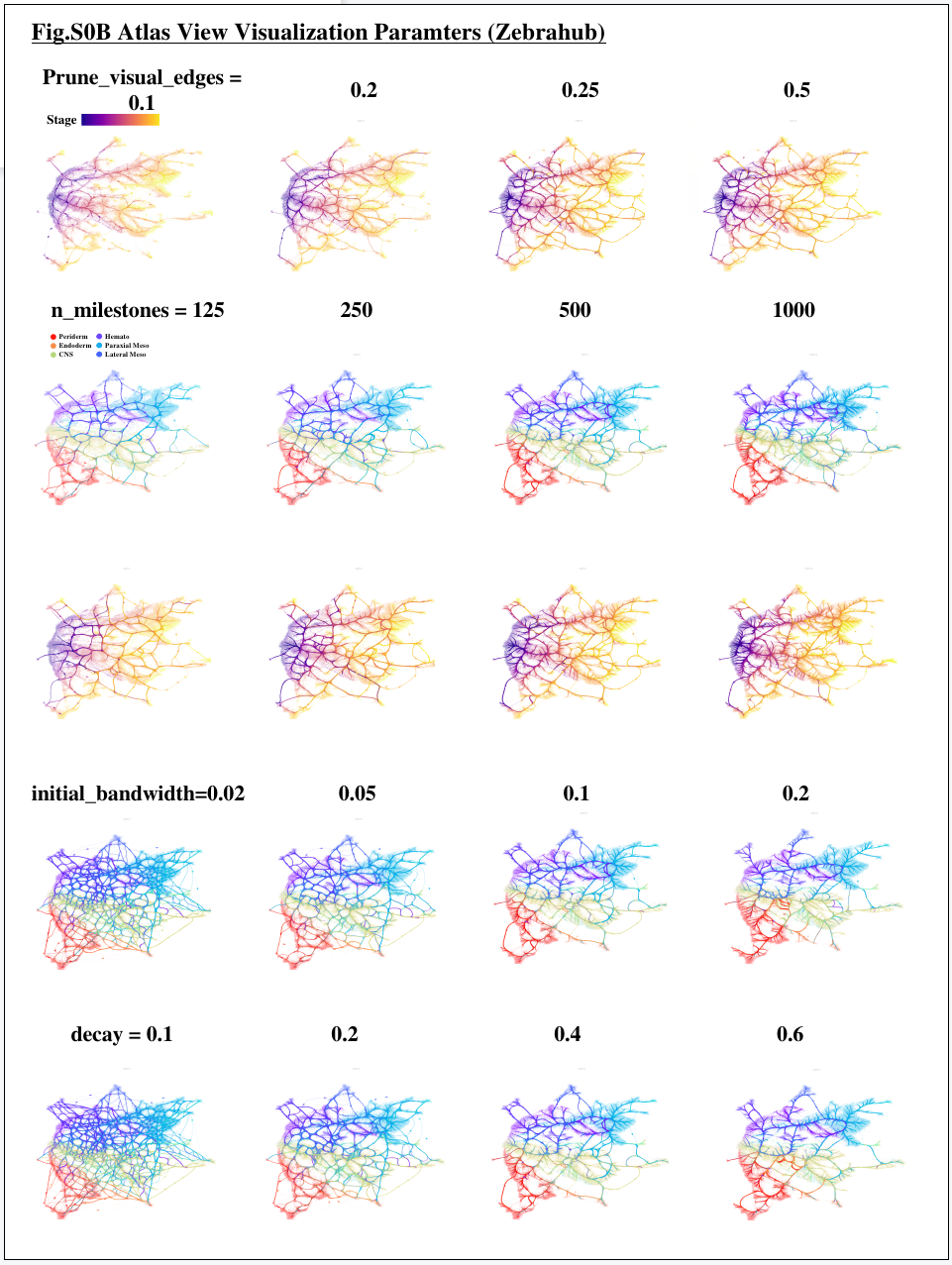
**

***Fig.S0B Atlas View Visualization Parameters (Zebrahub)*** Shows how four key parameters impact the visualized resolution of edges. Unless indicated as per the figure, prune_visual_edges = 0.25, initial_bandwidth=0.1, n_milestones =500, decay = 0.4

**
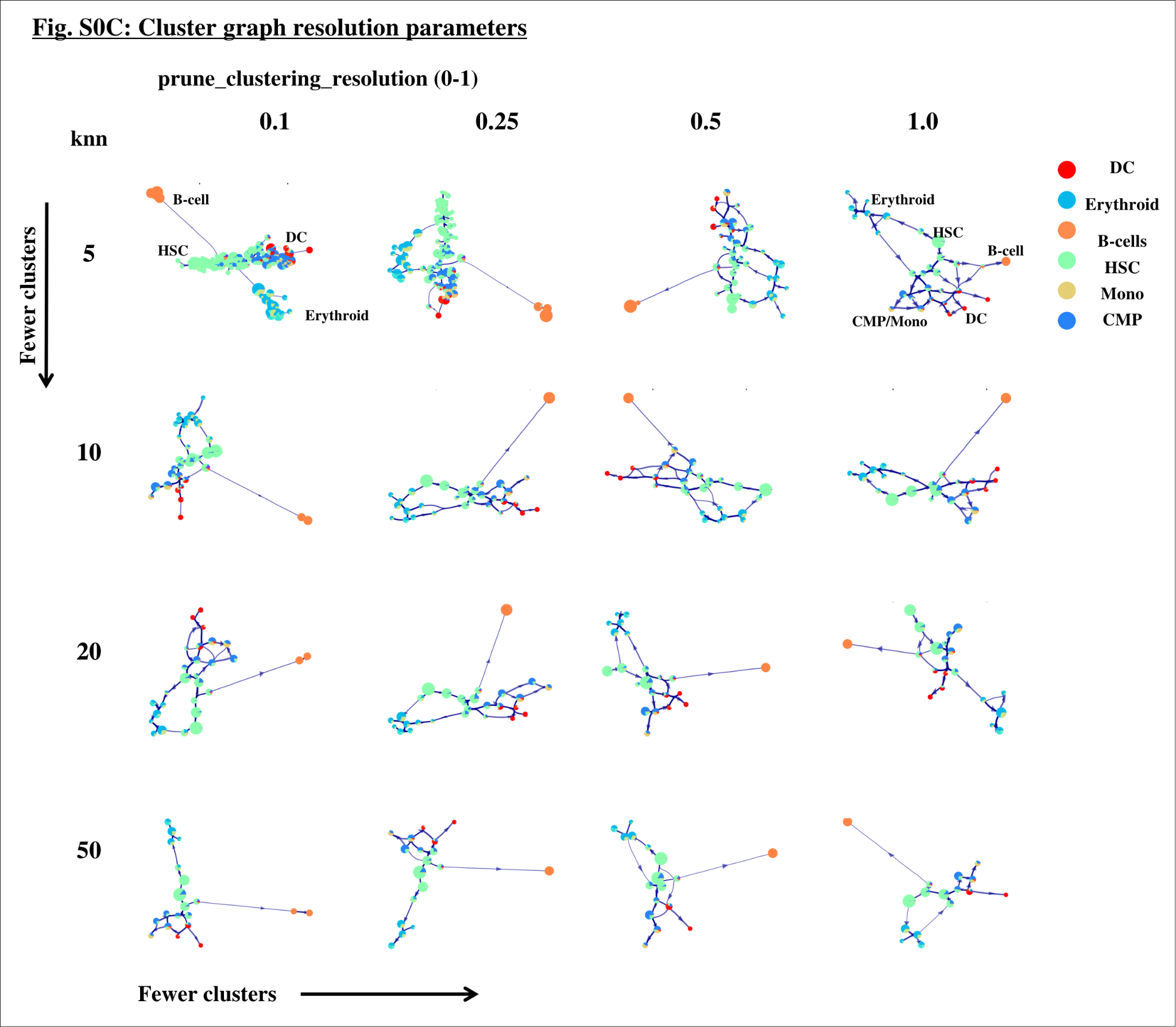
**

***Fig.S0C Cluster graph resolution parameters.*** Increasing the number of k-nearest neighbors reduces the number of clusters. In this dataset we do not observe a noticeable increase in the number of edges as knn is increased which suggests that connectivity is expressed predominantly within cell types and less between different cell types (the graph is highly modular). Increasing prune_clustering_resolution, retains more edges and thus lowers the number of clusters found in the graph partitioning.

**Note S2: StaVia reveals developmental structures in Mouse Gastrulation using its directed graph layout**

Here we highlight three novel developmental transitions in the Sala 2019 mouse gastrulation atlas, (with reference to **Fig.2 and Fig.3a in Results**) not automatically captured in other TI methods nor clearly visualized in other representations of the full dataset.

**1. Hematopoietic waves:** The Atlas View and the cluster graph reveal two waves of hematopoiesis - primitive and pro-definitive. The spatial arrangement of cells on these graphs in terms of adjacent populations and temporal stage is consistent with knowledge that the transient primitive (first) wave arises shortly after mesoderm formation [L Lange & Morgan 2021, Palis 2014]. We see this in Edge A of the StaVia cluster graph **(Fig.S1a)**. Edge B is a minor edge where nascent mesoderm cells are redirected towards the main mesodermal branch instead of proceeding towards the hematopoietically specified branch. In StaVia, the hematopoietic potential of the pro-definitive (second wave) is marked by an E8.5 population of hemogenic endothelial cells which undergo an endothelial-to-hematopoietic transition (EndHT)[Canu & Ruhrberg 2021, Wu Y & Hirschi 2020]. Amongst the hemato-endothelial *Kdr+Cd34+* progenitors **(Fig. 2b)**, the cells with lower *Etv2* (a hematopoiesis suppressor) are perhaps those most poised for EndHT [Hayashi 2012, Shen 2021]. This transition is captured by Edge C,which extends from the hemato-endothelial clusters towards the hematopoietic branch and represents the onset of definitive hematopoiesis initiated by hemogenic endothelial cells. Edge D extends from the hemato-endothelial clusters towards the paraxial and lateral plate mesoderm and represents endothelial-mesenchymal transitions (EndMT) of non-hemogenic endothelial cells towards the development of the arteries, heart and vasculature [Arciniegas 2005, Kovacic 2019].

**2. NMPs at the neuro-mesodermal junction:** The Atlas View (**Fig.2a,c**) depicts NMPs, bipotent progenitors expressing *T (Brachyury),* that contribute to spinal cord and paraxial mesoderm during axial elongation, arising from the caudal epiblast at E8.0 [Edri 2019, Cambray and Wilson, 2007; Wymeersch 2019]. NMPs closer to the paraxial mesoderm, residing on the upper section of the triangle marked in **Fig.2c**, express higher *Tbx6* expression, suggesting a pro-mesodermal tendency, consistent with the paraxial mesoderm cells neighboring this edge of the NMP-triangle. NMPs on the lower edge of the triangle express more *Nkx1-2*, correctly indicating a propensity towards the spinal cord neural cells that lie adjacent to them on the Atlas [Henrique 2015; Steventon & Arias 2017, Wilson 2009, Edri 2019]. **Fig.3d** shows that the sequential gene expression of gastrulation to NMP is automatically captured along the pseudotime axis for the NMP lineage.

**3. Gut development:** In contrast to prior analysis [Pijuan-Sala 2019] which required subsetting of the visceral and definitive endoderm (VE, DE) and gut cells *(Wnt5b+)* to observe the dual origin of the gut, StaVia’s cluster graph **(Fig.3a)** and Atlas View **(Fig. 2a,d)** reveal that gut endoderm morphogenesis arises from two distinct developmental origins: the intercalation of the streak derived *Sox17* positive definitive endoderm (DE) and the dispersal of *Ttr* expressing extraembryonic VE [Kwon 2008, Balmer, 2017, Nowotschin 2019].

**Note S3: Selection rationale of TI methods used for comparison**

We primarily focus on comparing StaVia to a hybrid pipeline of CellRank+PAGA+scVelo as this allows for a more apples-to-apples comparison with some key features in StaVia, such as:

1. Combining feature-distances, pseudotime and RNA velocity information to infer direction such that datasets without RNA-velocity (or where velocity is too noisy to be used exclusively) can still be analyzed. We note that the pseudotime-transition matrix in CellRank is computed using either Via 1.0 or Palantir. Data-driven strategies to automate the integration of spatial and temporal information, as done in StaVia, are not readily available in existing TI methods and therefore difficult to compare.
2. Dual interpretations of the TI and its visualization at cluster graph level and single-cell level (in terms of pseudotimes, cell fates and lineage probabilities) using the PAGA-CellRank cell-fate cluster graphs (**Fig.S1 iv and Fig. S6c** “cell fate view”) and scVelo-directed-PAGA graphs alongside, scVelo’s streamplots, scVelo-CellRank gene trends and CellRank’s single-cell lineage probabilities projected onto a UMAP/t-SNE. StaVia’s also offers a third type of view, the Atlas View, which offers a high-resolution view not just of the single-cells, but also the edges which make up the trajectories.

Palantir is used to represent comparison with methods (such as Monocle3, Slingshot, FateID, MARGARET) that operate solely on pseudotime without scRNA-velocity or other multi-modal metadata. It is also one of the more scalable examples of these methods, with runtimes in the order of ~10 minutes for 100K cells (compared to ~3 minutes for StaVia). In addition to CellRank, scVelo and directed-Paga, there are a handful of other recent RNA-velocity TI methods. These are Cytopath [Gupta 2022], Cellpath [Z. Zhang and X.Zhang 2021] and Vetra [G.Weng 2021].

With respect to our first criterion of offering a ‘hybrid’ approach that allows integration of non-RNA velocity-based and velocity-based direction inference, we note that these three methods rely exclusively on RNA-velocity to infer direction and are limited to use on small datasets of less than 10,000 cells. Cytopath relies on scVelo’s pipeline (or the user) to provide the transition probability matrix, the root and terminal states, and three elements that can substantially influence the accuracy of TI analysis. Because of their reliance on the quality of the RNA-velocity data, Vetra and Cellpath often suffer from poor root and terminal state detection [Gupta et al., 2022] and cannot accept user-defined roots/cell fates to provide adjustments, which can distort results. Cellpath also frequently grossly overestimates the number of trajectories that confound downstream analysis [Gupta et al., 2022] and require manual subselection.

With respect to our second criterion, we note that the pure RNA velocity and pure pseudotime-based methods mentioned above do not offer an intuitive way to liaise visually or computationally between the cluster graph and single-cell resolution trajectory which is a unique aspect of StaVia’s output. However, combining Paga+scVelo+CellRank allowed us to compare various facets of StaVia. We note that CellRank and Palantir required manual assignment of several cell fates automatically detected by StaVia. On a ~100K cell dataset, StaVia completes the full trajectory computation in 3 minutes, compared to Palantir’s 8 minutes and CellRank’s 20 minutes during which both Palantir and CellRank occupy all resources in a 8 Intel Zeon 3.6GHz CPUs machine. Pre-processing and sc-embedding computation runtime are excluded in these numbers. The computation time of scVelo’s latent time which can be used in CellRank’s gene trends is excluded from the reported runtime and takes (on the Mouse Gastrulation dataset) 105 minutes to compute on 8 cores, requiring 99 GB RAM, compared to StaVia’s pseudotime which computes in ~ 1 minute and requires less than half the peak RAM.

**Tables for parameters used in TI**

**Table S1a. Mouse Gastrulation:**

| **Method** | **Common** | **Unique Parameters** |
| --- | --- | --- |
| StaVia | PCs = 30  KNN =30 | Velo_weight = 0.5  Knn_seq = 10 (20knn +10knnseq = 30 knn total) |
| CellRank |  | N_macrostates = 15 (default = 10, but increasing to 15 improved results). Higher values than 15 took prohibitively long runtimes without improvement |
| PAGA |  | scVelo’s latent time for direction (uses CellRank’s initial and terminal states) |
| Palantir |  | Epiblast root state provided to match that in CellRank and StaVia. Terminal states manually assigned to facilitate comparison. Waypoints set to 1000 to allow high resolution |

**Table S1b. Zebrahub:**

| **Method** | **Common** | **Unique Parameters** |
| --- | --- | --- |
| StaVia | PCs = 100  KNN =15 | Velo_weight = 0.5, temporal knn = 5, knn = 10. Total knn = 15 |
| CellRank |  | N_macrostates = 15 (Higher values than 15 took prohibitively long runtimes without improvement) |
| PAGA |  | Direction based on scVelo’s latent time |
| Palantir |  | Root state provided to match that in CellRank and StaVia as paraxial mesoderm from earliest time point. Terminal states manually assigned to facilitate comparison. Waypoints set to 1000 to allow high resolution. |

**Table S1c. ZESTA:**

| **Method** | **Common** | **Unique Parameters** |
| --- | --- | --- |
| StaVia | PCs = 30  KNN =15  “deep_blastomere” set as root state | knn = 15. spatial_knn=10, temporal_knn=5 (total knn=30) |
| CellRank |  | N_macrostates = 15 (Higher values than 15 took prohibitively long runtimes without improvement). We tried both knn = 15 and knn=20, and found that knn=15 gave better resolution in CellRank. |
| PAGA |  | Direction based on pseudotime using blastomere as root |
| Palantir |  | Root state provided to match that in CellRank and StaVia as paraxial mesoderm from earliest time point. Terminal states manually assigned to facilitate comparison. Waypoints set to 1000 to allow high resolution. We tried knn = 15 and 20, and found knn=15 gave better resolution in Palantir. |

**Tables for parameters used in visualization**

All methods are run on default parameters. Non-default parameters are listed below.

**Table S2.**

| **Dataset** | **Common** | **StaVia** | **Umap** | **t-SNE** |
| --- | --- | --- | --- | --- |
| Mouse Gastrulation | PCs = 30  KNN =30 | min_dist=0.2  30knn=15seq +15 nonseq | min_dist=0.2 | Perplexity =30 |
| Zebrahub Lange | PCs = 100  KNN =30 | min_dist=0.3  30knn=15seq +15 nonseq | min_dist=0.3  knn=50 to reduce fragmentation | Perplexity =30 |
| Zebrafish Wagner | PCs = 30  knn=60 | min_dist=0.1  60knn=45seq +15 nonseq | min_dist=0.1 | Perplexity = 100 |
| Ascidian protovert Cao | PCs = 30  KNN =30 | min_dist=0.3  30knn=15seq +15 nonseq | min_dist=0.3 | Perplexity = 30 |
| Mouse Neuron Manno | PCs=30  knn=30 | min_dist=0.2  30knn=15seq +15 nonseq | min_dist=0.2 | Perplexity =30 |

**Tables for literature support of StaVia’s biological findings**

We provide a table for each of the main atlases analyzed by StaVia that summarizes the biological interpretations and the relevant literature support for these findings

**Table S3a. Mouse Gastrulation**

**
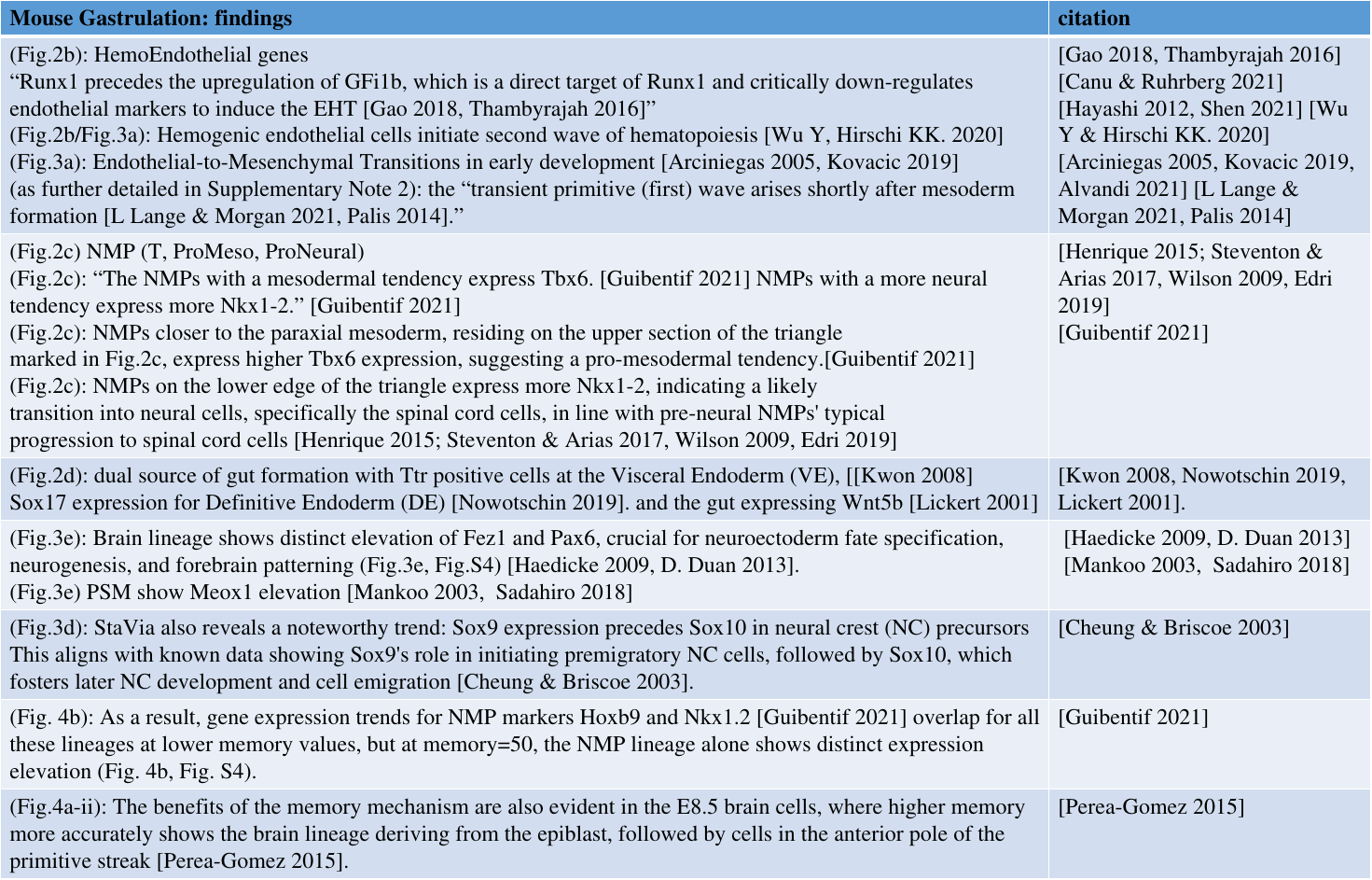
**

**Table S3b. Zebrahub**

**
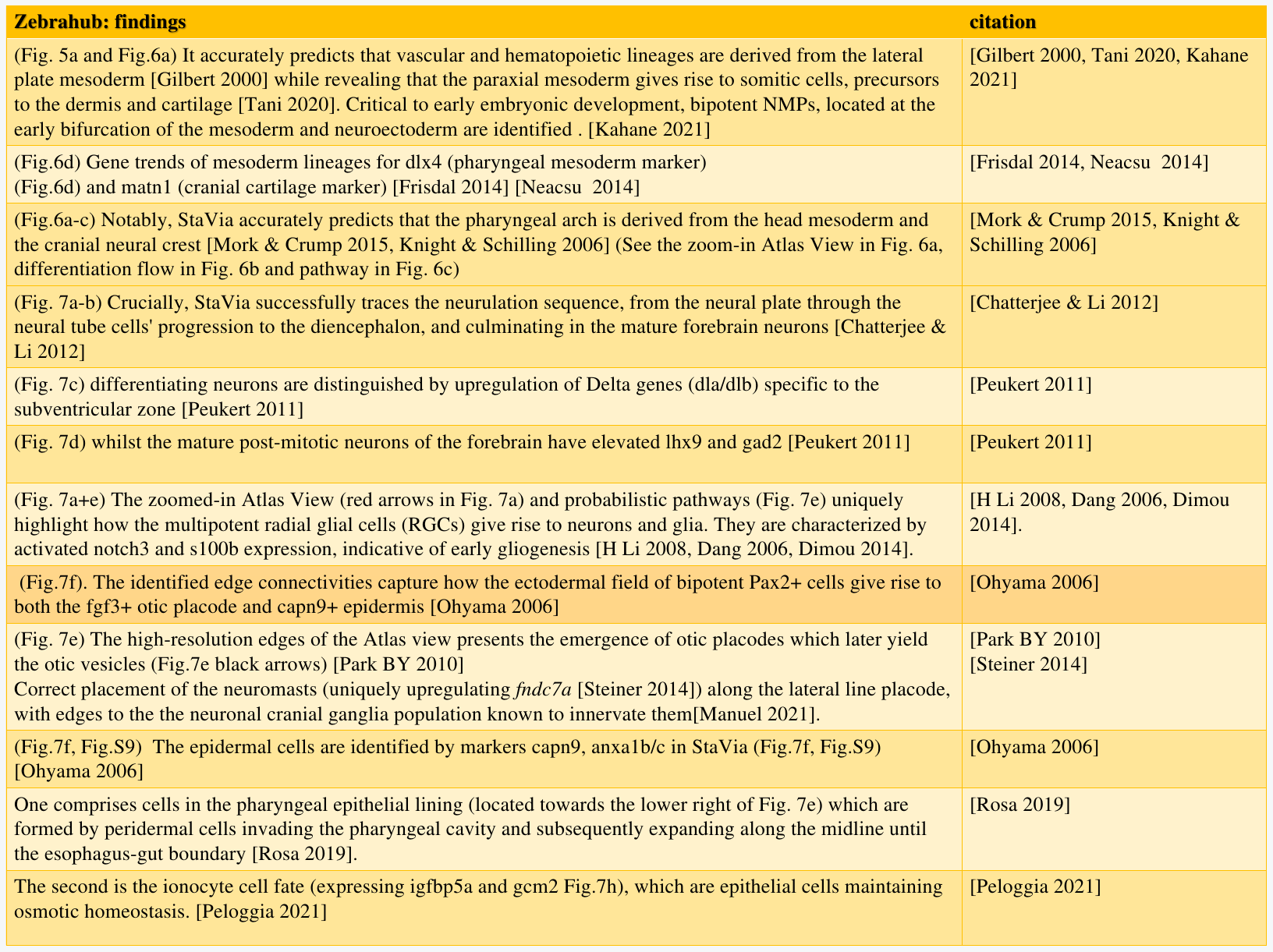
**

**Table S3c. MERFISH Preoptic Mouse**

**
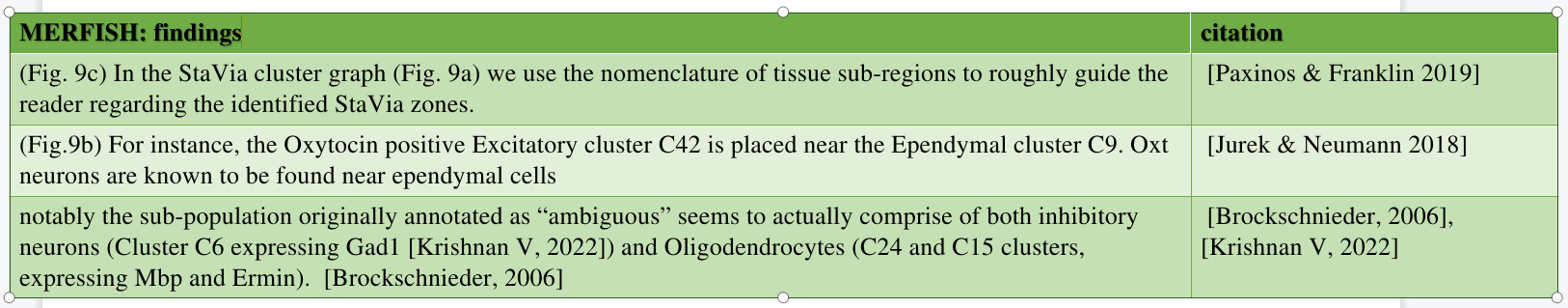
**

**Table S3d. Stereo-seq Zesta Zebrafish STOmics**

**
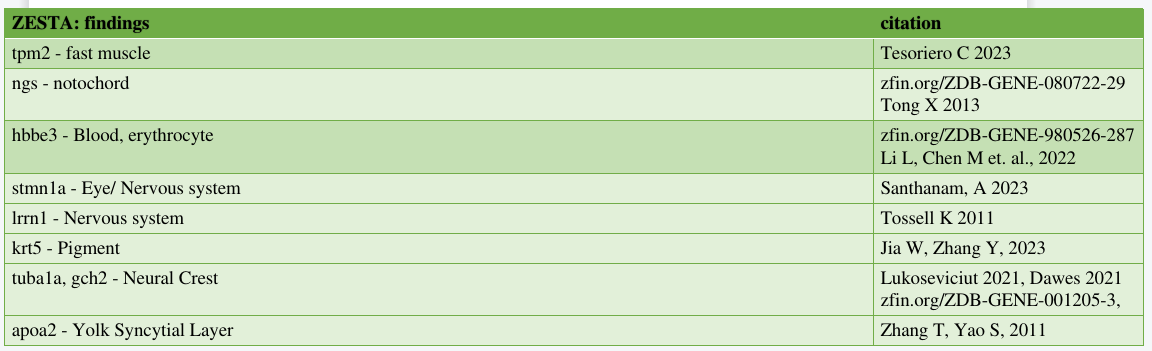
**

**
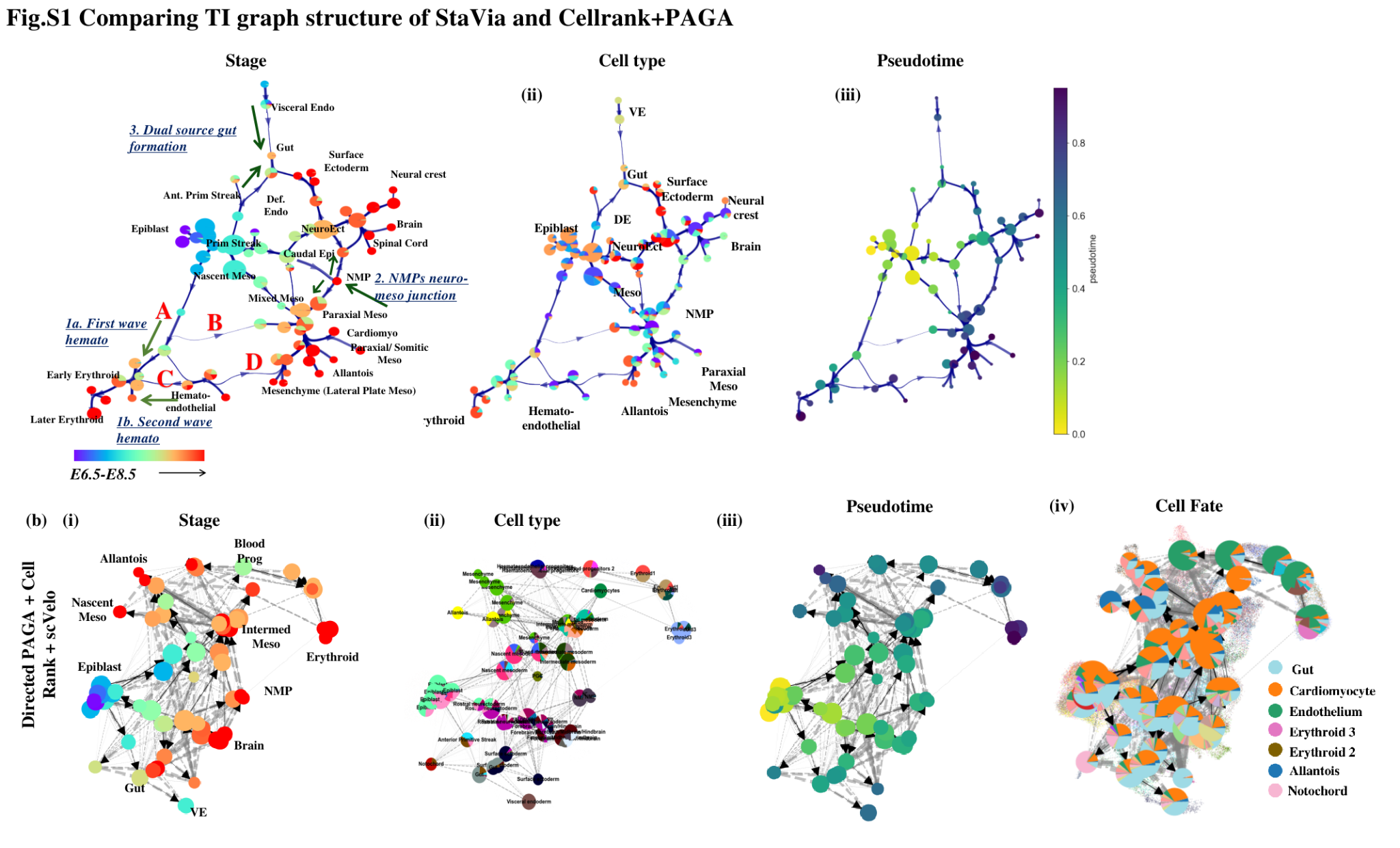
*Fig.S1. Comparing TI graph structure of StaVia and CellRank+PAGA*** *(a) StaVia pseudotime-RNA velocity directed cluster graph colored by (i) developmental stage (ii) cell type (iii) Via pseudotime (b) scVelo-latent time directed PAGA graph, initialized with CellRank’s initial states. Gray arrows denote connectivity and black arrow-edges denote direction, colored by (i) known developmental stage (ii) cell type (iii) Pseudotime (iv) CellRank’s lineage probability of cells in that cluster towards one of the detected terminal states. Due to most lineage probabilities being very localized, and a few being very diffuse over all cells (cardiomyocyte and surface ectoderm whose sc-likelihoods are shown in Fig.3 and Fig. S3), we see the cell fates associated with the diffused lineage probabilities over-represented in almost all clusters regardless of relevance to the cell fate.*

**
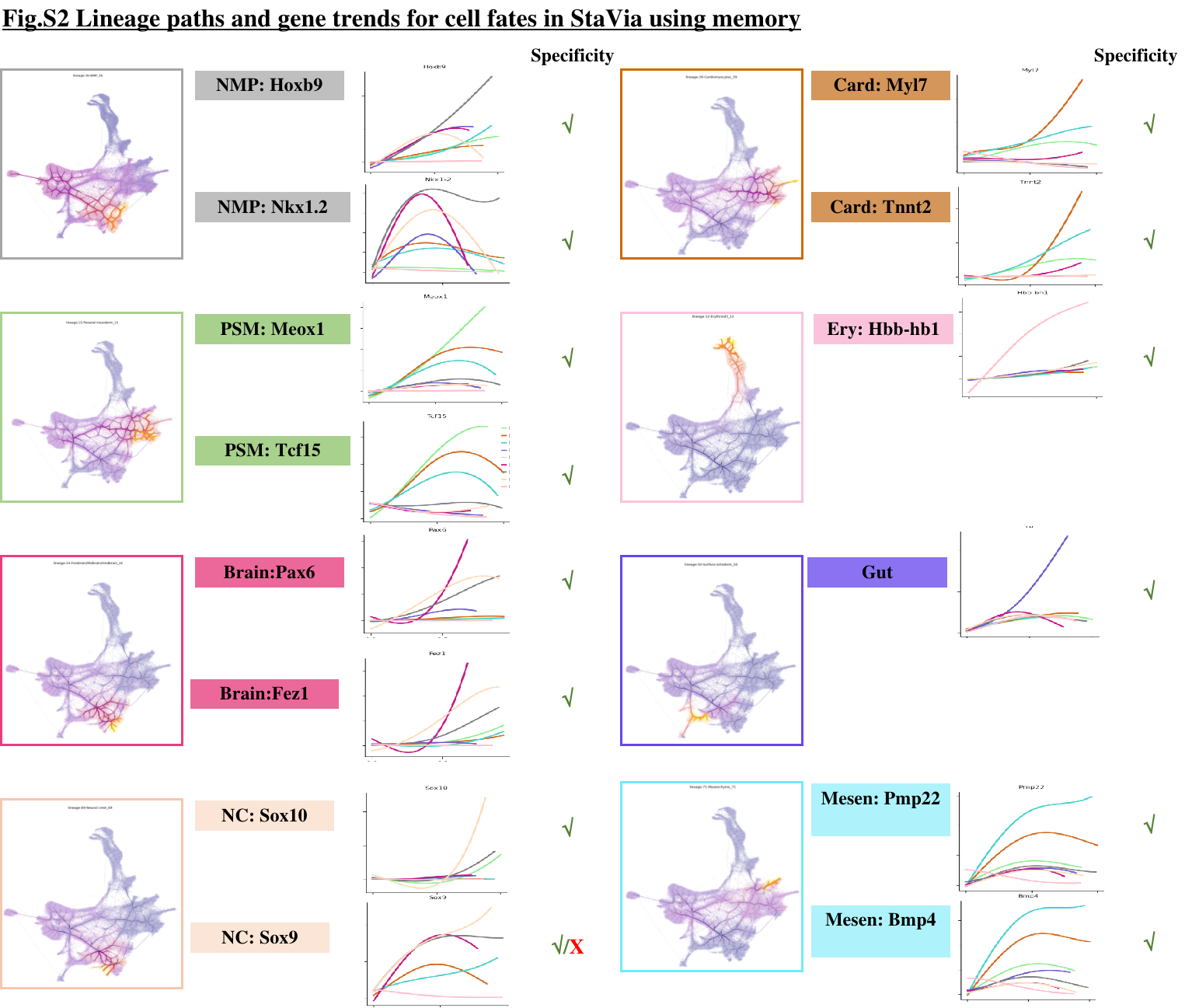
**

***Fig.S2. Lineage paths and gene trends for cell fates in StaVia using memory:*** *Lineage pathways detected by StaVia for cell fates show progression from epiblast (E6.5) through relevant intermediate stages before arriving at the cell fate population (E8.5). Two marker genes are shown for each lineage. Each plot shows the gene expression versus pseudotime for all lineages. The lineage trend of interest is indicated by the color of the gene-box and the border of the lineage plot. If the correct lineage shows upregulation of the highlighted marker gene, then the color of the trend line and gene label/plot border will match. E.g. The cardiomyocyte lineage is light brown. In the gene trends for Myl7 and Tnnt2, the brown cardiomyocyte trend is the single most upregulated compared to other trend lines belonging to the other lineages.*

**
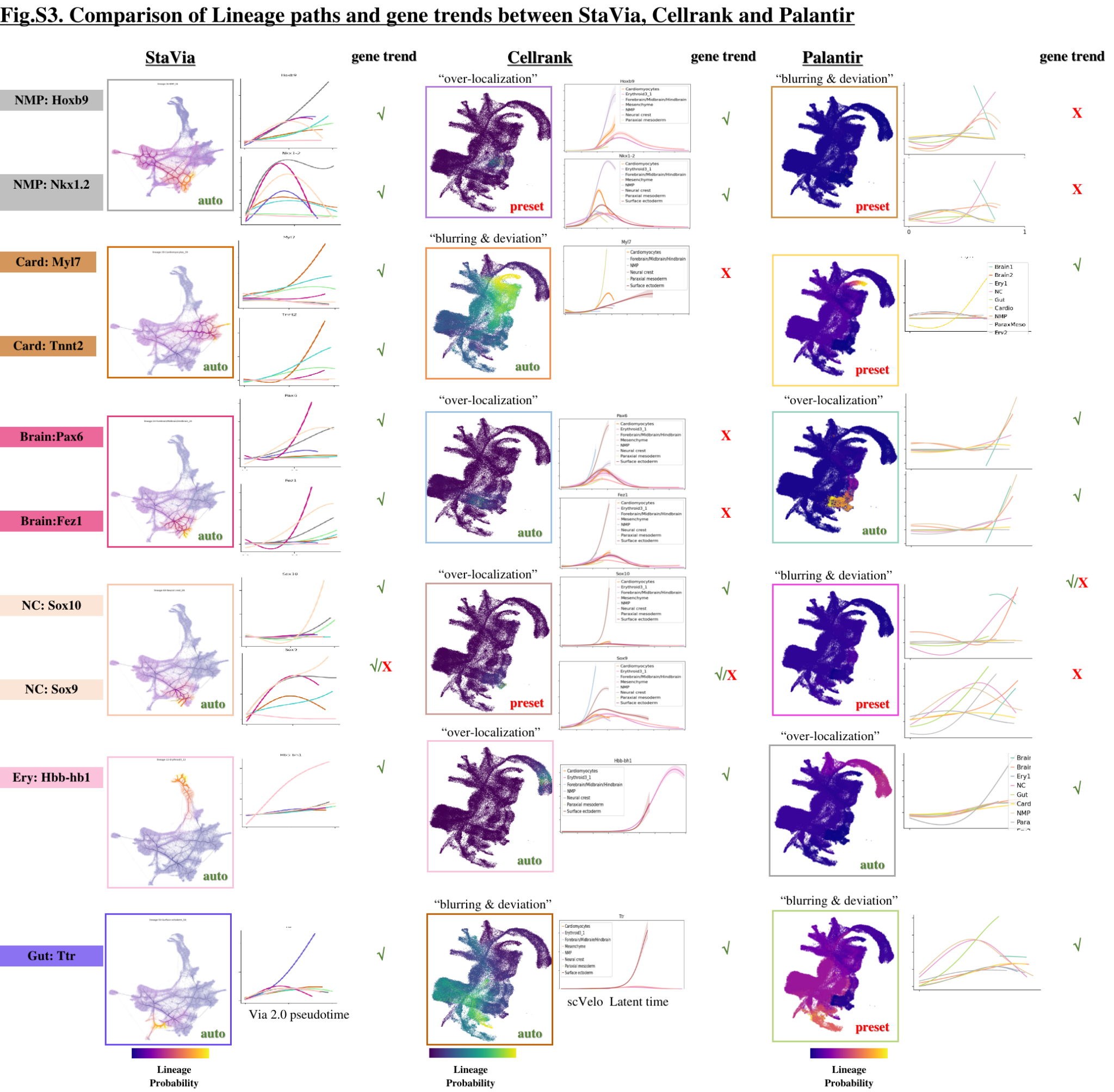
**

***Fig.S3 Lineage paths and gene trends comparison between StaVia, CellRank and Palantir.*** *StaVia’s lineage probabilities show paths from start-intermediate-end states, with the single-cell probabilities towards a given cell fate being visualized on the Atlas View. CellRank and Palantir lineage pathways (visualized on a UMAP embedding) are either very localized and show little information on the end-to-end progression from epiblast along consecutive stages, or are very diffuse (Gut), such that several cell populations are shown to progress towards the gut. Asides from not conveying the correct transitions, another consequence of this is that the gene trend lines associated with lineages are not correctly upregulated. For example, the Brain lineage trend line is light blue in CellRank (indicated by the border color of the pathway plot). However, the light blue gene trend line is not the most upregulated for Pax6 or Fez1 and hence receives a cross-mark in the columns titled “specificity”. scVelo’s latent time used for gene trends takes 105 minutes to compute on 8 cores and requires 99 GB RAM, compared to StaVia’s computes in ~ 1 minute and requires less than half the peak RAM.*

**
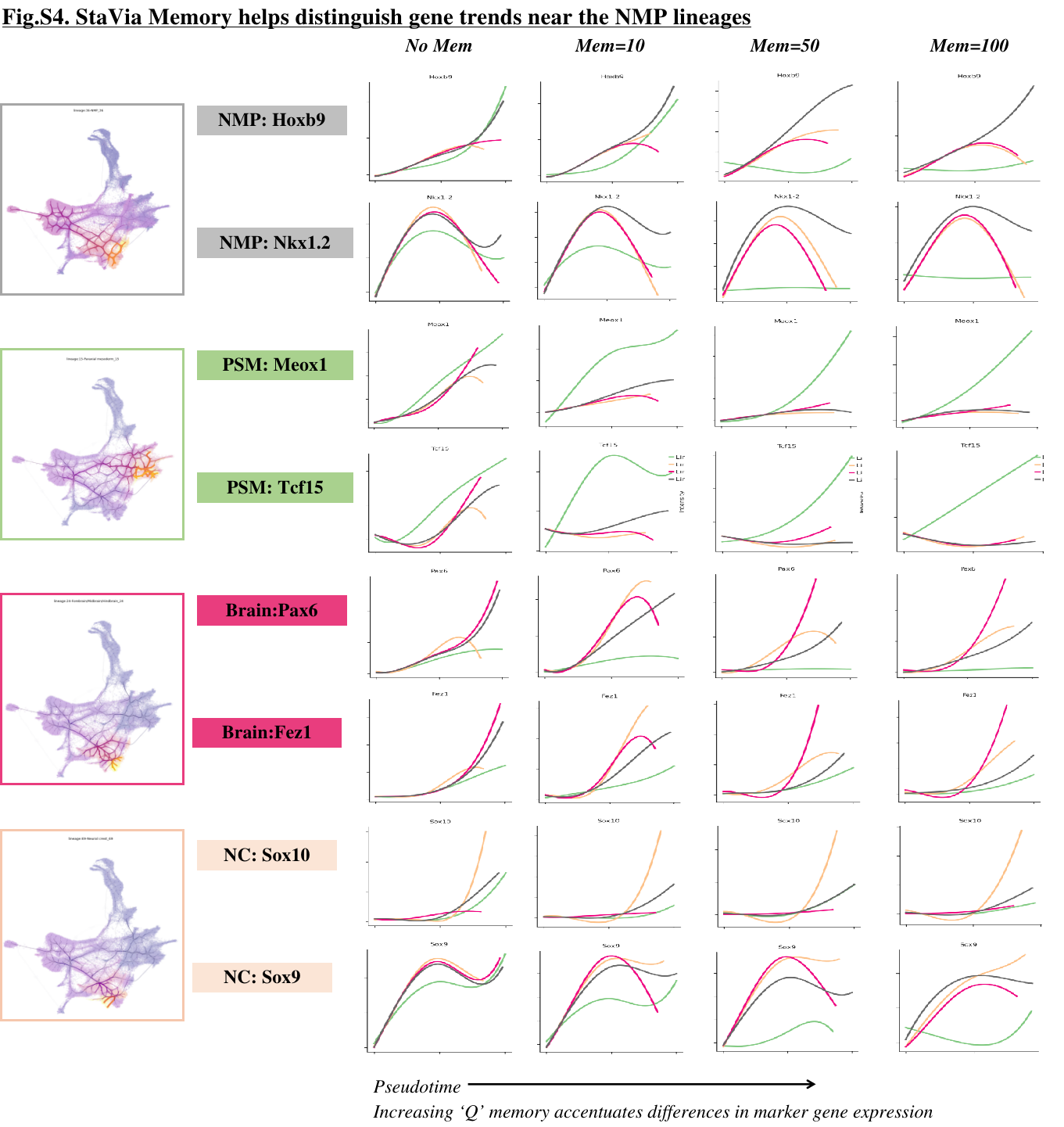
**

***Fig.S4. StaVia Memory helps distinguish gene trends near the NMP lineages:*** *Each gene trend subplot shows the gene expression along pseudotime for four lineages: NMP (neural mesodermal progenitors), PSM (Presomitic/paraxial mesoderm), Brain and NC (Neural crest) which are seen to emerge in varying proximity to each other. In particular with the NMPs closely linked to the brain and PSM populations, and the NC and Brain also being closely related. As a result, it can be challenging to obtain pathways that are specific enough to the relevant cell fates such that the correct gene trends for the relevant cell fate is plotted. By increasing memory, the correct lineage is upregulated for the marker gene, while other lineages remain suppressed.*


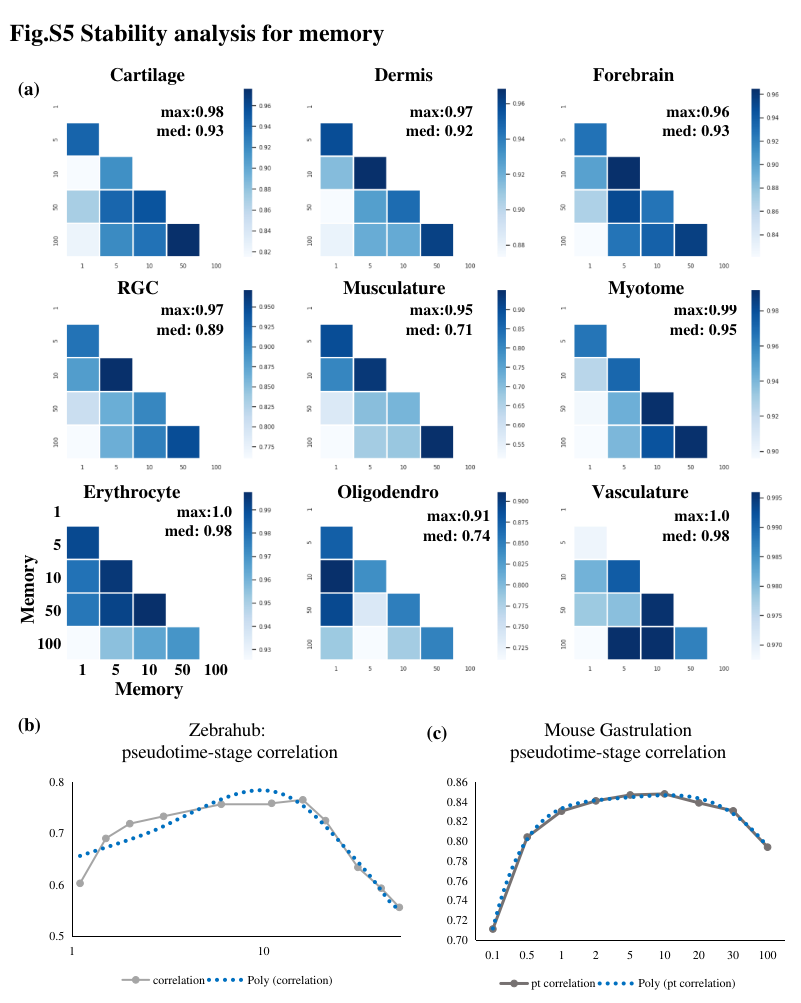


***Fig.S5 Stability analysis for memory*** *(a) pairwise correlation of lineage probabilities for Zebrahub cell fates when increasing memory from No memory (=1) to Memory =100 (b) for memory (value of 1 signified No memory, (b) correlation of stage-labels and inferred pseudotime of memory values 0.01-50. x-axis is plotted on log(1+memory) base10 scale. Shows that values between 5-20 are desirable for Zebrahub. (c) correlation between inferred pseudotime to Mouse gastrulation stages for different memory values.*

**
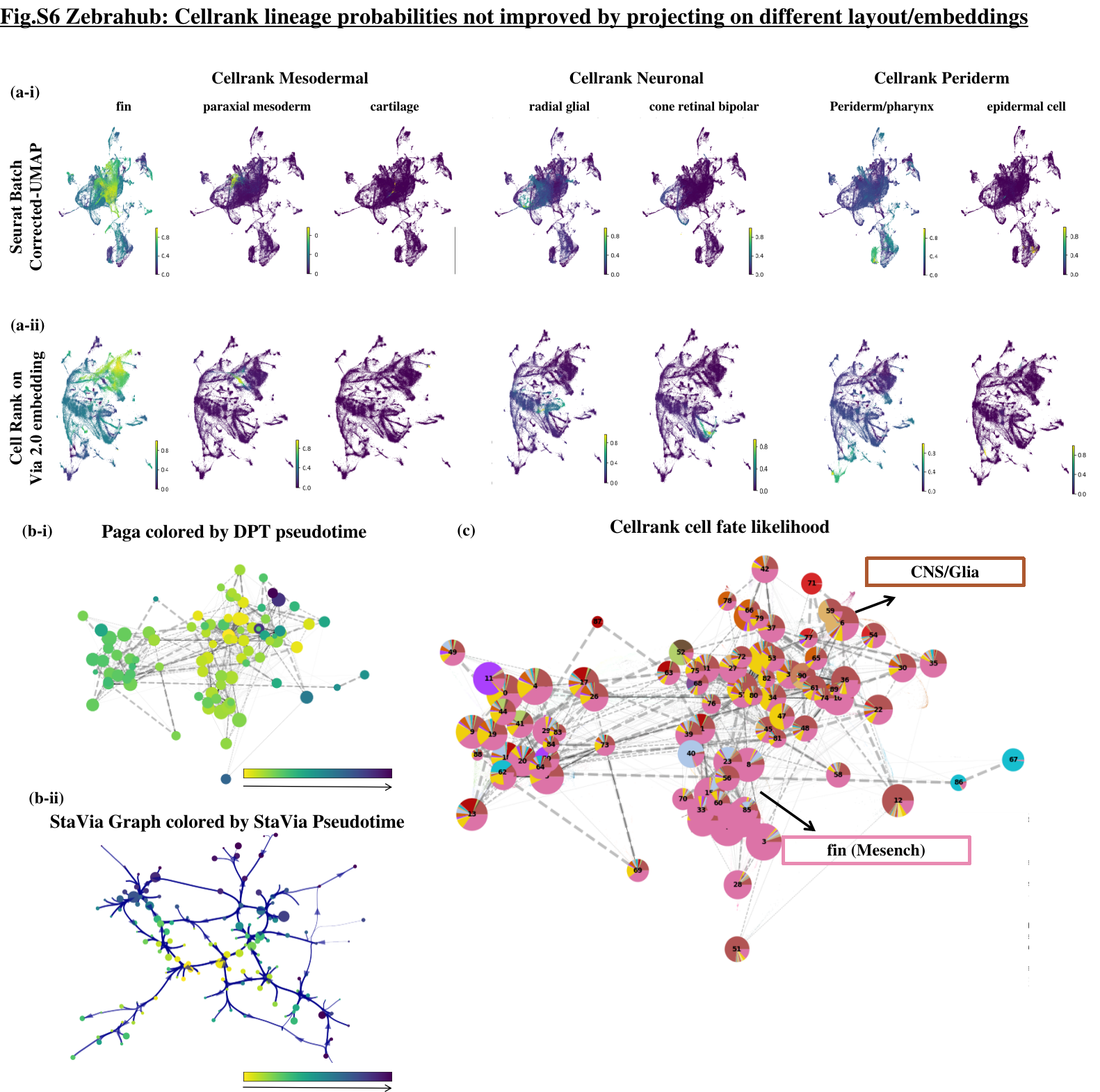
**

***Fig.S6 Zebrahub: CellRank lineage probabilities does not improve by projecting on different layout/embeddings*** *(a) Automatically captured cell fates for CellRank are shown here. The lack of end-to-end information in CellRank lineage probabilities cannot be corrected by simply using a different layout for the single-cell embedding, we plot CellRank’s lineage probabilities for each of its automatically detected cell fates on the UMAP on batch corrected PCs provided in the publicly available data file for Zebrahub (Anndata file without RNA velocity) as well as on StaVia’s Atlas sc-embedding (b) Paga cluster graph colored by DPT pseudotime shows how the pseudotime scale is distorted. (c) Cluster graph composition is colored by cells’ lineage probabilities towards one of the detected terminal states. Due to the highly diffused lineage probabilities shown in (a) for the fin lineage, we see that the “pink” fin terminal state is overrepresented in the CellRank-paga cell fate cluster graph. It incorrectly suggests that all cells are moving primarily towards the pink fin cell fate or the brick-brown Glia*

**
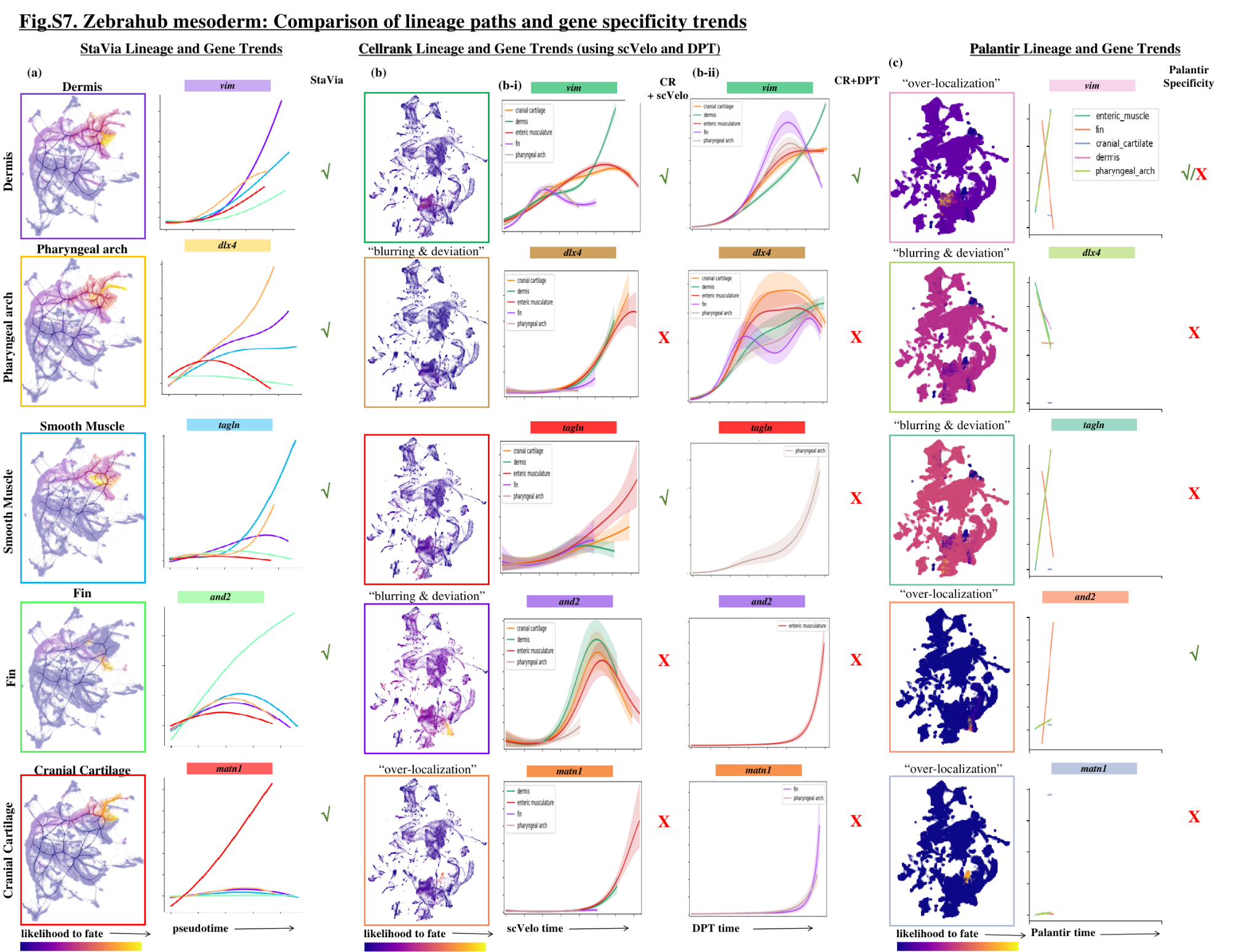
**

***Fig.S7. Zebrahub mesoderm: Comparison of lineage paths and gene specificity trends*** *(a) End-to-end lineage probabilities for automatically detected mesodermal cell fates in StaVia at Memory =10. All mesodermal-lineage gene trends are plotted, but only the lineage for which the marker gene is relevant, is upregulated in each case - indicated by the green tick mark which means the color of the trendline matches the color of the associated gene name. (b) In order to facilitate comparison,* ***we manually assign cell fates for CellRank which does not detect all relevant fates.*** *CellRank’s pathways projected on the UMAP used in the Zebrahub paper and made publicly available (Anndata object with velocity) (b-i) shows the gene trends for the same mesodermal lineages (we manually assign those that are not detected by CellRank in order to compare all cell fate pathways) when CellRank’s lineage probabilities are used together with scVelo’s latent time. (b-ii) Gene trends for CellRank when using the DPT pseudotime (c) Similar analysis for Palantir which required manual setting of cell fates*

**
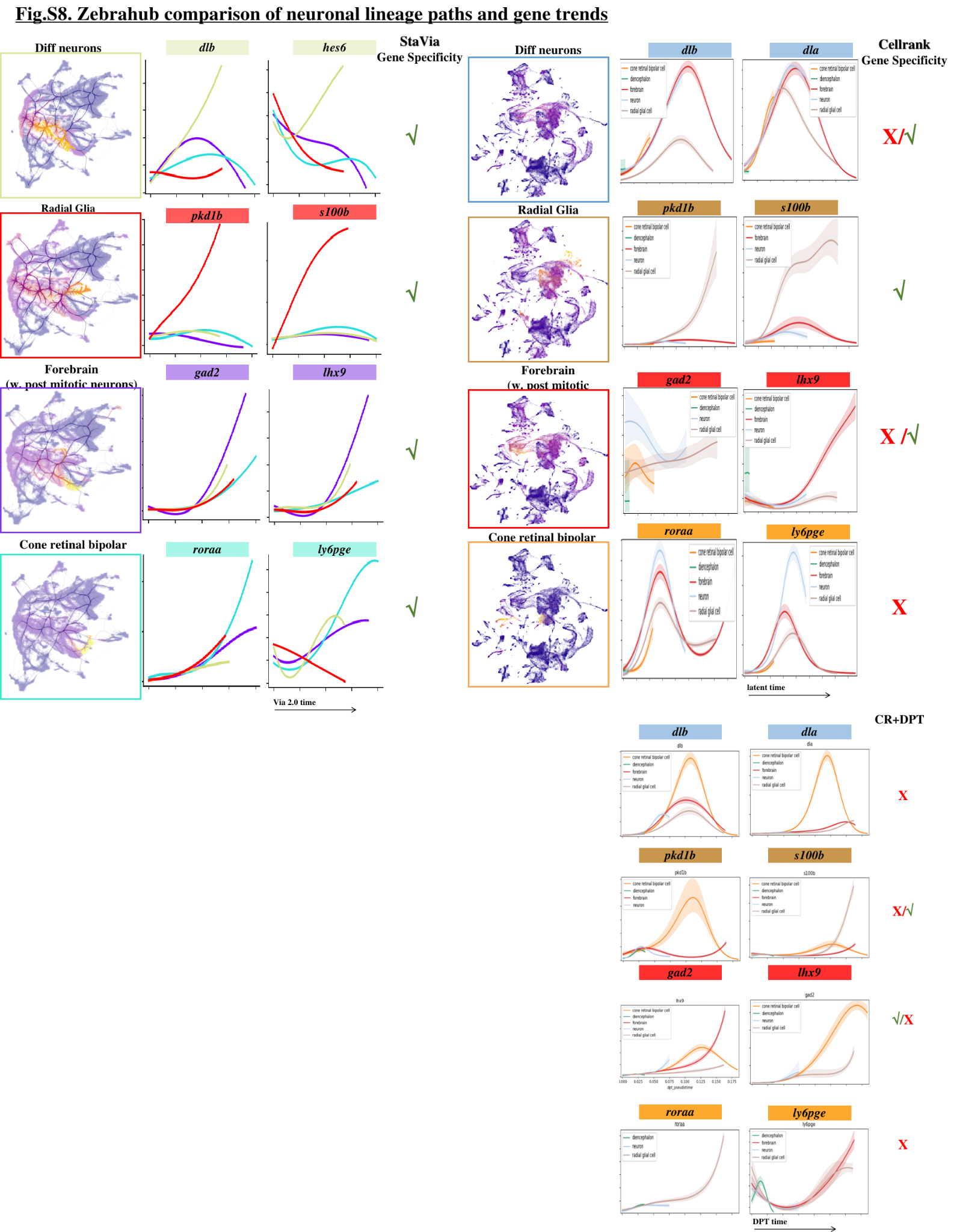
*Fig.S8. Zebrahub neural ectoderm: Comparison of lineage paths and gene specificity trends.*** *Same as Fig.S7 but for the neural ectoderm lineages. Cell fates are manually assigned to CellRank in the case of forebrain and differentiation neurons.*


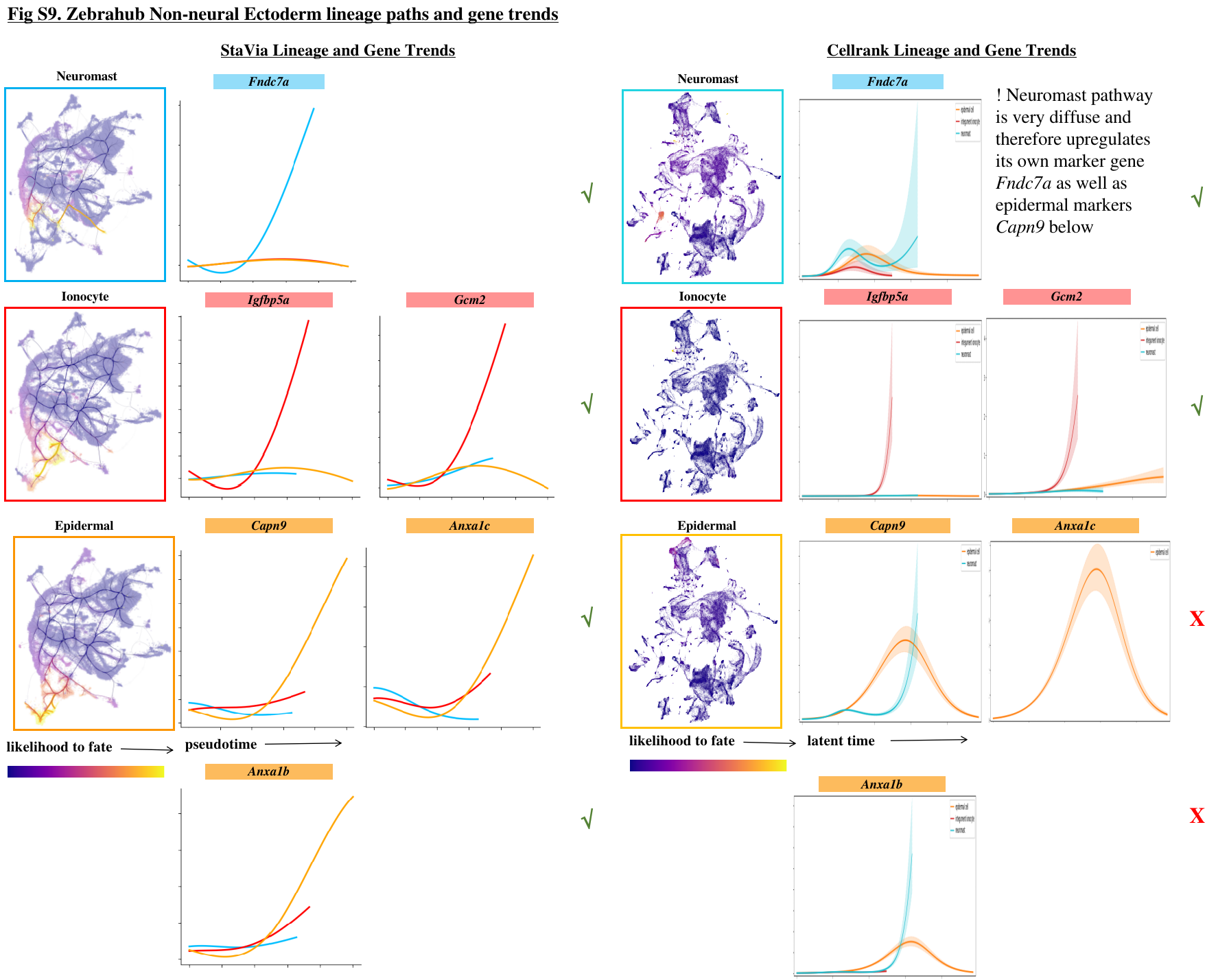


***Fig.S9. Zebrahub non-neural ectoderm: Comparison of lineage paths and gene specificity trends.*** *Same as Fig.S8 but for the non-neural ectoderm lineages. Cell fates for Ionocyte and Neuromasts are manually assigned to Cellrank as they are not automatically detected.*

**
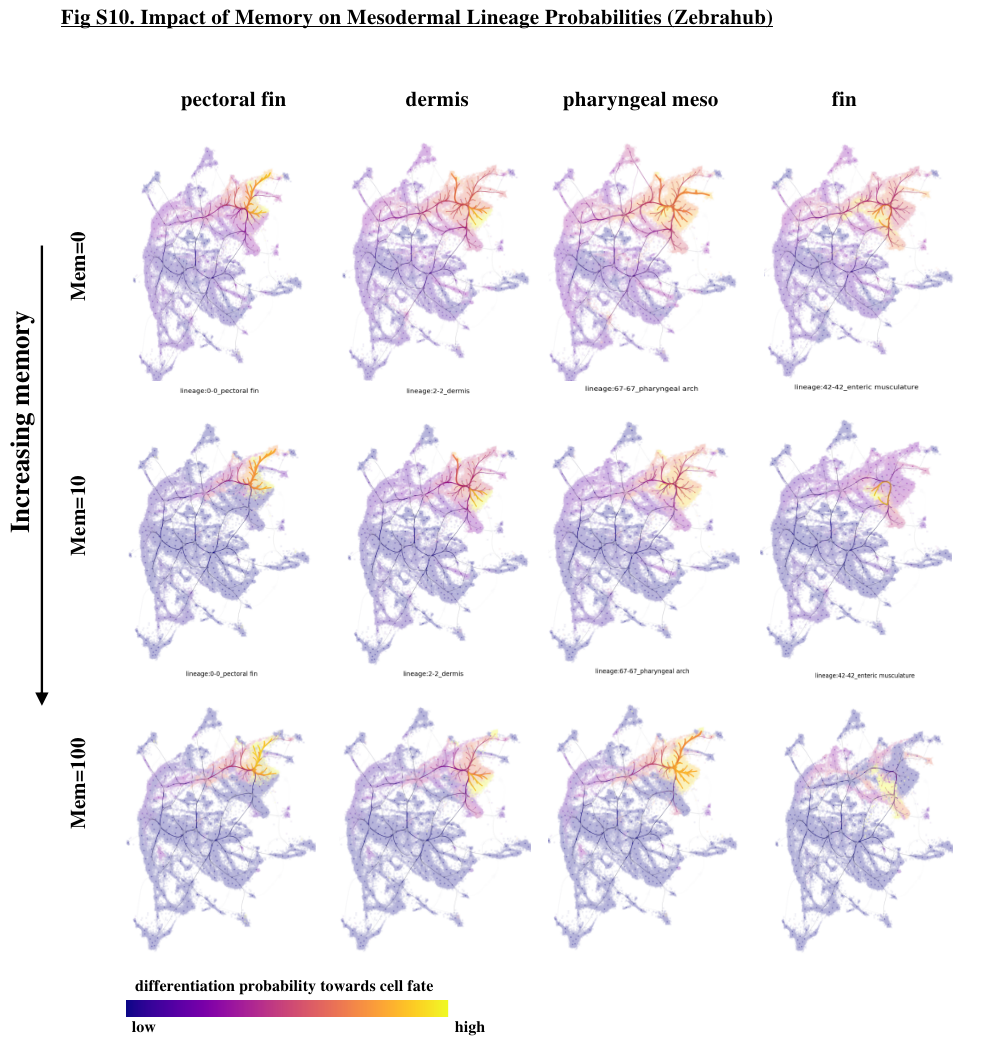
*Fig S10. Impact of Memory on Mesodermal Lineage Probabilities:*** *When the random walk has no memory (is first order), we see that the pharyngeal and fin lineages have significant cell-fate probabilities in the non-mesodermal middle and lower branches, indicated by the light purple-pink coloration in these lower branches. As memory is increased the middle and lower ectoderm branch become a darker blue and the cells residing on them do not express a likelihood towards the mesodermal lineages. Including random walks with memory therefore helps refine the end-to-end pathways to cell fates*


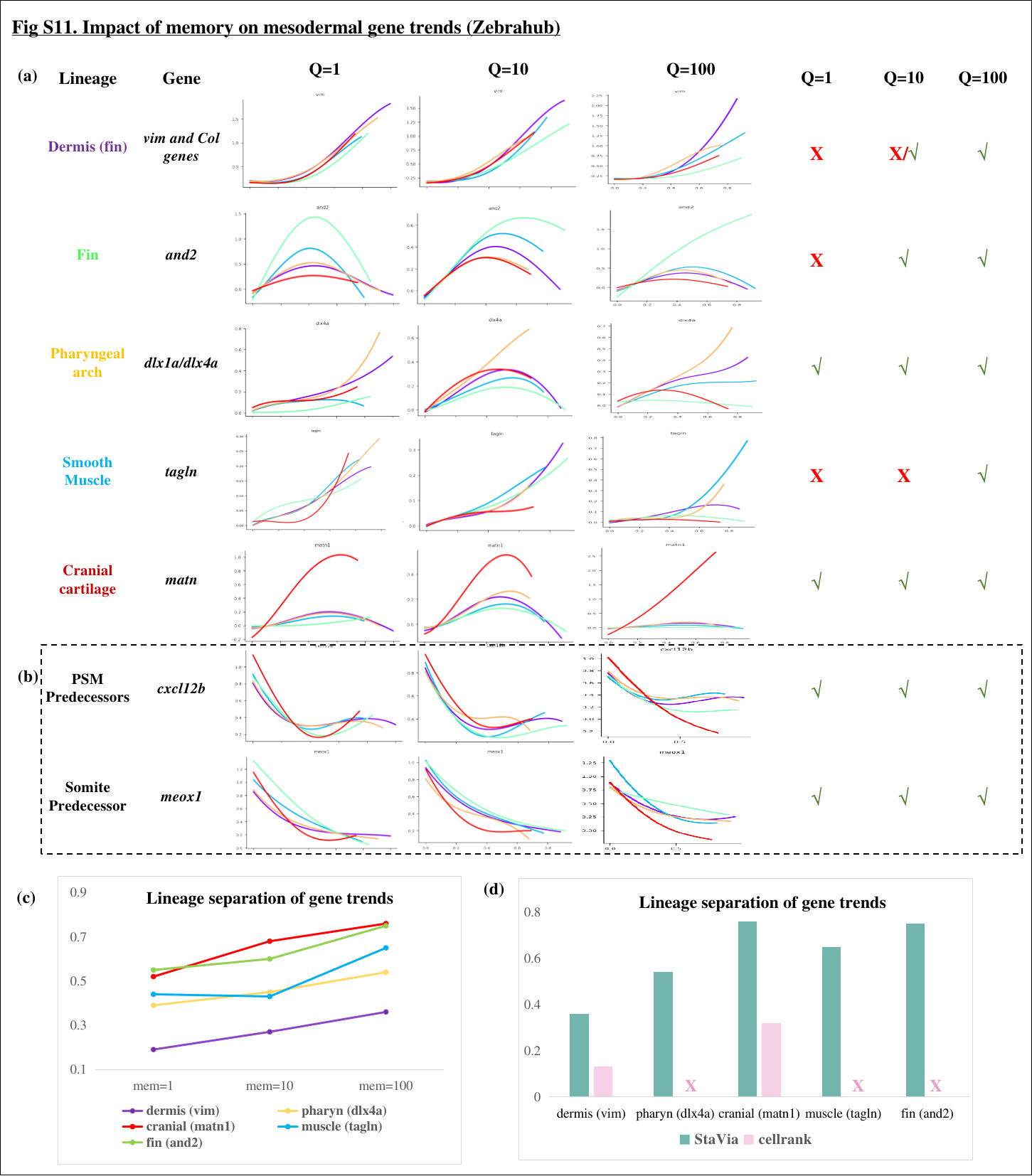


***Fig S11. Impact of Memory on Mesodermal Gene Trends (Zebrahub):*** *(a) When the random walk has no memory (is first order), we see that the pharyngeal and fin lineages have significant cell-fate probabilities in the non-mesodermal middle and lower branches, indicated by the light purple-pink coloration in these lower branches. As memory is increased the middle and lower ectoderm branch become a darker blue and the cells residing on them do not express a likelihood towards the mesodermal lineages. Including random walks with memory therefore helps refine the end-to-end pathways to cell fates. (b) The predecessor genes are correctly shown to all be downregulated along the pseudotime axis. (c) the graph shows the lineage separation in StaVia at no memory (denoted by mem=1) and higher levels of memory (mem=10, 100). As memory increases the separation for all 5 mesodermal lineages is more pronounced. The separation is measured as the ratio of the average area between the gene trend whose lineage is associated with that gene and other lineage trends for that gene, versus the area under the lineage-of-interest’s gene trend. (d) The StaVia lineage separation compared to CellRank’s lineage separation. The ‘X’ marks instances where the incorrect lineage is most upregulated as seen in Fig.S7b where CellRank’s trends are plotted. Palantir’s trends are not quantified as the gene trends are very distorted (as seen Fig.S7c)*

**
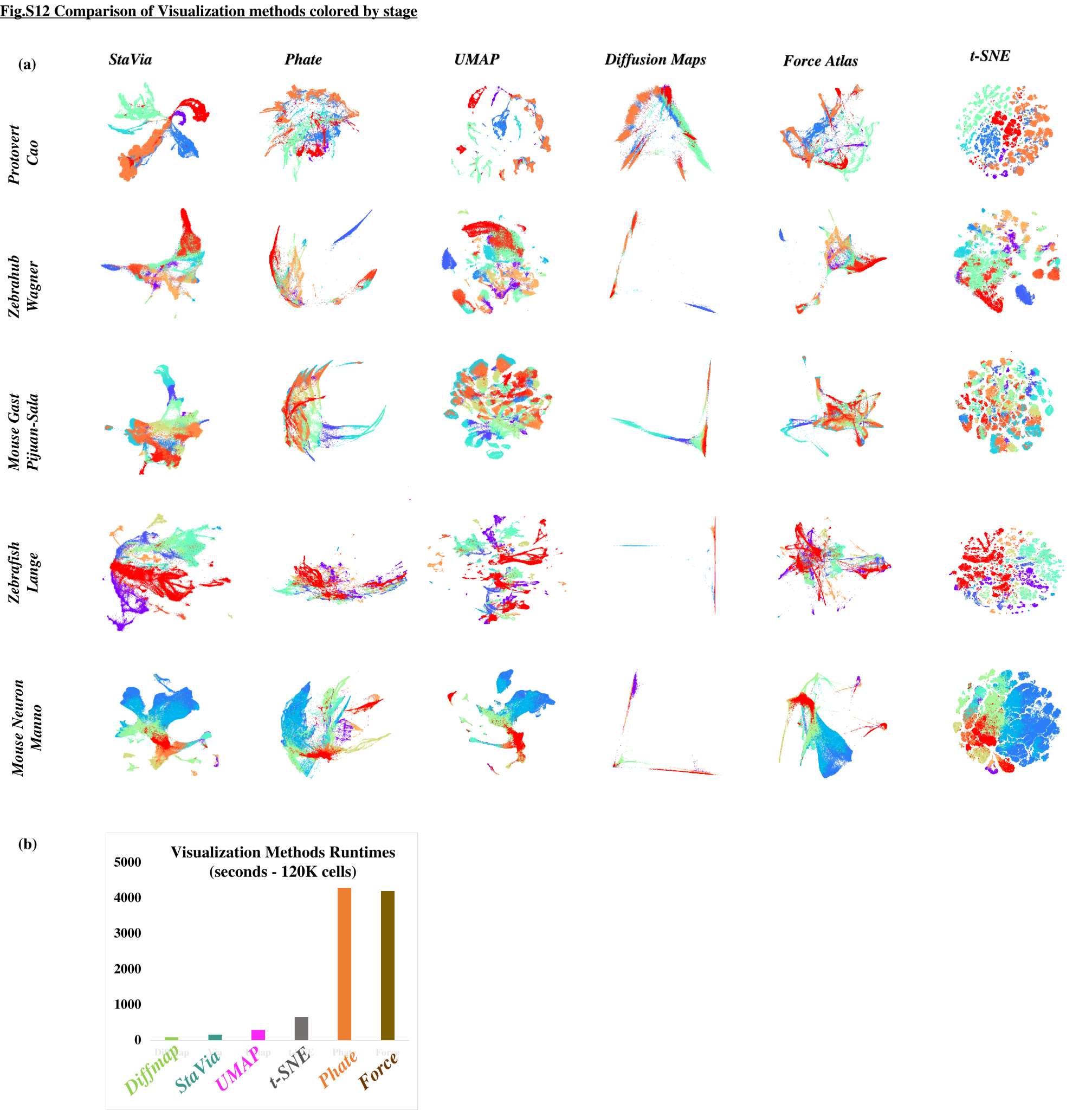
**

***Fig.S12 Comparison of visualization methods on different time-series RNA-seq datasets.*** *(a)* *Colored by known tissue type. (b) Comparison of runtime in seconds to compute the single cell embeddings for Zebrahub (120K cells). We note that the 8 Million Mouse Pup dataset took about 3 hours of TI computation and an additional 13 hours for Atlas creation in StaVia. The visualization took 18 hours in UMAP. RAM usage is peak when loading the h5ad gene-expression file, requiring >500GB. By saving a data file with only the PCs, the RAM requirements can be reduced.*


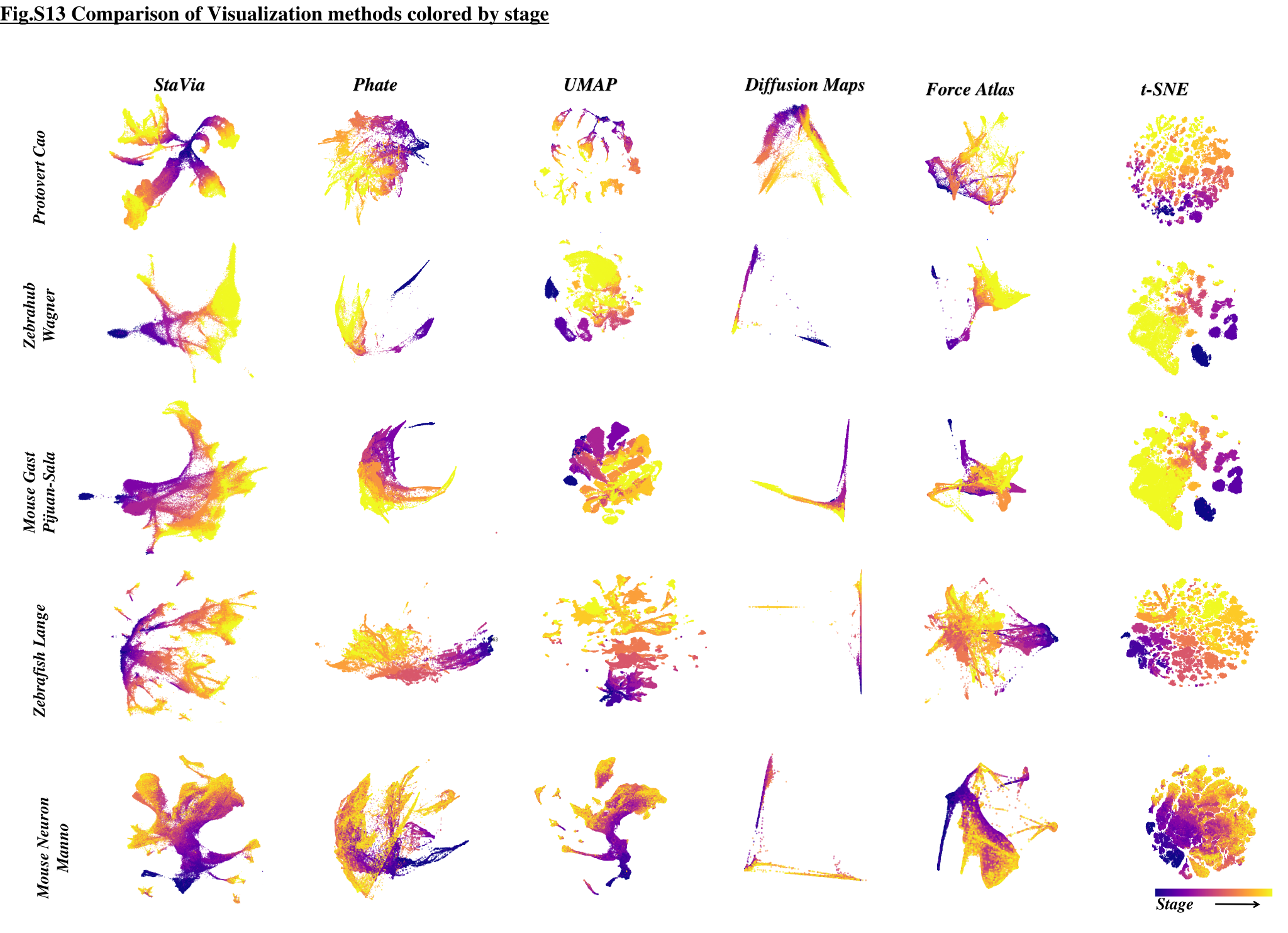


***Fig.S13 Comparison of visualization methods on different time-series RNA-seq datasets.*** *Colored by known developmental stage.*


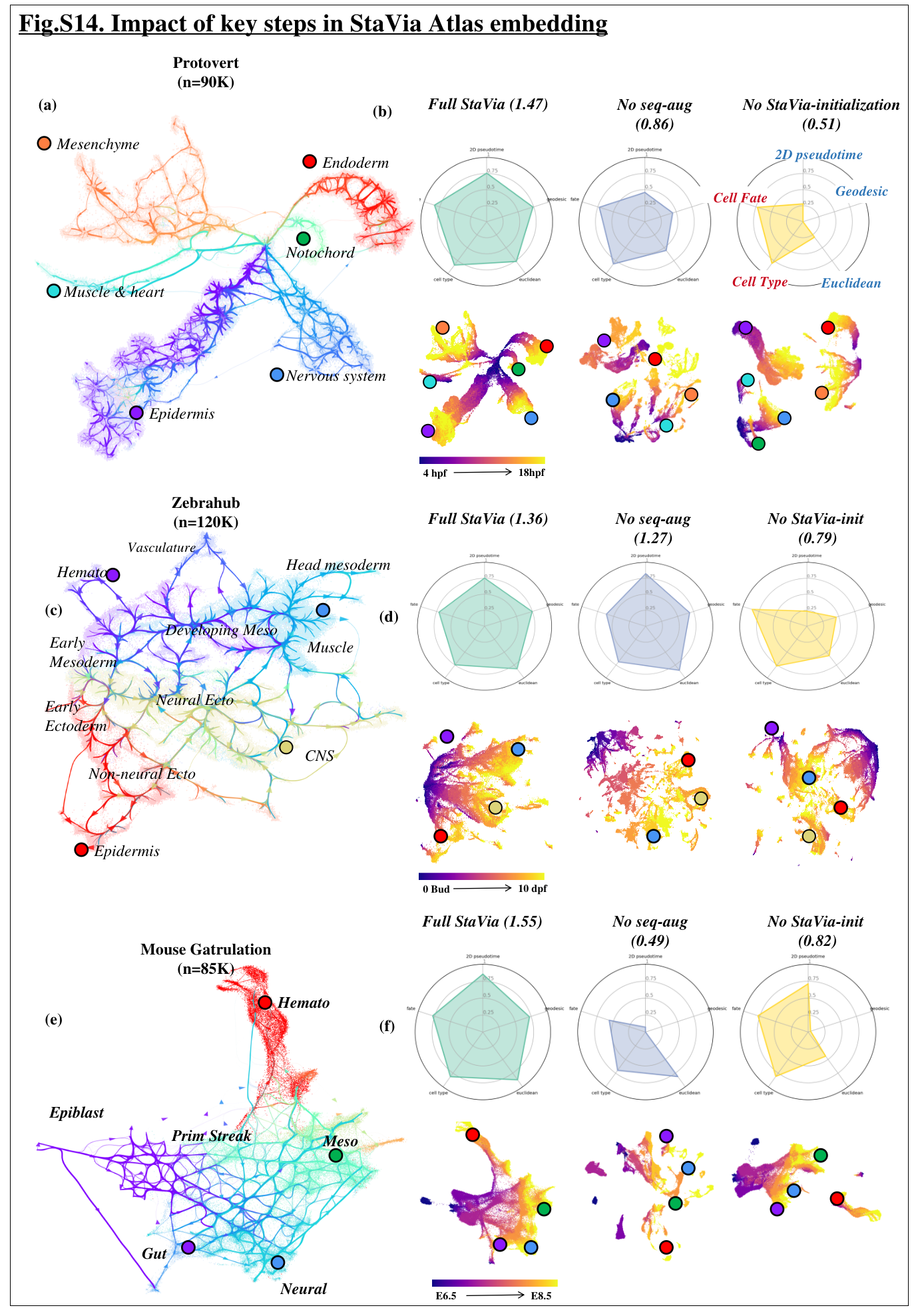


***Fig.S14. Impact of key steps in StaVia Atlas embedding*** *(a) StaVia Atlas view colored by cell type for Ascidian embryo (b) showing the impact on quantitative score and quality of visual embedding colored by stage, when knocking-out one at a time, one of the main steps in generating the embedding. “Full” is the embedding score with all steps intact. “No-seq-aug” does not augment the sc-KNN graph with the known time-series information. “No Via-Initialization” skips the step where the embedding layout is otherwise initialized using the force-directed layout of the forward-biased TI cluster graph. (c-d) Zebrahub (e-f) Mouse Gastrulation*

**
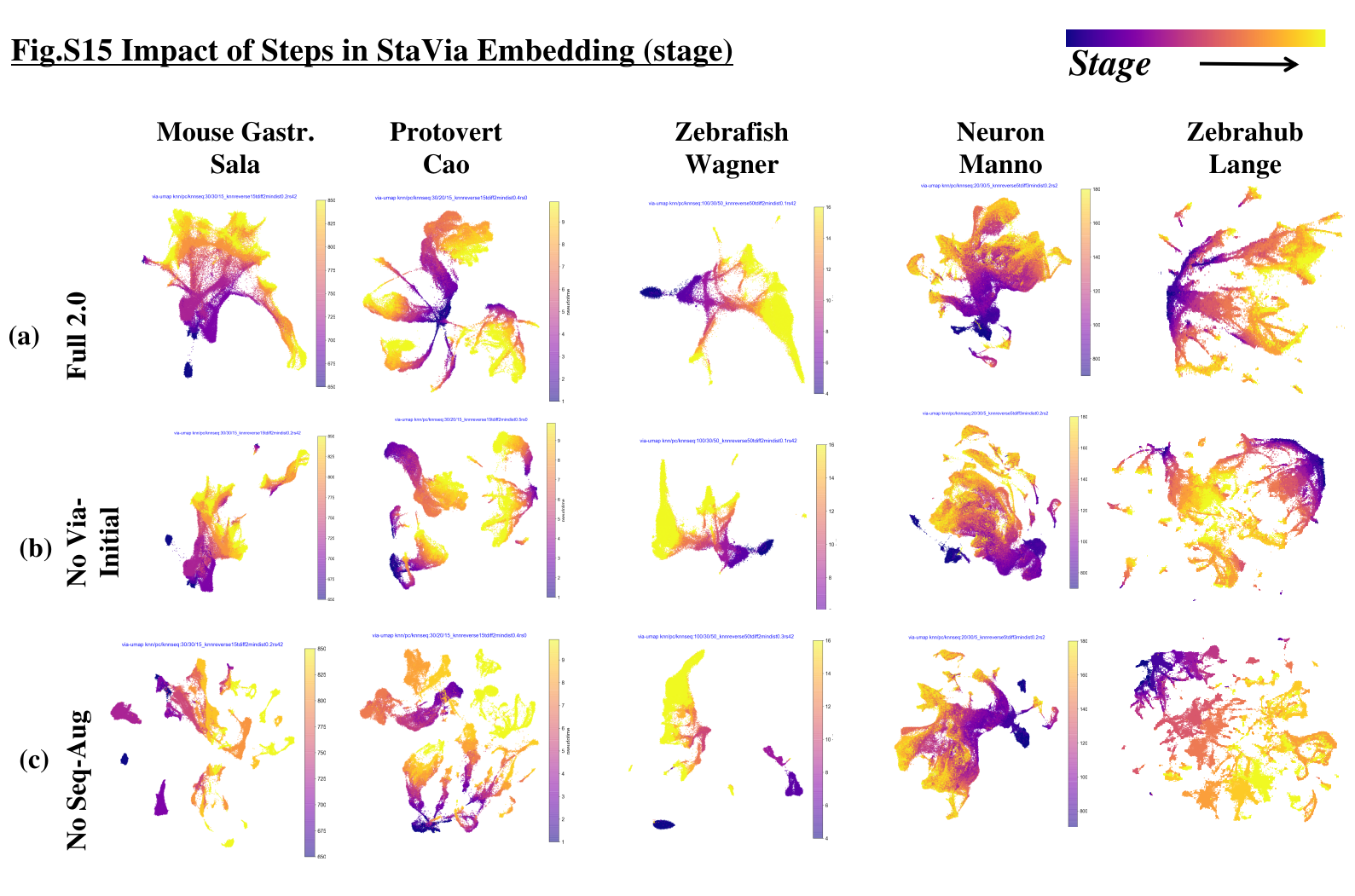
**

***Fig.S15 Impact of Steps in StaVia Embedding (cells colored by known developmental stage)*** *Each row represents the effect of removing one of the key steps in the embedding computation. (a) Includes all key steps (b) Skips via-initialization of embedding using TI-directed and weighted cluster-graph layout (c) Skips leveraging experimental sequential labels to sequentially augment sc-KNN graph*

**
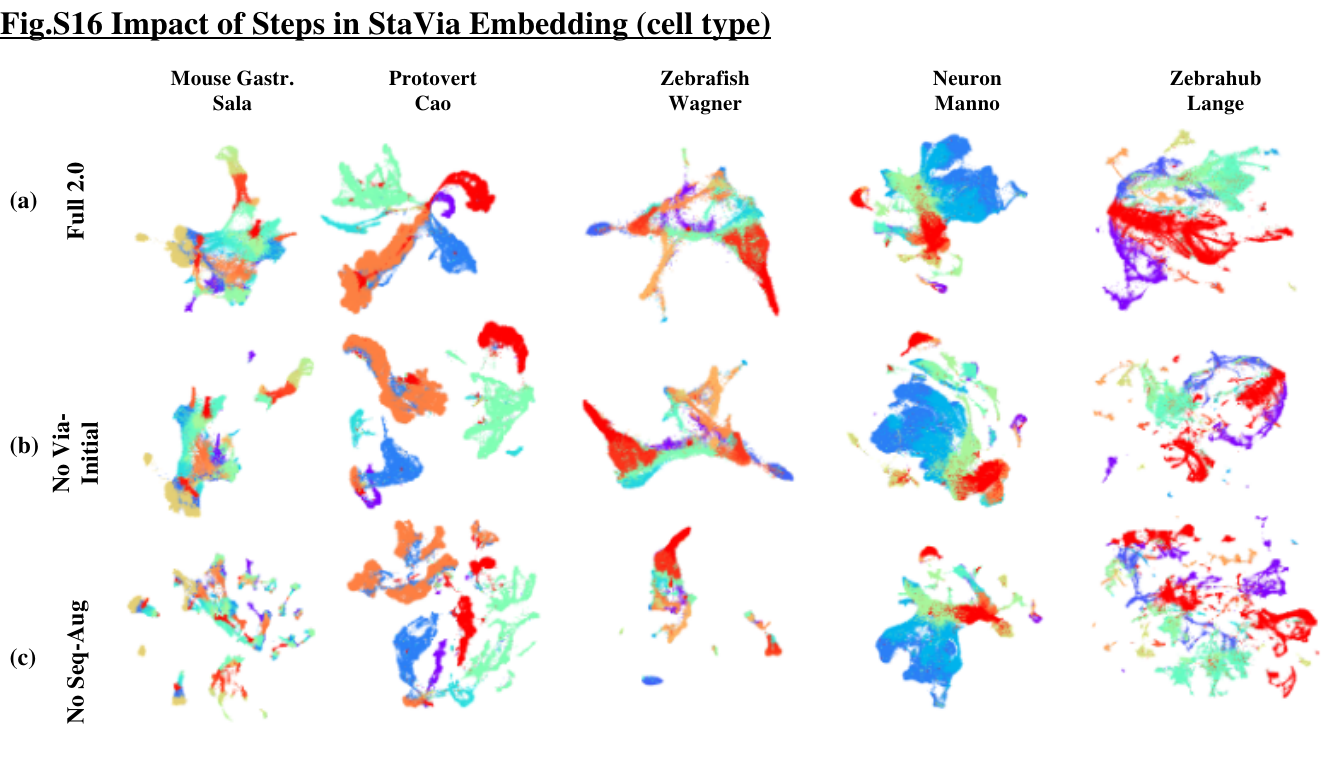
**

***Fig.S16 Impact of Steps in StaVia Embedding (cells colored by tissue type)*** *Each row represents the effect of removing one of the key steps in the embedding computation (same as Fig.S15)*

**
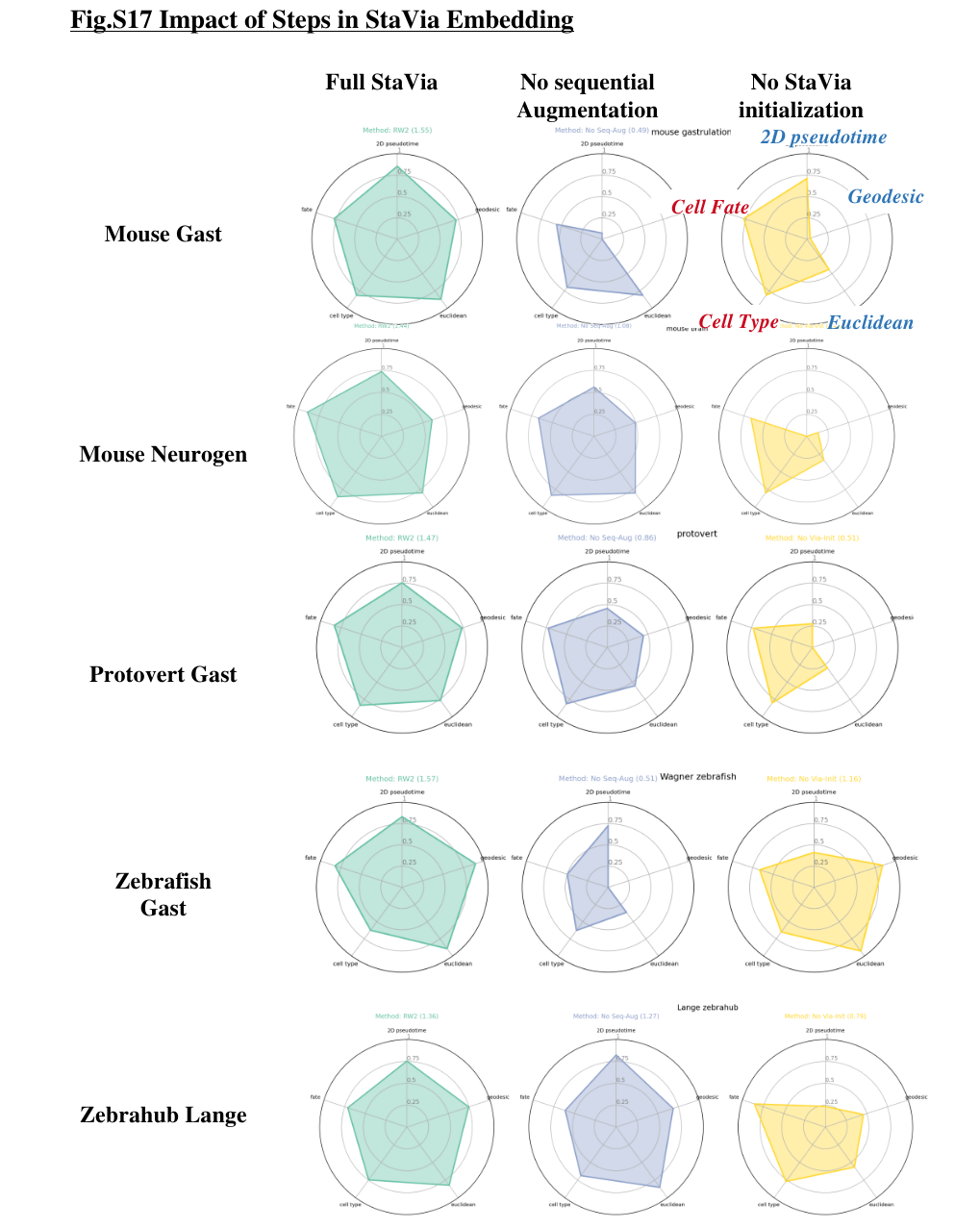
**

***Fig.S17 Impact of Steps in StaVia Embedding on different time-series RNA-seq datasets.*** *Radar plots scoring preservation of structural continuity (blue metrics: 2D pseudotime, Geodesic root-cell correlation with developmental stage, Euclidean root-cell distance correlation with developmental stage) and cell type separation (red metrics: Cell Fate, Cell Type).* *Each column represents the effect of removing one of the key steps in the embedding computation. (Column 1) Includes all key steps (Column 2) Skips via-initialization of embedding using TI-directed and weighted cluster-graph layout (Column 3) Skips leveraging experimental sequential labels to sequentially augment sc-KNN graph.*

*
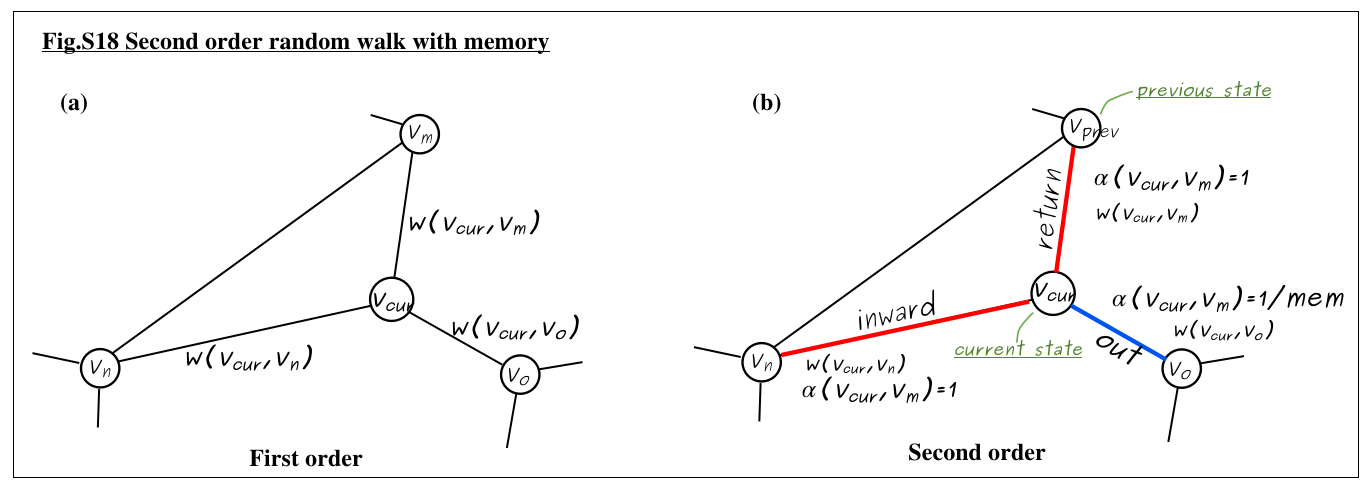
*

***Fig.S18 Formulation of Second order random walks with memory for StaVia***

***
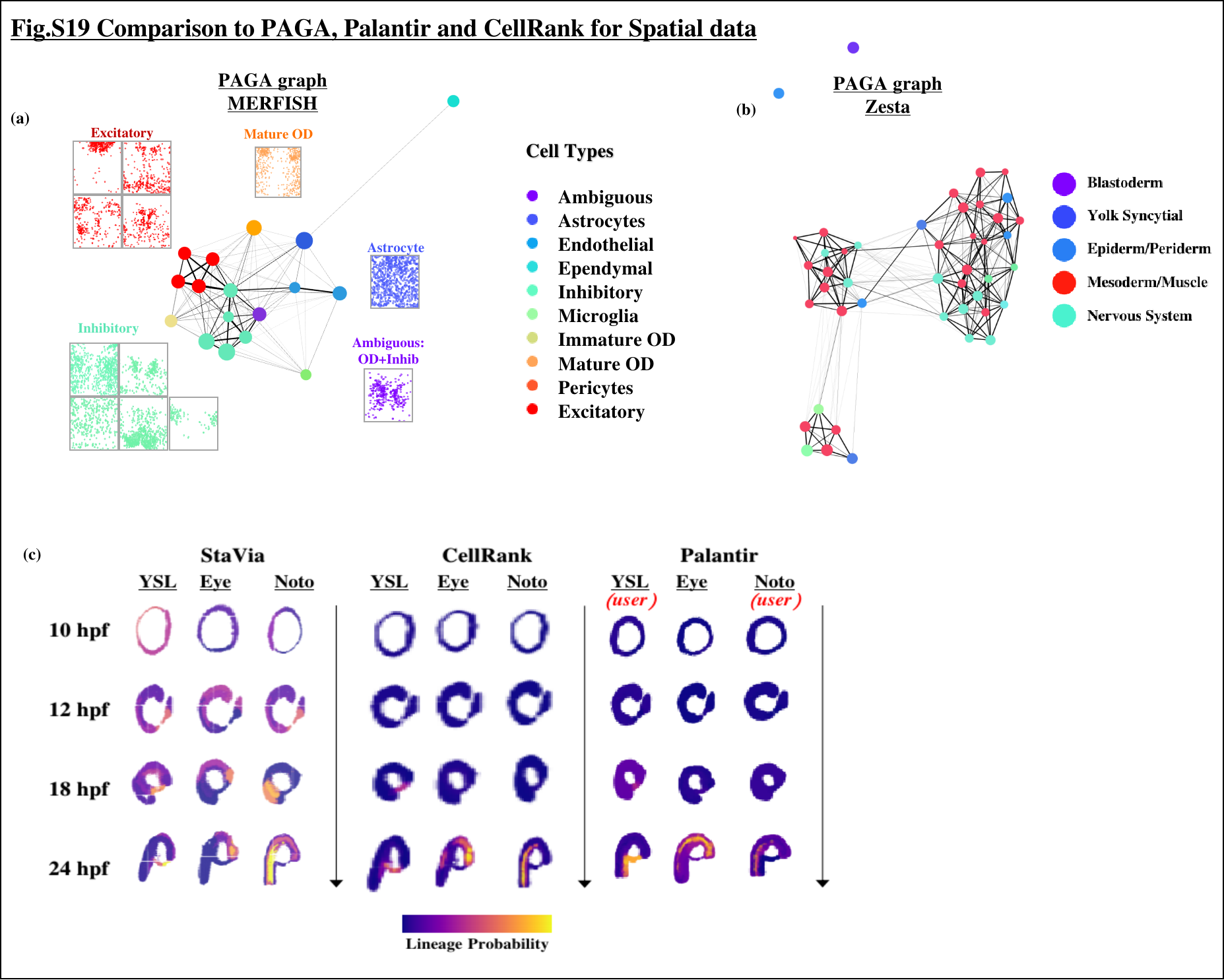
Fig.S19 Comparison to PAGA, Palantir and CellRank for Spatial data*** *(a) PAGA of the MERFISH cells without integrating spatial information. (b) PAGA graph of ZESTA spatial-temporal Stereo-Seq dataset colored by the major cell type of each cluster. Blastoderm 3hpf (purple) and YSL-5hpf cells (light blue) are entirely separated from the rest of the 10hpf-24hpf cells which comprise the three connected subnetworks (c) the single-cell lineage probabilities for 3 example terminal cell fates in ZESTA predicted by StaVia, CellRank and Palantir. CellRank and Palantir cannot identify any relevant populations in the 10-18hpf time points that contribute towards the final cell fate. We have to manually predefine most of the cell fates in Palantir in order to compare lineage probabilities.*

***
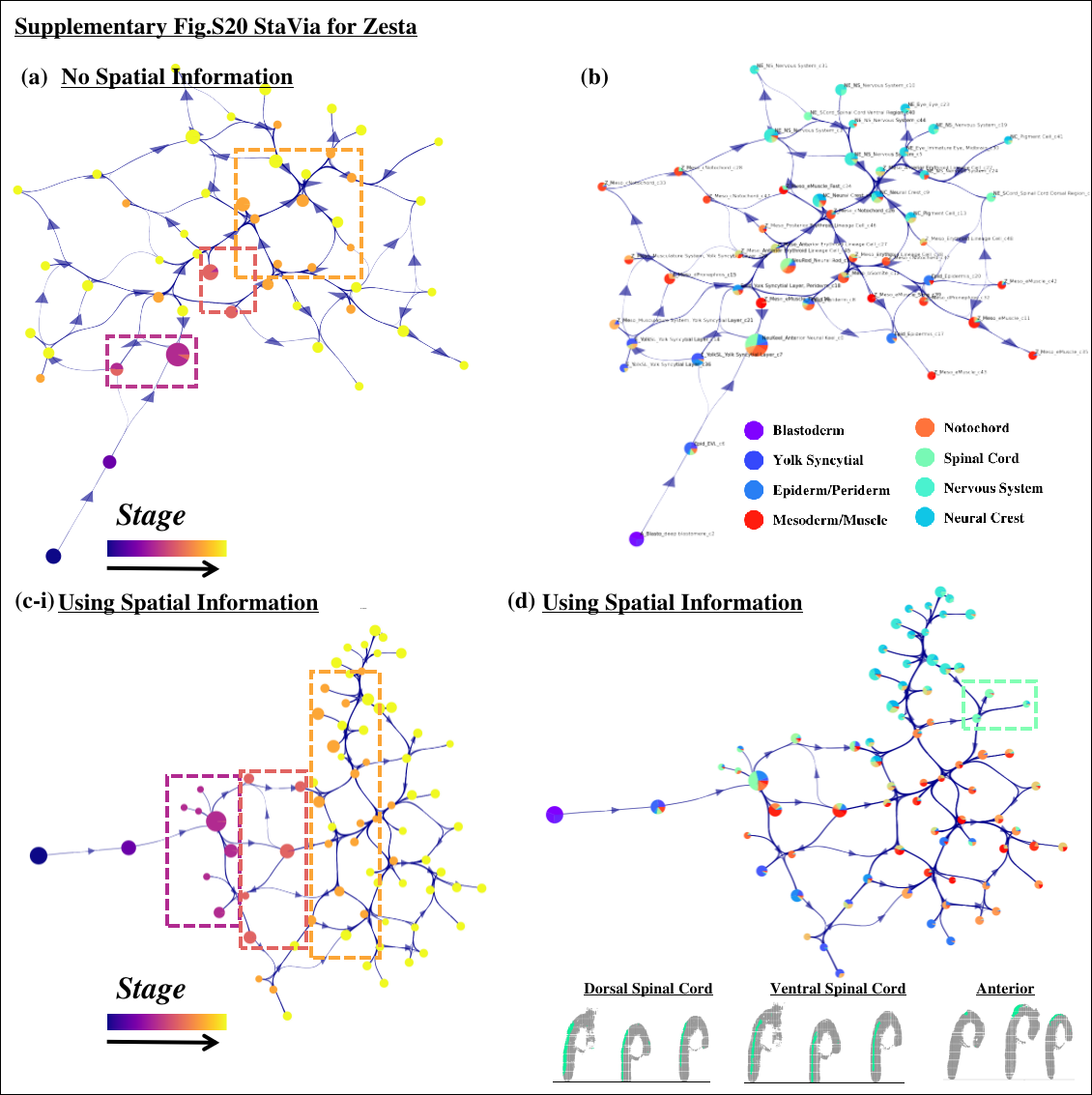
***

***Fig.S20 StaVia for Zesta - with and without spatial coordinates*** *(a) StaVia without spatial information resolves fewer early stage cells into cell type specific clusters. There are for example only 2 magenta 8hpf clusters which combine the early neural keel and segmental plate and the yolk syncytial layer. In (b), these three cell types are separated into 3 main clusters.*
