# Supplementary material for: StaVia: spatially and temporally aware cartography with higher-order random walks for cell atlases
Source: Genome Biol. 2024 Aug 16;25:224. doi: 10.1186/s13059-024-03347-y (PMC11328412; doi:10.1186/s13059-024-03347-y)
Supplement: Supplementary file 2 — Additional file 2. Review history [file 13059_2024_3347_MOESM2_ESM.docx]

**1^st^ round**

**Reviewer 1**

=This paper presents an end-to-end trajectory inference framework that can uncover cellular trajectories/pathways for large-scale single-cell atlases. The method is mainly based on their previous works, and further integrates with higher-order random walks. By analyzing a murine gastrulation atlas and a zebrafish developmental atlas, the authors show the performance of the proposed method VIA 2.0. The considered problem is important. The details of comments are as follows.

1. Clustering plays an important role in the whole method. When the number of cells is very large, there should exist high cluster heterogeneity for different developmental stages or different cells types. How to set the cluster-related parameters such that the clusters along different lineages have comparable developmental pseudotime? Each cluster is taken as one node in the graph, can we get the pseudotime for each cell in the cluster?

2. How to set the edge directions using the scRNA velocity? How to set the user-defined parameter? Any guidance?

3. When using the proposed method, more complex paths appear between two stages. How to combine these paths to get the whole differentiation flow?

4. The visualization is for the atlas view. Generally, we can see the overall pattern of all cells. Can we get into the details of the trajectory? Or can we set some parameter related to the detail level such that by setting it, we can see different levels of the whole picture?

5. There are many findings using the proposed method. Are there existing works that supporting these findings?

6. There are many parameters when using the proposed method. Please give some criteria or suggestions for choosing these parameters when using it.

7. We cannot find the reference for Via 1.0 in the paper though the current method is based on Via 1.0.

8. I think the codes in the link of the paper is Via rather than the updated Via 2.0. The linked paper in the code website is also for Via 1.0.

9. The resolution of some figures is low, for example Fig 3, Fig 8.

**Reviewer 2**

Trajectory inference (TI) is an important computational problem in the field of single-cell biology and despite the availability of multiple TI methods in literature, there is a need for developing novel improved TI methods particularly for handling large-scale temporal scRNA-seq datasets. To that end, the authors in this manuscript have presented an updated version of their TI algorithm, VIA 1.0. The new algorithm is named VIA 2.0 and the major update in this version is the usage of a higher-order lazy teleporting random walk with memory which can incorporate real time and RNA velocity information along with gene expression for computing the trajectory. In addition, the authors have introduced a new visualization method called cartographic Atlas view that uses kernel density estimation-based edge bundling for highlighting edge connectivities on the single-cell graph. VIA 2.0 is tested on two biological datasets and Atlas view is systematically benchmarked against other visualization methods and is shown to perform better than other visualization methods. Overall, the contributions are novel, particularly the atlas view will be helpful in depicting complex trajectories. I, however, have some comments regarding the trajectory inference of VIA 2.0

1. Similar to VIA 1.0, the trajectory is modeled as a cluster graph by VIA 2.0 where nodes are PARC-based clusters of single cells. Thus, it seems that the trajectory backbone is formed in the same manner as VIA 1.0. Some recent methods (e.g., MARGARET) have been shown to outperform VIA 1.0 for different trajectory topologies. It is necessary that the authors perform a systematic benchmarking of the trajectory inferred by VIA 2.0 using gold standard real datasets and/or simulated datasets (see https://www.nature.com/articles/s41587-019-0071-9 for details on the datasets as well as evaluation metrics) to understand the improvement of VIA 2.0 over VIA 1.0 in capturing different trajectory topologies. The performance should be compared against other TI methods that uses graph partitioning (e.g., PAGA, MARGARET) as well as recently developed methods such as Monocle 3.

2. In the VIA 2.0 TI graph for mouse gastrulation (Fig. 3a), some edges are observed from hematopoietic lineage towards paraxial mesoderm/mesenchyme. Can the authors describe these edges? Are these supported by previous literature?

3. The usage of memory in the formulation improves the gene trends as compared to other methods such as CellRank. However, these improvements are shown qualitatively. Is it possible to show quantitative improvements using known lineages?

4. The authors should cite relevant TI methods/approaches which also model cell state and cell fate dynamics (e.g., Palantir, MARGARET, FateID).

**Authors’ response**

We thank you for providing valuable feedback towards improving our manuscript. We have considered all the comments very seriously, performed new comprehensive analyses, and substantially revised the manuscript which now has a modified title, "StaVia: Spatially and temporally aware cartography with higher order random walks for cell atlases" that more accurately reflects the key strengths of Via 2.0 (now renamed as StaVia). We believe that we constructively and satisfactorily addressed all of the concerns raised by the reviewers. In our point-by-point response letter, we first echo the reviewers’ comments and then respond to each issue with reasoning and relevant revision.

We have carefully revised the main and supplementary text to ensure that the core claims in this paper are well-supported and clearly communicated. In particular, we have added 4 main areas of new analysis:

A new section in the main manuscript, “Spatio-temporal cartography in StaVia captures relationships between cells across space and time,” is a proof of concept that StaVia (Via 2.0) can be generalized to spatial omics data, simultaneously incorporating both spatial tissue information and temporal information, to predict meaningful biological insights.

A new section in Supplementary Note 1: Parameter guidance for StaVia offers guidance on parameter selection, and a quantitative demonstration of their stability with respect to the predicted TI at visualization.

Addition of Supplementary Tables 3a-3d for literature support for StaVia biological findings with literature citations associated with each key biological observation derived from StaVia.

Extension of benchmarking to additional datasets and methods:

Spatio-temporal atlases benchmarked with Palantir, PAGA and CellRank.

Comparison of gene-trends and lineage probabilities for all cell fates for the mouse and Zebrahub time-series atlases for CellRank, PAGA+scVelo and also Palantir.

Quantitative and qualitative assessment of memory in lineage pathway recovery.

Supplementary Note 3 on the choice of comparative methods that can incorporate time/tissue-location or RNA-velocity information towards the analysis of cell atlases.

We believe StaVia will be useful to the single-cell community in exploring large atlases, noting a high level of interest from researchers on BioRxiv with an Altmetric score of 71 in the weeks following its online presence.

We look forward to hearing from you.

Sincerely,

Shobana Stassen and Kevin Tsia

*Reviewer #1: This paper presents an end-to-end trajectory inference framework that can uncover cellular trajectories/pathways for large-scale single-cell atlases. The method is mainly based on their previous works, and further integrates with higher-order random walks. By analyzing a murine gastrulation atlas and a zebrafish developmental atlas, the authors show the performance of the proposed method VIA 2.0. The considered problem is important. The details of comments are as follows.*

*R1.Q1 Clustering plays an important role in the whole method. When the number of cells is very large, there should exist high cluster heterogeneity for different developmental stages or different cell types. How to set the cluster-related parameters such that the clusters along different lineages have comparable developmental pseudotime? Each cluster is taken as one node in the graph, can we get the pseudotime for each cell in the cluster?*

R1.A1. The pseudotime of clusters (and single cells) calculated by StaVia is relative to a common root cluster (or root cell), thus the more differentiated cells from later developmental stages (regardless of which cell type/lineage type) should still have higher pseudotime than cells from earlier developmental stages since they will be transcriptomically more distant from the root even if the number of clusters varies across main branches of the cluster graph. As such, the pseudotime is a measure of the relative transcriptomic (or feature level) difference between a given cell and the root state. If the dataset has multiple separate (disconnected) components, the pseudotime is relative to the root cell belonging to its respective component.  The spatial and/or temporal information passed to StaVia helps adjust the graph-level connectivity by preventing discontinuities and graph fragmentation that often arises when purely using expression data on very large and heterogeneous data. E.g. earlier time points where there are often fewer cells relative to more differentiated stages frequently appear disconnected to the remainder of the data. By adding edges between temporally adjacent time points (using temporal_knn**,** see the new **Supplementary Note 1** for guidance on this parameter) that are also similar in feature/gene-space and removing links between time points that are very distant (this threshold can be defined by the user as time_diff, and is by default only set to remove edges between very distance time points), the pseudotime and TI computations are better aligned with the underlying biology.

The single-cell level pseudotime and lineage differentiation probabilities in StaVia are calculated as the weighted average of cluster level pseudotimes/differentiation probabilities of a single-cell’s nearest neighbors (scKNN graph) and the cluster membership of these single-cell nearest neighbors.

*R1.Q2. How to set the edge directions using scRNA velocity? How to set the user-defined parameter? Any guidance?*

R1.A2. The scRNA velocity can be used to determine the direction of edges. However, given these readings can be very noisy and at times velocity models are incompatible with the biology [Zheng, S.C et al., 2023, Bergen V. et al., 2021], we allow for the directionality to be set as a weighted average of pseudotime based direction, and scRNA velocity direction by using the parameter velocity_weight. Directionality is added to the original feature-based symmetric/undirected transition matrix  Tundirected by biasing the relative weighting of edgeij (from celli to cellj) versus the edgeedgeji based on pseudotime and RNA velocity.  The final transition matrix is given by:

(weightvelo × Tvelo) +((1-weightvelo) × Tpseudotime).

The velocity based edge-weights between celli and cellj are calculated by using the cosine similarity between celli’s velocity, vi, and the change in gene expression between *cell_j_* and *cell_i_*. The result (ranging from -1 to 1) is then passed through a logistic function and used to reweight the original undirected gene-expression transition matrix. A negative cosine similarity from *cell_i_* and *cell_j_* passed through the logistic function thus weakens the weight of that transition *edge_ij_*, and conversely a positive cosine similarity existing in the opposite direction from *cell_j_* to *cell_i_* increases the weight of *edge_ji_*, resulting in this case a biasing of transition in the *j-to-i* direction. The edge-direction between two cells when using pseudotime considers the difference between the pseudotime values (normalized to range from 0-1) of cells (or clusters) *i and j* and is then similarly passed through a logistic function before reweighting the gene-based transition matrix to create Tpseudotime.

**Supplementary Note 1** now offers the following guidance on using the “velocity_weight” parameter: *The default value when scRNA-velocity is available, is set to* velocityweight=0.5 *such that both pseudotime and scRNA-velocity contribute equally to the edge directionality. If the dataset possesses scRNA-velocity available for a large number of genes, the weight can be increased such that* velocity_weight>=0.5*. Conversely, if the RNA velocity data is thought to be noisy, have transcriptional boosts and incompatible with model assumptions of time-constant rates of splicing/degradation and transcription [Zheng, S.C et al., 2023], then a de-emphasis on the directionality inferred by scRNA-velocity may be suitable provided there is a clear subset of cells which represent an initial state.*

*It should also be noted that when RNA velocity is available, the root can be auto-detected. StaVia proposes 3 root states for the user to consider when defining the start state (if no user-feedback is, the first of these roots is selected, as the cluster with the lowest stationary probability). When prior knowledge is available and preferred, then the root can be user-defined.*

*R1.Q3. When using the proposed method, more complex paths appear between two stages. How to combine these paths to get the whole differentiation flow?*

R1.A3. The differentiation flow (such as those in **Fig.3c** and **Fig.6b** and **Fig.7b**) is a tree-like interpretation of the more complex cluster graph-level abstraction. The pathways shown on the differentiation flow charts are calculated based on the most probable (shortest) paths on the weighted-directed cluster-graph to identify and thus represent a high-level summary of pathways from root to the auto-detected cell fates. The Sankey differentiation flows auto-generated from the cluster graph can be used as a simplified overview (showing only the most likely path for each cell fate) of the more complex cluster graph view rather than an exhaustive view of all possible pathways.

*R1.Q4. The visualization is for the atlas view. Generally, we can see the overall pattern of all cells. Can We get into the details of the trajectory? Or can we set some parameters related to the detail level such that by setting it, we can see different levels of the whole picture?*

R1.A4. The level of edge-fusion and cell-type resolution can be modulated by a few key parameters whose effect we demonstrate in the new **Supplementary Note 1 and accompanying new Supplementary Fig. S0Ba-d**. Briefly, we note that the Atlas View allows visualization at different resolutions of the connectivity (edges) in the trajectory as well as the cell types (number of milestones in the Atlas View). The connectivity visualized in the Atlas View is based on the sc-knn graph constructed in the high-dimensional feature space (and not a graph constructed using the 2D embedding which would otherwise introduce further loss of edge information). This way, the transitions that are illustrated stem from the high-dimensional graph, and are not influenced by the selection of embedding. Increasing the number of milestones in the Atlas View also allows a more granular view of the potential transitions between sub-cell types. The edge bundling helps visually reduce the clutter at even a high number of milestones, by combining dominant pathways emerging in these edges.

*R1.Q5. There are many findings using the proposed method. Are there existing works that support these findings?*

R1.A5. The Atlas View and StaVia graphs offer many biological findings for each of the developmental processes investigated. StaVia stands out from other methods by uncovering these insights without manual subsetting of the data, but retaining a holistic view of the entire duration and organism. To more clearly communicate to the reader the existing literature which supports our findings we have made the following revisions:

1) Ensured that a citation directly follows each biological finding within the text.

2) Moved some of the discussion which elaborates on the findings into a section in S**upplementary Note 2 (“StaVia reveals 3 developmental patterns in Murine Embryogenesis”)**  which allows us to offer more supporting context, while keeping the main text digestible and focused on the method.

3) Added **Supplementary Tables 3a-3d** for each dataset, to list each finding, the supporting figure and relevant citation.

**Table S3a. Mouse Gastrulation**

**Table S3b. Zebrahub**

**Table S3c. MERFISH Preoptic Mouse**

**Table S3d. Stereo-seq Zesta Zebrafish STOmics**

*R1.Q6. There are many parameters when using the proposed method. Please give some criteria or suggestions for choosing these parameters when using it.*

R1.A6. This is a very useful suggestion and we have now added a supplementary section on parameter guidance under **Supplementary Note 1** and updated StaVia’s ReadTheDocs pages to help inform the user on parameter selection and impact. The inspection of parameters is done both quantitatively - through correlation analysis to assure the user of stable behaviour, and also done qualitatively - through visualized outputs on incremental changes to parameters which we believe will be useful for potential users.

**Supplementary Note 1: Parameter Guidance for StaVia**

This section covers the main parameters influencing TI and visualization in StaVia and can be used in conjunction with the tutorials available on StaVia’s ReadTheDocs pages to help inform the user on parameter selection and impact. We inspect parameters both quantitatively - through correlation analysis to assure the user of stable behaviour, and also qualitatively - through visualized outputs on incremental changes to parameters - which we believe is intuitive and useful for potential users.

**Spatial and Temporal metadata related parameters:**

There are two main temporal parameters that impact how the temporal information is incorporated into the TI and resulting visualizations. These are the knn_temporal (number of sequentially adjacent neighbors added between cells) and time_diff (edges > time_diff apart are removed from the single-cell graph)*.* The main spatial parameter is knn_spatial.

- **Knn-temporal: Supplementary Fig.S0Aa** shows how the interpretation of the trajectory alters based on the number of knn_temporal. We illustrate this using the Atlas View which is derived from both the underlying single-cell and cluster-graph configurations. Adding knn-temporal refines the sequential connectivity, with the marginal change for this dataset stabilizing after knn_temporal >10.   **Supplementary Fig.S0Ab** shows that the inferred pseudotime is very stable predominantly only showing a noticeable change when moving from no time-series information towards using the time-series labels to guide the TI.
- **Time_diff:** This parameter causes fairly subtle changes as the time_diff threshold is only applied as long as a cell continues to maintain more than 5 edges. A time_diff = means that edges between cells that are more than = discrete average time intervals apart will be removed. In **Supplementary Fig.S0Ab,** For τ<5, the hematopoietic branches maintain connectivity to the early mesoderm as edges within these time-intervals represent a greater share of the total edges present. This connectivity between hemato-early mesoderm is lost for greater values of .
- **Knn-spatial:** In datasets with spatial coordinates, we use the knn_spatial parameter in two distinct steps of the StaVia TI computations. StaVia's graph construction leverages a recent concept [V. Singhal 2024] to recalibrate gene expression by considering a cell's environment of its knn_spatial tissues-location based neighbors. Furthermore, StaVia also augments the gene-expression based KNN graph with knn_spatial spatial neighbors when establishing cluster connectivity. **Supplementary Fig.S0Ae** shows how the number of knn_spatial spatial neighbors impacts the ability to delineate cell types at early stages of development, which aids in the construction of pathways towards cell fates.

**Memory:** The use of memory through second-order random walks is a key aspect of StaVia and there are several analyses presented in the main and supplementary sections to highlight the usefulness of this approach. Here we summarize the key figures used to show the impact of increasing levels of memory on recovering lineage pathways and gene trends for all cell fates in two large and complex datasets. Generally speaking, higher memory increases the specificity of lineage pathways towards cell fates and prevents the inclusion or unrelated intermediate states. However, very large values can potentially be too restrictive. **Fig.4a** in the main text shows the impact on a subset of recovered pathways, and **Fig.4b** shows the consequential impact on gene trends which use these lineage probabilities to predict the gene trend associated with each lineage along pseudotime. **Supplementary Fig4. and Supplementary Fig.10-11** present these results more exhaustively for all lineages for both Zebrahub and Mouse gastrulation. **Supplementary Fig.5a** quantifies the stability when tuning the memory parameter. **Supplementary Fig.S5b** offers a heuristic for quantitatively identifying a range of memory values, however this works when temporal data is available.

**Edge directions using scRNA velocity:** The scRNA velocity can be used to determine the direction of edges. However, given these readings can be very noisy and at times velocity models are incompatible with the biology [Zheng, S.C et al., 2023, Bergen V. et al., 2021], we allow for the directionality to be set as a weighted average of pseudotime based direction, and scRNA velocity direction by using the parameter velocity_weight. Directionality is added to the original feature-based symmetric/undirected transition matrix  Tundirected by biasing the relative weighting of edgeij (from celli to cellj) versus the edgeedgeji based on pseudotime and RNA velocity.  The final transition matrix is given by:

(weightvelo × Tvelo) +((1-weightvelo) × Tpseudotime).

The default value when scRNA-velocity is available, is set to velocity_weight = 0.5 such that both pseudotime and scRNA-velocity contribute equally to the edge directionality. If the dataset possesses scRNA-velocity available for a large number of genes, the weight can be increased such that velocity_weight ≥0.5. Conversely, if the RNA velocity data is thought to be noisy, have transcriptional boosts and incompatible with model assumptions of time-constant rates of splicing/degradation and transcription [Zheng, S.C et al., 2023], then a de-emphasis on the directionality inferred by scRNA-velocity may be suitable provided there is a clear subset of cells which represent an initial state.

It should also be noted that when RNA velocity is available, the root can be auto-detected. StaVia proposes 3 root states for the user to consider when defining the start state (if no user-feedback is, the first of these roots is selected, as the cluster with the lowest stationary probability). When prior knowledge is available and preferred, then the root can be user-defined.

**Cluster graph resolution and connectivity parameters:** The key parameters which influences the resolution of the clusters-graph is the number of k-nearest neighbors of the single-cell expression graph (knn), the number of features of the input and the level of edge-pruning prior to clustering (edgepruning_clustering_resolution) of the single-cell graph prior to using a graph-based clustering method such as PARC (default). **Supplementary Fig.S0C** uses a scRNA-seq dataset of hematopoiesis (Setty et al, 2019) to show how increasing knn lowers the number of clusters by globally increasing the connectivity between cells thus making them ‘harder’ to separate. By pruning the graph edges using edgepruning_clustering_resolution, we can more locally adjust the overall connectivity of the graph to remove spurious edges at local regions of the graph and resolve a greater number of clusters or sub-types for a given knn without causing graph fragmentation that can otherwise occur for lower global knn values (see PARC Stassen 2020 for a detailed explanation of this parameter).

We note that the temporal parameters (knn_temporal and time_diff) do not influence the number of clusters and are used only to modulate the connectivity of the single-cell and cluster graphs, and done separately from the clustering step. The rationale for this is that temporal and spatial edges are primarily used to examine transitions, and we want to avoid artificially forcing them to cluster together. We refer to figures from Via 1.0 [Stassen 2021] for a detailed analysis on multiple datasets of parameters such as knn, and dimensionality of input data (we have included an overview figure here for completeness).

**Atlas View parameters:** The Atlas View is a key feature of StaVia and the final visualized output can be tuned using a handful of key parameters. It is important to note that altering the visualized resolution of edges and cells does not impact the underlying TI which is influenced by the other parameters discussed earlier in this section. The results are presented in **Supplementary Fig.S0Ba-d**

- Global_visual_pruning: increasing this parameter increases the number of edges shown
- Decay: increasing the decay increases the merging of edges
- Initial_bandwidth: increasing the initial bandwidth increases merging of edges.
- N_milestones: increasing n_milestone increases the resolution of edges on the Atlas View

***Fig.S0A Spatial-temporal parameters (Mouse Gastrulation)*** *(a) Adding knn-temporal refines the sequential connectivity, with the marginal change for this dataset stabilizing after knn_temporal >10. (b) inferred pseudotime and lineage probabilities are very stable, predominantly showing a noticeable change when moving from no time-series information towards using the time-series labels to guide the TI. (c)  Varying time_diff causes subtle changes in the topology. In this example, for time_diff  < 5, the hematopoietic branches maintain connectivity to the early mesoderm as edges within these time-intervals represent a greater share of the total edges present. This connectivity between hemato-early mesoderm is lost for greater values of time_diff. (d) Pseudotime correlation is very high across this parameter. (e) shows how adding knn_spatial spatial neighbors improves the ability to delineate cell types at early stages of development, which aids in the construction of pathways towards cell fates. The dark-pink and burnt-orange-colored boxes house a greater number of early stage clusters as spatial neighbors are accounted for in the cluster graph. Without spatial neighbors (knn_spatial =0), all early-stage cells are placed in a few common large clusters, with no cell type distinction.*

***Fig.S0B Atlas View Visualization Parameters (Zebrahub)*** *Shows how four key parameters impact the visualized resolution of edges. Unless indicated as per the figure, prune_visual_edges = 0.25, initial_bandwidth=0.1, n_milestones =500, decay = 0.4*

***Fig.S0C Cluster graph resolution parameters.*** *Increasing the number of k-nearest neighbors reduces the number of clusters. In this dataset we do not observe a noticeable increase in the number of edges as knn is increased which suggests that connectivity is expressed predominantly within cell types and less between different cell types (the graph is highly modular). Increasing prune_clustering_resolution, retains more edges and thus lowers the number of clusters found in the graph partitioning.*

*R1.Q7. We cannot find the reference for Via 1.0 in the paper though the method is based on Via 1.0.*

R1.A7. We thank the reviewer for pointing this out, it has been added to the reference list.

*R1.Q8. I think the code in the link of the paper is Via rather than the updated Via 2.0. The linked paper in the code website is also for Via 1.0*.

R1.A8. Many of StaVia’s new functionalities were introduced in recent versions of the pyVIA package, with tutorials continuously updated on ReadTheDocs even though the name of the package was still “Via 1.0”. This allowed us to test the usability of these new features prior to a full aunch. The links to StaVia’s ReadTheDocs and GitHub now represent the full functionality described in this paper. Although the python package is still called pyVIA, all tutorials cover temporal, spatial and memory (higher order RW) -driven TI, as well as the integration of the Atlas View to illustrate computational findings.

*R1.Q9. The resolution of some figures is low, for example Fig 3, Fig 8.*
R1.A9. We have attached these as separate TIF files with higher resolution. We note that they become very compressed in the PDF generation of the manuscript file, but when viewed as stand-alone files, maintain their high quality.

*Reviewer #2: Trajectory inference (TI) is an important computational problem in the field of single-cell biology and despite the availability of multiple TI methods in literature, there is a need for developing novel improved TI methods particularly for handling large-scale temporal scRNA-seq datasets. To that end, the authors in this manuscript have presented an updated version of their TI algorithm, VIA 1.0. The new algorithm is named VIA 2.0 and the major update in this version is the usage of a higher-order lazy teleporting random walk with memory which can incorporate real time and RNA velocity information along with gene expression for computing the trajectory. In addition, the authors have introduced a new visualization method called cartographic Atlas view that uses kernel density estimation-based edge bundling for highlighting edge connectivities on the single-cell graph. VIA 2.0 is tested on two biological datasets and Atlas view is systematically benchmarked against other visualization methods and is shown to perform better than other visualization methods. Overall, the contributions are novel, particularly the atlas view will be helpful in depicting complex trajectories. I, however, have some comments regarding the trajectory inference of VIA 2.0*

We thank the reviewer for the positive comments, particularly regarding the usefulness of the Atlas View in visualizing computed trajectories and understanding single-cell data in the context of differentiation landscapes. We appreciate the reviewer’s comments and have tried to address them to the best of our abilities. We hope that our answers clarify any questions surrounding the method and its use.

*R2.Q1. Similar to VIA 1.0, the trajectory is modeled as a cluster graph by VIA 2.0 where nodes are PARC-based clusters of single cells. Thus, it seems that the trajectory backbone is formed in the same manner as VIA 1.0. Some recent methods (e.g., MARGARET) have been shown to outperform VIA 1.0 for different trajectory topologies. It is necessary that the authors perform a systematic benchmarking of the trajectory inferred by VIA 2.0 using gold standard real datasets and/or simulated datasets (see https://www.nature.com/articles/s41587-019-0071-9 for details on the datasets as well as evaluation metrics) to understand the improvement of VIA 2.0 over VIA 1.0 in capturing different trajectory topologies. The performance should be compared against other TI methods that use graph partitioning (e.g., PAGA, MARGARET) as well as recently developed methods such as Monocle 3.*

R2.Q1. We thank the reviewer for their suggestion to consider other methods that have also been developed with the aim of achieving complex trajectory inference. One of the key advances in StaVia is the integration of scRNA-velocity, temporal, and spatial information in the TI framework. We originally chose to benchmark against CellRank+PAGA+scVelo because these three methods combined can incorporate both pseudotime and scRNA-velocity based information while interfacing on both a cluster-graph and single-cell resolution for relatively large scale datasets (~100K cells). Whilst for Via 1.0, we benchmarked extensively on several state-of-the art TI methods (Monocle3, Palantir, PAGA, SlingShot and STREAM) on datasets without any scRNA-velocity/spatial/temporal elements on both synthetic and gold standard datasets by following the assessment framework used in Saelens et al., 2019, we refrained from repeating this for StaVia as these methods are not designed to handle the multi-modal views presented by recently emerging atlases. However, in order to represent the class of methods that cannot incorporate metadata of any kind, we have extended the comparisons to include Palantir, which is one of the most competitive and scalable methods of this kind. The comparisons of StaVia with and without memory also demonstrate the improvement in StaVia, over VIA 1.0. We also attempted to use MARGARET [Pandey & Zafar 2022] which offers a cluster graph and single-cell view,  but ran into issues of RAM (due to large matrix computations of pseudotime) and runtime (>3 hours for the first step using GPU) on our atlases which are at least ~90K cells.

Our revisions to highlight the comparative strengths of StaVia include 1) a new section for using StaVia on spatial-temporal atlases with results compared to Palantir, Paga and CellRank. 2) Additional comparisons on the main time-series atlases with Palantir which showed poor recovery of lineage pathways and gene trends even when manually assigning root and terminal states, possibly due to the lack of being able to incorporate scRNA-velocity for inferred transitions or leverage the temporal labels for aligning the graph with the biological chronology of different time samples. 3) Added quantitative comparisons on the formulation of higher-order random walks with memory in pathway recovery. And 4) New analysis of a very large 8 Million cell time-series dataset to emphasize StaVia’s scalability. These key additions are further described  below:

1. To highlight the competitive performance of StaVia we have now added a new section for using StaVia on spatial-temporal atlases which showcases the predictive power compared to Palantir, PAGA and CellRank. We did this by extending StaVia’s framework to incorporate spatial tissue information which aids in the identification of clusters and connectivity based on gene-expression as well as tissue location. We present an analysis of StaVia on the spatial MERFISH mouse hypothalamus and the spatial-temporal Stereo-seq Zebrafish (ZESTA) data that cannot be replicated by other methods which lack a framework for using spatial coordinates in TI. A comparison to Cellrank, PAGA, Palantir and StaVia on Zesta, demonstrates how StaVia is better able to capture chronology, predict cell fates and suggest meaningful differentiation steps with intermediate populations. Our work is a proof of concept that spatial transcriptomics data can be used in its own right to infer trajectories. This is in contrast to current approaches where a ‘companion’ scRNA-seq experiment is conducted in parallel, to provide the basis on which the clustering, annotation and TI is performed, with the intention of projecting these scRNA-seq based results (annotations, cell fates) onto the spatial-omics data for visualization in a spatial context. We used StaVia to show that the spatial-omics data can be used to infer biologically accurate conclusions about the process/organ being examined, rather than as a visualization platform for results drawn from scRNA-seq data.  These results are discussed in conjunction with the new Fig.9:

***Spatio-temporal cartography in StaVia captures relationships between cells across space and time***

*Spatial omics have expanded our understanding of tissue architecture by mapping cells in their native environments, considering both their physical locations and gene expression profiles. Yet, it remains challenging to truly integrate this spatial information with gene expression profiles, resulting in analyses remaining purely in the transcriptomic domain with resulting cluster annotations and observations subsequently merely being visually projected back onto the spatial tissue locations. This makes integrative spatial and gene expression analysis non-trivial as it is now known that cell clusters or subtypes can exhibit stark contrasts in their distribution across a tissue slice, depending on their microenvironmental neighborhood, be it highly localized or dispersed.*

*While our examples have until now focused on temporally varying processes, we show that StaVia can also be used to investigate spatial datasets to understand cellular landscapes based on a combination of their expression levels as well as characteristics of their spatial “habitats”. As a proof of concept, we use the pre-optic mouse hypothalamus dataset based on MERFISH (multiplexed error-robust fluorescence in situ hybridization) [Moffitt 2018] and a spatiotemporal Stereo-Seq zebrafish gastrulation atlas ZESTA [C.Liu 2023] to show that incorporating these spatial differences, in conjunction with gene expression, when clustering and capturing the connectivity-landscape elucidates differences in cell type and function. Here, StaVia's graph construction leverages a recent concept [V. Singhal 2024] to recalibrate gene expression by considering a cell's environment. Furthermore, StaVia also augments the gene-expression based KNN graph with spatial neighbors when establishing cluster connectivity (See Methods). Hence, the StaVia graph unifies gene expression with the spatial reality of the tissue.*

*In the hypothalamus dataset, this approach yields several key results that distinguish it from the PAGA graph (****Supplementary FigS19a)*** *as well as earlier versions of Via which did not leverage the spatial information: 1) the StaVia graph automatically generates and arranges clusters not purely based on their expression based cell type, but also their general tendency to occupy particular regions of the tissue section. While clusters themselves remain pure in terms of major cell classification, their cluster-level neighbors are often from a shared tissue ‘habitat’. The automated zoning of the tissue into neighborhoods of groups of clusters by the spatially aware cluster graph facilitates hypothesis generation and identification of potentially interesting sites in a tissue where different cell types are colocalized and potentially interact to yield location-specific functions. In the StaVia cluster graph* ***(Fig. 9a)*** *we use the nomenclature of tissue sub-regions [Paxinos & Franklin 2019]* ***(Fig. 9b)*** *to roughly guide the reader regarding the identified StaVia zones. Cells found in the lower section of the tissue slice are generally placed lower in the StaVia graph, whilst those towards the ACA and PVA are found in the top of the StaVia graph. 2) StaVia identifies sub-types that are missed when omitting spatial information. The excitatory neurons are separated into multiple subtypes that are located within their respective zones on the cluster graph but also express distinct DEGs* ***(Fig.9c)****. For instance, the Oxytocin positive Excitatory cluster C42 is placed near the Ependymal cluster C9. Oxt neurons are known to be found near ependymal cells [Jurek & Neumann 2018], this linkage is not predicted when spatial information is left out of the computational analysis; both the oligodendrocyte and astrocyte population comprise two subtypes that present different spatial and DEG characteristics; and notably the sub-population originally annotated as “ambiguous” seems to actually comprise of both inhibitory neurons (Cluster C6 expressing Gad1 I [Krishnan V, 2022]) and Oligodendrocytes (C24 and C15 clusters, expressing Mbp and Ermin [Brockschnieder, 2006]). The PAGA* ***(Supplementary Fig.S19a)*** *graph is an example of a graph that does not relay very much spatial information, given the lack of a framework to incorporate this, and has a more limited set of sub-populations even when graph and clustering parameters are adjusted for to increase the resolution, thus failing to achieve the results outlined above by StaVia.*

*We next use ZESTA [C.Liu 2022], an STOmics (spatiotemporal multi-omics) atlas of Zebrafish gastrulation from 5hpf to 24hpf of 150,000 spots approaching single cell size at a resolution of 10x10x15 micrometers. ZESTA exemplifies the emerging trend of STOmics atlases on which StaVia can demonstrate its unique capability of integrating both spatial tissue coordinates and real-time information in its trajectory analysis (****Fig. 9d-f****). StaVia arrives at a biologically sound representation of the chronology of germ layer differentiation in the first 24 hours of Zebrafish development purely using the Stereo-seq data, without using supplementary scRNA-seq data for the clustering or TI calculations.*

*This is in contrast to current analytical approaches for STOmics data [C.Liu] where the inferred trajectory is computed on a complementary scRNA-seq dataset, and the results subsequently projected onto the spatial data for visualization. We ran StaVia exclusively on the StereoSeq STOmics data and showed that the specification of germ layers into various cell types is captured correctly. The StaVia graph* ***(Fig.9d)*** *shows the progressive commitment of pluripotent cell types towards different cell fates. Edges from early cells in each of the major tissue types are correctly connected to cells of the same tissue type of later stages (this is not the case in the PAGA graph* ***Fig.S19b*** *where cells/clusters are predominantly connected by stage and not cell type). The clusters identified along the StaVia cluster graph as contributing to a lineage pathway are circled on the cluster graph and also colored directly on the tissue slices (****Fig.9e****). The probabilities of cells moving towards a particular fate are also shown at each time point (****Fig.9f****). These two subfigures thus* *highlight the areas on each slice at different time points that are predicted by StaVia to contribute towards the cell fate under consideration, showing progressive commitment of cells as it occurs on the tissue.*

*When running StaVia without the spatial information, but using only the temporal information, we see two main shortcomings* ***(Supplementary Fig.S20):*** *1) earlier time points are not as well divided into different constituent cell types, which limits our ability to detect cell fate specification and cues at earlier stages and 2) certain sub-types are only delineated in the presence of spatial information, e.g. spatial information helps identify different clusters for the anterior and ventral spinal cord, which are distinct both in location and gene expression.*

*Comparison of StaVia with the PAGA graph (****Supplementary Fig.S19b****), as well as CellRank and Palantir (in terms of the inferred fate probabilities) ((****Supplementary Fig.S19c****), further highlight StaVia’s strength in identifying sequential steps and intermediate stages. The PAGA graph does not accurately position the early time points (3 and 5 hpf) in relation to the other clusters. Moreover, because there is limited connectivity between cells of earlier and later cells from the same tissue type, there is scant information on how and which cells progress towards tissue specialized fates. CellRank and Palantir were unable to detect any intermediate states, as evident in* ***Supplementary Fig.S19c*** *for three different cell fates. Even when the resolution of the method was increased (which incurred a high runtime cost in CellRank), it only led to an increase in the number of predicted final states (several of these were not from 24 hpf despite using the same root cell information as StaVia), but did not result in the identification of the relevant transition populations and differentiation pathway between 3hpf and 24 hpf. By contrast, StaVia manages to identify cells from each developmental time that contribute to the final cell fate at 24 hpf* ***Fig. 9e,f****, demonstrating the unique capabilities of StaVia in trajectory analysis.*

***Fig. 9 Spatiotemporally aware StaVia cartography.*** *(a) StaVia cluster graph of mouse hypothalamus preoptic region at bregma -0.289mm. Clusters located in similar zones share spatial ‘habitats’ Clusters are colored by cell type composition, all subplots share a legend for cell type coloration. Scatter plots placed near zones in the graph show the placement of cells in a cluster according to their spatial location on the tissue slice. (b) MERFISH tissue slice at -0.289mm colored by the cell type annotations originally found by Moffitt et al., BNST, bed nucleus of the stria terminalis;   MPN, medial preoptic nucleus  PVA, paraventricular thalamic nucleus;  ACA, anterior commissure;  PE, periventricular hypothalamic nucleus  LPO, lateral preoptic area  MPA, medial preoptic area.  (c) the top DEGs show clear differences in corresponding subpopulations (d) StaVia cluster graph of the trajectory, colored by major cell type. Cell and cluster annotations are made of StaVia’s clusters using the DEGs and markers compiled by C. Liu et al.,  for each cell type (e) progression of key cell fates for 10-hpf onwards (since 3-to-5-hpf are a common origin for all cell fates) based on cluster-connectivity in the StaVia graph. Colored regions in (sub-fig e) correspond to labeled clusters in the cluster graph (sub-fig d). Lowest row shows the normalized gene expression for each cell fate by a known marker gene (See* ***Supplementary Table 3d*** *for literature references of cell type markers). (f) lineage probabilities towards three cell fates colored onto the tissue slices to show the propensity of cells on the tissue in space-time of developing towards a particular fate (****see Supplementary Fig.S20c*** *for Palantir and CellRank lineage probabilities.*

***Fig.S19 Comparison to PAGA, Palantir and CellRank for Spatial data*** *(a) PAGA of the MERFISH cells without integrating spatial information. (b) PAGA graph of ZESTA spatial-temporal Stereo-Seq dataset colored by the major cell type of each cluster. Blastoderm 3hpf (purple) and YSL-5hpf cells (light blue) are entirely separated from the rest of the 10hpf-24hpf cells which comprise the three connected subnetworks (c) the single-cell lineage probabilities for 3 example terminal cell fates in ZESTA predicted by StaVia, CellRank and Palantir. CellRank and Palantir cannot identify any relevant populations in the 10-18hpf time points that contribute towards the final cell fate. We have to manually predefine most of the cell fates in Palantir in order to compare lineage probabilities.*

***Fig.S20 StaVia for Zesta - with and without spatial coordinates*** *(a) StaVia without spatial information resolves fewer early stage cells into cell type specific clusters. There are for example only 2 magenta 8hpf clusters which combine the early neural keel and segmental plate and the yolk syncytial layer (b) StaVia without spatial information, colored by cell type. In (c-d) StaVia uses the spatial information and, these three cell types are separated into 3 main clusters at the early 8hpf (magenta) color stage clusters. (d) is colored by cell type and highlights three clusters that have distinct spatial habitats for the spinal cord cell type.*

1. **Incorporation of temporal and scRNA-velocity information to improve prediction accuracy of lineage pathways and development.** As mentioned before, most current computational tools do not incorporate these types of additional metadata. In addition to the CellRank+PAGA comparisons (which also allow the integration of scRNA-velocity and pseudotime on both cluster-graph and single-cell resolution) for automated cell fate and lineage probabilities, we have now included comparisons with Palantir (as a popular, scalable pseudotime and random walk based method) for all gene-trends and lineage pathways in the Mouse and Zebrahub datasets, see **Supplementary Fig.3 and Supplementary Fig.7f**. We had to manually assign cell fates (indicated by “preset” label in Fig.S3 below) to facilitate comparison in these datasets as Palantir was unable to detect relevant cell fates in these large atlases. Its recovery of intermediate stages and pathways even when provided root and terminal states was often poor, possibly suffering from the challenge of chaining multiple time-points across lineages together and inferring transitions without any guidance from existing temporal labels, RNA velocity or memory.

***Supplementary Fig.S3 Lineage paths and gene trends comparison between StaVia, CellRank and Palantir.*** *StaVia’s lineage probabilities show paths from start-intermediate-end states, with the single-cell probabilities towards a given cell fate being visualized on the Atlas View. CellRank and Palantir lineage pathways (visualized on a UMAP embedding) are either very localized and show little information on the end-to-end progression from epiblast along consecutive stages, or are very diffuse (Gut), such that several cell populations are shown to progress towards the gut. Asides from not conveying the correct transitions, another consequence of this is that the gene trend lines associated with lineages are not correctly upregulated. For example, the Brain lineage trend line is light blue in CellRank (indicated by the border color of the pathway plot). However, the light blue gene trend line is not the most upregulated for Pax6 or Fez1 and hence receives a cross-mark in the columns titled “specificity”. scVelo’s latent time used for gene trends takes 105 minutes to compute on 8 cores and requires 99 GB RAM, compared to StaVia’s computes in ~ 1 minute and requires less than half the peak RAM.*

***Supplementary Fig.S7. Zebrahub mesoderm: Comparison of lineage paths and gene specificity trends*** *(a) End-to-end lineage probabilities for automatically detected mesodermal cell fates in StaVia at Memory =10. All mesodermal-lineage gene trends are plotted, but only the lineage for which the marker gene is relevant, is upregulated in each case - indicated by the green tick mark which means the color of the trendline matches the color of the associated gene name. (b) In order to facilitate comparison,* ***we manually assign cell fates for CellRank which does not detect all relevant fates.*** *CellRank’s pathways projected on the UMAP used in the Zebrahub paper and made publicly available (Anndata object with velocity) (b-i) shows the gene trends for the same mesodermal lineages (we manually assign those that are not detected by CellRank in order to compare all cell fate pathways) when CellRank’s lineage probabilities are used together with scVelo’s latent time. (b-ii) Gene trends for CellRank when using the DPT pseudotime  (c) Similar analysis for Palantir which required manual setting of cell fates*

1. **Improved comparisons for the use of memory in the formulation of higher-order random walks in pathway recovery:** In addition to qualitatively comparing the outputs of marker genes and single-cell differentiation probabilities predicted by StaVia to those predicted by CellRank, Palantir and StaVia without memory for all lineages in the mouse gastrulation (**Supplementary Fig.S3**) and all mesodermal lineages of Zebrahub (**Supplementary Fig.S4, and Supplementary Fig.S7**), we have now also added a quantitative measure of this improvement in gene trend separation and distinction between lineage pathways in **Supplementary Fig.11**. Please refer to R2.Q3 for details.
2. **Scalability for large cell counts** - StaVia is designed to process large scale atlases whose complexity is characterized both by the sheer cell count as well as the plurality of co-occurring lineages. This demands efficiency in terms of runtime and memory usage. Via 1.0 was tested for runtime and memory usage, and compared to methods that could also be used on large cell counts. The framework for metadata integration, visualization and memory-based random walks in StaVia have since been carefully designed to limit the increase in overhead. To highlight the continued speed and efficiency of StaVia, we showcase its scalability on the 8 Million cell scRNA-seq gastrulation to mouse pup dataset which we have added in **Fig.8e**. The TI required 3.5 hours and was not attempted on other TI methods as they are significantly slower even on much smaller datasets of ~100K. While CellRank and Palantir are slower and more computationally intensive than StaVia, they can still analyze Zebrahub and Murine Gastrulation atlases ~100K cells within a reasonable amount of time (<30mins, compared to <5 mins for StaVia). However, for methods like Monocle3 and MARGARET these mid-sized atlases become intractable even after several hours. We note that MARGARET when attempted on the 150K samples ZESTA dataset on a GeForce GTX 1080 Nvidia GPU takes 3 hours to compute the first step of its computational pipeline, and then produces a segmentation default when proceeding to calculate the pseudotime required for trajectory directionality, due to high RAM requirements on a large matrix computation. We raised this issue many weeks ago, but have not yet heard back regarding the feasibility of running these atlases. Visualization times are compared across several visualization methods for the 120K Zebrahub dataset and shown in **Supplementary Fig.S12b**. Given the runtimes reported in **Fig.S12b,** it was to be expected that we were only able to successfully run StaVia and UMAP to completion on the 8 Million cell Mouse to Pup atlas, taking 13 and 18 hours respectively.

*(Fig.8e) 8 Million cells of mouse gastrula to pup (E8.0 to E18.0) [C. Qiu 2023] by StaVia and UMAP colored by major tissue type and developmental stage. PHATE, t-SNE and force-directed layouts were attempted on this mega atlas but failed due to errors even after 24-48 hours of runtime and over 30 cores of parallel processing.*

*(Fig.S12b) Comparison of runtime (secs) to compute single cell embeddings for Zebrahub (120K cells).*

*R2.Q2. In the VIA 2.0 TI graph for mouse gastrulation (Fig. 3a), some edges are observed from hematopoietic lineage towards paraxial mesoderm/mesenchyme. Can the authors describe these edges? Are these supported by previous literature?*

R2.Q2. We thank the reviewer for their question surrounding the significance of the transitions leading to and from the hematopoietic branch. We refer to **Figure.3a** (below) with four main edges that connect clusters in the hematopoietic lineage to the mesodermal lineage, labeled A, B, C and D so we can refer to them more easily in our explanations. Edge A represents the first wave of hematopoiesis where cells from the nascent mesoderm produce primitive erythroids [L.Lange & Morgan 2021, J.Palis 2014]. Edge B is a minor edge where nascent mesoderm cells are redirected towards the main mesodermal branch instead of proceeding towards commitment in the hematopoietically specified branch. Edge C, extends from the hemato-endothelial clusters towards the hematopoietic branch and represents the onset of definitive hematopoiesis initiated by hemogenic endothelial cells [Wu Y & Hirschi KK. 2020]. Edge D extends from the hemato-endothelial clusters towards the paraxial and lateral plate mesoderm and might represent endothelial-mesenchymal transitions (EndMT) of non-hemogenic endothelial cells towards the development of the arteries, heart and vasculature [Arciniegas 2005, Kovacic 2019]. New **Supplementary Note 2** includes an explanation of the observations provided above.

*R2.Q3. The usage of memory in the formulation improves the gene trends as compared to other methods such as CellRank. However, these improvements are shown qualitatively. Is it possible to show quantitative improvements using known lineages?*

R2.Q3. We agree with the Reviewer that a quantitative measure can be a useful way to summarize the benefits of using random walk with memory in better separating the pathways to different lineages. In the main text we show a qualitative assessment of the impact of memory as seen in **Figure 4** where StaVia is run with differing levels of memory. We have now tried to quantify this improvement **(see Supplementary Fig. S11a-d** below**)** and compare it with other methods (**see Supplementary Fig.S7a-c and Fig. S11d** below) by using the area between the gene trend of interest and other lineages’ trends as a quantitative measure of how well the memory formulation improves the ability to capture separation of lineages as they emerge. This provides a useful way to quantify the gradual improvement in StaVia as we increase from no memory to mid and higher levels of memory. However, quantifying this area is only reasonable when CellRank and Palantir can upregulate the correct marker gene along pseudotime. This is unfeasible for Palantir since the lineage trends are more distorted (see **Supplementary Fig. S7c** for which we demonstrate the qualitative comparison exhaustively across all mesodermal cell fates in Zebrahub). We note that for both CellRank and Palantir, many cell fates need to be manually provided as they are not detected automatically. The same start and end states are therefore provided to all methods in order to focus on the differences arising in the pathways, rather than measuring the ability to detect appropriate start and end states.

***Fig.4 StaVia Memory impact on lineage paths*** *(a) Increasing memory mitigates too-diffuse pathways from epiblast towards specialized cell fates and consequently improves the associated gene trends specificity as shown in (b). Gene expression trends along pseudotime for lineages NMP (grey), PSM (green), Brain (pink) and Neural Crest (NC) (peach) . Hoxb9 is an NMP marker and we expect the grey NMP gene expression to become comparatively more upregulated than the other three lineages. Similarly we expect Meox1 as a PSM marker to be comparatively more upregulated.*

***Supplementary Fig.S7. Zebrahub mesoderm: Comparison of lineage paths and gene specificity trends*** *(a) End-to-end lineage probabilities for automatically detected mesodermal cell fates in StaVia at Memory =10. All mesodermal-lineage gene trends are plotted, but only the lineage for which the marker gene is relevant, is upregulated in each case - indicated by the green tick mark which means the color of the trendline matches the color of the associated gene name. (b) In order to facilitate comparison,* ***we manually assign cell fates for CellRank which does not detect all relevant fates.*** *CellRank’s pathways projected on the UMAP used in the Zebrahub paper and made publicly available (Anndata object with velocity) (b-i) shows the gene trends for the same mesodermal lineages (we manually assign those that are not detected by CellRank in order to compare all cell fate pathways) when CellRank’s lineage probabilities are used together with scVelo’s latent time. (b-ii) Gene trends for CellRank when using the DPT pseudotime  (c) Similar analysis for Palantir which required manual setting of cell fates*

***Supplementary Fig S11. Impact of Memory on Mesodermal Gene Trends (Zebrahub):*** *(a) When the random walk has no memory (is first order), we see that the pharyngeal and fin lineages have significant cell-fate probabilities in the non-mesodermal middle and lower branches, indicated by the light purple-pink coloration in these lower branches. As memory is increased the middle and lower ectoderm branch become a darker blue and the cells residing on them do not express a likelihood towards the mesodermal lineages. Including random walks with memory therefore helps refine the end-to-end pathways to cell fates. (b) The predecessor genes are correctly shown to all be downregulated along the pseudotime axis. (c) the graph shows the lineage separation in StaVia at no memory (denoted by mem=1) and higher levels of memory (mem=10, 100). As memory increases the separation for all 5 mesodermal lineages is more pronounced. The separation is measured as the ratio of the average area between the gene trend whose lineage is associated with that gene and other lineage trends for that gene, versus the total area under the lineage-of-interest’s gene trend. (d) StaVia lineage separation compared to CellRank. The ‘X’ marks instances where the incorrect lineage is most upregulated as seen in Fig.S7b where CellRank’s trends are plotted. Palantir’s trends are not quantified as the gene trends are very distorted (as seen Fig.S7c)*

*R2.Q4. The authors should cite relevant TI methods/approaches which also model cell state and cell fate dynamics (e.g., Palantir, MARGARET, FateID).*

R2.Q4. We thank the authors for this comment and have made the appropriate changes within the introductory paragraphs of the text.

*“Available TI methods face three pressing challenges, the first is the inability to resolve end-to-end differentiation pathways that preserve localized details of underlying trajectories while maintaining a global view of their connectivity, resulting in differentiation pathways for distinct lineages being intermingled (by deviating into unrelated intermediate cell populations) or too myopic (failing to detect transition states). The use of first-order memoryless random walks employed by most TI methods (e.g. Palantir [Setty 2019], MARGARET [Pandey & Zafar 2022], CellRank [Lange 2022], Via 1.0 [Stassen 2021]) make them susceptible to these misleading pathways. The second is that strategies to integrate available metadata (e.g spatial or temporal information) that could aid in the analysis of the cellular landscape are not readily available, thus forgoing the opportunity to use these sources of complementary information. Third, current practices to visualize developmental landscapes rely on established dimension-reduction visualization tools which primarily capture clusters of distinct lineages (e.g. UMAP [McInnes 2018], t-SNE [Maaten & Hinton 2008]). These tools are not designed to display a single-cell embedding that can intuitively be mapped or linked to inferred continuous trajectories. On the other hand, methods relying on diffusion maps (e.g. Phate [Moon 2019]) convey progression information at the expense of collapsing/superimposing multiple distinct lineages.*”

**2^nd^ round**

**Reviewer 1**

The authors have addressed my previous question, and the paper has improved.

**Reviewer 2**

I thank the authors for meticulously addressing all the points raised by the reviewers. I do not have any major comment.

Since the name of the method is now changed to StaVia (earlier it was VIA 2.0), the authors need to make sure that the method name is consistently updated in all the figures and text. I noticed that 'VIA 2.0' was being used in supplementary figure S9. The authors should correct it and any other inconsistent terminology.
